# A tyrosine kinase-STAT5-miR21-PDCD4 regulatory axis in chronic and acute myeloid leukemia cells

**DOI:** 10.18632/oncotarget.19192

**Published:** 2017-07-12

**Authors:** Anne-Sophie Espadinha, Valérie Prouzet-Mauléon, Stéphane Claverol, Valérie Lagarde, Marc Bonneu, François-Xavier Mahon, Bruno Cardinaud

**Affiliations:** ^1^ University of Bordeaux, INSERM U1035, Bordeaux, France; ^2^ University of Bordeaux, INSERM U1218, Bordeaux, France; ^3^ University of Bordeaux, Plateforme Protéome, CGFB, Bordeaux, France; ^4^ Bordeaux Institut National Polytechnique, Bordeaux, France

**Keywords:** CML, STAT5, miR-21, microRNA, leukemia

## Abstract

MicroRNAs (miRNAs) are regulators of several key patho-physiological processes, including cell cycle and apoptosis. Using microarray-based miRNA profiling in K562 cells, a model of chronic myeloid leukemia (CML), we found that the oncoprotein BCR-ABL1 regulates the expression of miR-21, an “onco-microRNA”, found to be overexpressed in several cancers. This effect relies on the presence of two STAT binding sites on the promoter of miR-21, and on the phosphorylation status of STAT5, a transcription factor activated by the kinase activity of BCR-ABL1. Mir-21 regulates the expression of PDCD4 (programmed cell death protein 4), a tumor suppressor identified through a proteomics approach. The phosphoSTAT5 — miR-21 — PDCD4 pathway was active in CML primary CD34^+^ cells, but also in acute myeloid leukemia (AML) models like MV4.11 and MOLM13, where the constitutively active tyrosine kinase FLT3-ITD plays a similar role to BCR-ABL1 in the K562 cell line.

## INTRODUCTION

In chronic myeloid leukemia (CML), the activity of the constitutively active tyrosine kinase BCR-ABL1 [the product of the t(9;22)(q34;q11) chromosome translocation arising in hematopoietic stem cells of the bone marrow] drives the activation of the PI3K/AKT, JAK/STAT, and RAS/RAF/MEK/ERK pathways. Among other consequences, activated or inhibited transcription factors induce important modifications of the CML cells gene expression pattern that could impact cell cycle control, apoptosis and genetic instability, leading to the expansion of the oncogene-transformed cells and to the acquisition of potentially harmful *de novo* mutations [1]. However, indirect BCR-ABL1-dependant regulations might also occur, for instance through the action of microRNAs (miRNAs). Among the ~2000 miRNAs reported in humans, numerous species are up- or down-regulated in various cancer models. In the context of CML however, there is no clear consensus regarding the role of specific miRNAs, despite several studies [2, 3].

Here, we studied the effects of a clinically relevant concentration of imatinib, a tyrosine-kinase inhibitor (TKI) that blocks BCR-ABL1, on the BCR-ABL1^+^ cell line K562: both the microRNA expression profile and the cells proteome were analyzed. Using microarray hybridization, RT-qPCR experiments and a functional assay, we identified miR-21 as one of the most significantly down-regulated microRNA in cells that were treated with imatinib. In parallel, a semi-quantitative proteomic approach identified the tumor suppressor *programmed cell death protein 4* (PDCD4) as the most over-expressed protein in imatinib-treated cells. We showed that miR-21 can bind to PDCD4 3'UTR and decrease its expression. The STAT5 - miR-21 - PDCD4 pathway was conserved in CML primary CD34^+^ cells, and to some extent in acute myeloid leukemia (AML) models as well; the known functions of miR-21 and PDCD4 suggest that their regulation by BCR-ABL1 could participate in the antileukemic response triggered by tyrosine kinase inhibitors.

## RESULTS

### Imatinib treatment induces the significant regulation of 13 microRNAs in K562 cells

**Figure 1:**
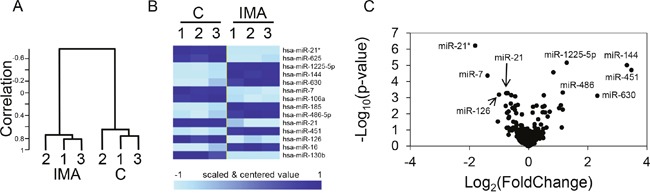
Regulation of miRNA expression by imatinib in K562 cells Cells (n=3 independent wells for each sample) were either not treated (C) or treated for 24 h with 1 μM imatinib (IMA) before RNA extraction, labeling and miRNA microarray hybridization. **(A)** Hierarchical clustering of the 6 independent microRNA expression profiles, revealing a strong molecular signature associated with the treatment. **(B)** Heatmap of the 13 significantly (p<0.001) dysregulated miRNAs. **(C)** Significativity (y axe) versus fold change (x axe) plot; the most relevant candidates are depicted.

K562 cells were treated for 24h with 1 μM imatinib, a condition that induced apoptosis in less that 5% of the cells, as revealed by annexin V labeling (not shown). The treatment induced significant changes in the microRNA expression profile (Figure 1): a hierarchical clustering clearly ranked the samples according to the treatment (Figure 1A), revealing an overall modification of the miRNA expression profile. The 13 miRNAs that were significantly (p<0.001) dysregulated are listed on Figure 1B, altogether with a heatmap illustrating that the imatinib-induced effects concerned both up- and down-regulations. A subset of the microRNAs that are down- or up-regulated and the associated p-values are depicted on Figure 1C. Five out of the seven up-regulated microRNAs revealed the previously described TKI-induced erythroid differentiation of K562 cells [4]: miR-144 and miR-451 are produced from the same pri-miRNA and are mostly expressed in the erythroid lineage where they participate in the late stages of erythropoiesis regulation [5]; miR-486 expression is also increased during erythroid differentiation [3]; miR-185 and miR-16 expression correlate with the appearance of erythroid surface antigens (CD71, CD36, and CD235a) and hemoglobin synthesis in cord blood-derived CD34^+^ cells [6]. The down-regulated miRNAs were miR-21 and its passenger strand miR-21*, miR-625, miR-7, miR-106a, miR-126 and miR-130b. The regulation of miR-126 might also be a signature of the TKI-induced erythroid differentiation since this microRNA inhibits the erythropoiesis in CD34^+^ cells [7]. Besides miR-625, that was expressed at very low levels in the K562 cells and has probably no regulatory functions, the other down-regulated microRNA were not previously associated with erythroid differentiation, but rather with distinct steps or models of tumorigenesis. MiR-21 was specifically interesting for several reasons. First, it is known to be overexpressed in many solid tumors and is considered as a *bona fide* “oncomicroRNA” [8]. Second, in our experiments, it is highly expressed in non-treated K562 cells, thus suggesting regulatory functions, as it is suggested that only the most abundant miRNAs mediate efficient target suppression [9]. Third, the two mature forms miR-21 (miR-21-5p) and miR-21* (miR-21-3p) produced from the same precursor (pre-miR-21) are down-regulated by imatinib treatment (Figure 1C); this reinforce the potential role of the *miR-21* locus in the biology of CML since both miR-21 and miR-21* have specific targets and tumorigenic effects [10] [11]. Finally, the regulation of miR-21 has been rarely studied in the context of myeloid leukemias, strengthening the interest of studying this microRNA in our model.

**Figure 2:**
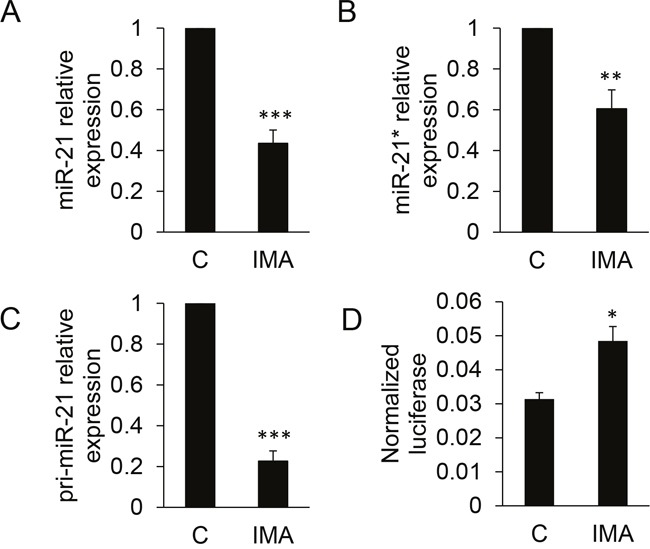
Quantification of miR-21, miR-21* and pri-miR-21 in K562 cells following imatinib treatment Cells (n=8-9 independent experiments) were either not treated (C) or treated for 24 h with 1 μM imatinib (IMA) before RNA extraction, reverse transcription and quantification by qPCR of miR-21 **(A)**, miR-21* **(B)** or pri-miR-21 **(C)**. In **(D)**, cells (n=4 independent experiments) were transfected with a miR-21 sensor plasmid, then treated or not with imatinib. Luciferase activities were measured after 24h. *** P<0.001, * P<0.05 (treated versus not treated cells).

The effects of BCR-ABL1 inhibition on the expression of miR-21 and miR-21* were measured by RT-qPCR. The results confirmed their significant down-regulation following a 24h imatinib treatment (Figure 2). Of note, the mean Ct (threshold cycle) values for miR-21 and miR-21* were 27.3 and 36.4, respectively, in untreated cells. This reflects the low expression level of miR-21* as compared to miR-21 (≈ 500-fold difference), that was already suggested by the microarray hybridization signals intensities (≈ 120-fold difference). The effect of imatinib also concerned the primary transcript of *miR-21*, pri-miR-21, that was quantified by RT-qPCR on RNA extracted from the nuclear fractions of treated- or non-treated cells (Figure 2C). This suggested that the imatinib-induced miR-21/miR-21* decrease relied on a transcriptional regulation of the *miR-21* gene by BCR-ABL1 activity, although the expression of this miRNA is regulated by post-transcriptional mechanisms in other models [12]. The 24h imatinib treatment also produced a significant increase of the luciferase activity expressed by K562 cells transfected with a “sensor” plasmid driving the transcription of a *Renilla* luciferase coding sequence followed by a short 3'UTR containing two miR-21 complementary sequences (Figure 2D); this functional assay confirmed the imatinib-induced decrease of miR-21. Imatinib did not modify the luciferase activity of K452 transfected by a miR-21* sensor plasmid (not shown), suggesting that this microRNA, perhaps because of its very low level of expression, plays no regulatory function in K452 cells. Imatinib had comparable effects on miR-21, miR-21* and pri-miR-21 on another BCR-ABL1^+^ model: the LAMA-84 cell line (Supplementary Figure 1), strengthening the results obtained on K562 cells. On K562 cells, the second-generation dasatinib (at 10nM) and nilotinib (at 20nM) induced a similar effect than imatinib on miR-21 expression (Supplementary Figure 2). Conversely, it did not modify miR-21 expression in BCR-ABL1-negative cell lines: TF1 and HL60 (two acute myeloid leukemia cell lines) and NTERA-2 (a pluripotent human embryonic carcinoma cell line) (Supplementary Figure 3). Finally, we measured miR-21 in RNA prepared from induced pluripotent cells (iPSC) prepared from CD34^+^ primary cells prepared from cord blood, and transduced or not with a lentivirus allowing the expression of BCR-ABL1 (provided by Dr. A. Bedel, INSERM U1035, Bordeaux, France): the BCR-ABL1^+^ cells expressed higher levels (x2) of miR-21 than the BCR-ABL1^-^ cells (Supplementary Figure 4). Taken together, these results suggest that the BCR-ABL1 activity positively regulates the expression of the microRNA miR-21 and of its passenger strand miR-21*, through a transcriptional mechanism.

### STAT5 regulates the expression of miR-21 in K562 cells and is necessary for the imatinib-induced miR-21 regulation

**Figure 3:**
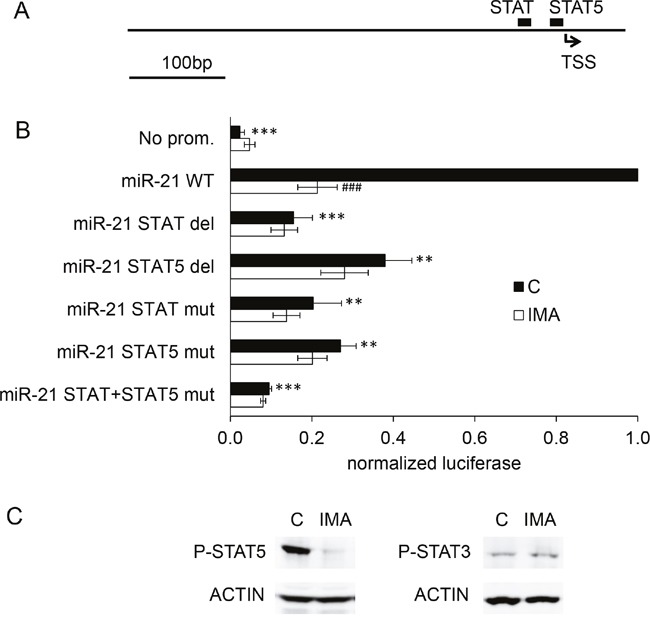
STAT binding sites are necessary for the action of imatinib on miR-21 promoter activity A 517 bp fragment corresponding to the proximal *miR-21* promoter was cloned upstream of a firefly luciferase cds. **(A)** Relative positions of the STAT/STAT5 putative binding sites identified by Genomatix. **(B)** K562 cells were transfected with wild-type (WT), deleted or mutated variants of the miR-21 promoter, and treated or not with imatinib (1 μM, 24h) before luciferase activity measurements. For each independent experiment, the normalized luciferase activities of the deleted/mutated variants were compared to the activity of the WT *miR-21* promoter. **(C)** phospho-STAT5 and phospho-STAT3 were detected by western blot of protein extracts from K562 cells not treated or treated with imatinib. ***P<0.001, ** P <0.01 (variant versus WT miR-21 promoter); ### P <0.001 (imatinib-treated versus not treated cells).

In order to study the mechanism underlying the imatinib-induced regulation, we cloned a 517 bp genomic DNA fragment corresponding to the proximal *miR-21* promoter [13] into the luciferase vector pGL4.10. Luciferase activity expressed by K562 cells transfected with this construct suggested that the 517 bp DNA fragment contains regulatory element(s) necessary for the action of imatinib on the expression of miR-21 (Supplementary Figure 5). Two of the transcription factors binding sites identified in this 517 bp fragment through a bioinformatics analysis (*Matinspector* program of the *Genomatix* suite) concern factors of the *Signal transducer and activator of transcription* (STAT) family (Figure 3A): the first one (TTACAGGAA, position 410-418) is not related to a specific subtype of STAT; the second one (TTCTGAGAA, position 441-449) is qualified as a “STAT5” site by *Matinspector*. Of note, these two sites were qualified as “STAT3” in other studies [14, 13], although they were not specifically associated with this transcription factor by *Matinspector*. Interestingly, STAT5 is known to be regulated by BCR-ABL1 activity in K562, in other BCR-ABL1^+^ cell lines and in CML primary cells as well [15, 16]. STAT3 and/or STAT5 regulate miR-21 expression in mammary epithelial cells and in T cells [17–19]. The two sites (called “STAT” and “STAT5” hereafter) were deleted and/or mutated (Supplementary Figure 6) and the corresponding plasmids were transfected in K562 cells. Luciferase measurements revealed that the deletion of any of the two binding sites (STAT or STAT5) significantly decreased the *miR-21* promoter activity (Figure 3B). Two-nucleotides substitutions in the STAT site, the STAT5 site or in both the STAT and STAT5 sites also sharply decreased the promoter activity. The presence and the integrity of these two sites were also necessary for the action of imatinib on the transcription of *miR-21*: deletions or mutations of the STAT and/or STAT5 sites abolished the imatinib-induced decrease of luciferase activity observed in cells transfected with the wild-type *miR-21* promoter (Figure 3B).

The two sites we identified are supposed to mediate STAT3-dependant transcription in myeloma cells [14]. However, the distinction of STAT3- and STAT5-specific binding sites is not always justified: for instance, STAT3 and STAT5 are able to bind common sites across the locus encoding IL-17 [20]. This seems to be the case also regarding the promoter of *miR-21*, since chromatin immunoprecipitation experiments conducted on cells overexpressing STAT3 or STAT5 carrying FLAG epitopes confirmed that both transcription factors could bind to the *miR-21* promoter (Supplementary Figure 7). However, we hypothesized a role of STAT5 rather than STAT3 in the transcription of *miR-21* for several reasons: the phosphorylation of STAT5 in BCR-ABL1^+^ cells, through the kinase activity of BCR-ABL1 [21], is correlated with the cells growth and viability [22]. Recent works revealed that STAT5 overexpression leads to a TKI-resistant phenotype, an effect that is independent of JAK2 expression, and that targeting STAT5 by drugs, like the neuroleptic drug pimozide [23] might be a promising perspective in the pharmacology of leukemia and other cancers as well. Moreover, STAT5 is expressed at much higher levels than STAT3 in K562 cells (transcriptomic analysis, not shown). To strengthen these indirect arguments, we studied the effect of imatinib on phospho-STAT5 and phospho-STAT3 by western blotting. The results shown on Figure 3C reveal that imatinib induced a near-complete dephosphorylation of STAT5, although, interestingly, phospho-STAT3 seemed not modified.

**Figure 4:**
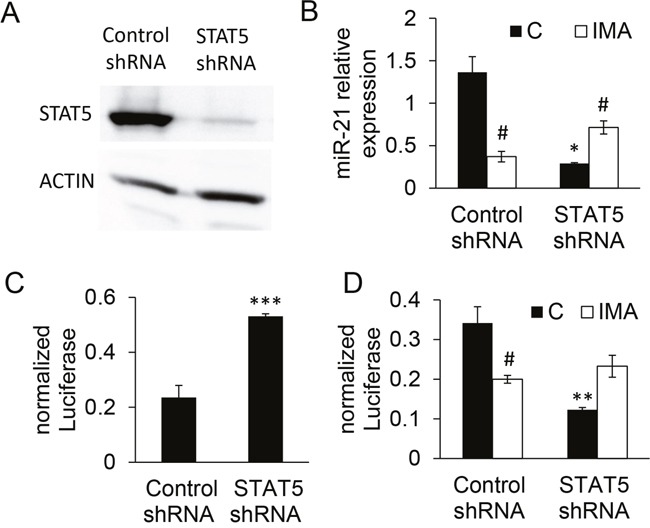
Validation and effects of STAT5 knock down on miR-21 expression in K562 cells Cells were transduced at a MOI of 5 with either STAT5 shRNA or control shRNA lentiviral vector and GFP^+^ cells were sorted 48h later. **(A)** The expression of STAT5 was assessed by western blot analysis, confirming the efficacy of the STAT5 shRNA. **(B, C)** MiR-21 was quantified in the transduced cells either directly by RT-qPCR **(B)** or indirectly **(C)** using a reporter “sensor” plasmid driving the transcription of luciferase cds followed by two miR-21 binding sites. **(D)** The knock-down of STAT5 decreased the *miR-21* promoter activity in cells transfected with the *miR-21* promoter-luciferase plasmid and abolished the imatinib-induced effect. ***P<0.001, ** P <0.01 (control shRNA versus STAT5 shRNA); # P <0.05 (imatinib-treated versus not treated cells).

In order to assess the potential role of STAT5 in the transcription of *miR-21* and in the imatinib effect, K562 cells were transduced with a lentivirus encoding a small hairpin RNA (shRNA) directed against the two isoforms STAT5A/B [24]. This induced a clear down-regulation of STAT5 expression (Figure 4A). The STAT5-shRNA K562 cells significantly expressed less mature miR-21 sequence than the control-shRNA cells, as revealed by RT-qPCR (Figure 4B) and luciferase activity of control- or STAT5-shRNA K562 cells transfected with the “sensor” plasmid (Figure 4C). The activity of *the miR-21* promoter was reduced in STAT5-shRNA cells (Figure 4D). Contrarily to what was observed in control cells, BCR-ABL1 blocking by imatinib could not induce the downregulation of miR-21 in STAT5-shRNA K562 cells, as revealed by RT-qPCR (Figure 4B) and *miR-21* promoter studies (Figure 4D). Instead, it produced a slight up-regulation of miR-21, an effect that might reflect the action of others signaling pathways activated by BCR-ABL1. Taken together, these results suggest that STAT5 mediates the effects of BCR-ABL1 on the regulation of miR-21 in the BCR-ABL1^+^ cell line K562.

### Both miR-21 and STAT5 knock down decrease cell growth and sensitize cells to imatinib-induced cell death

**Figure 5:**
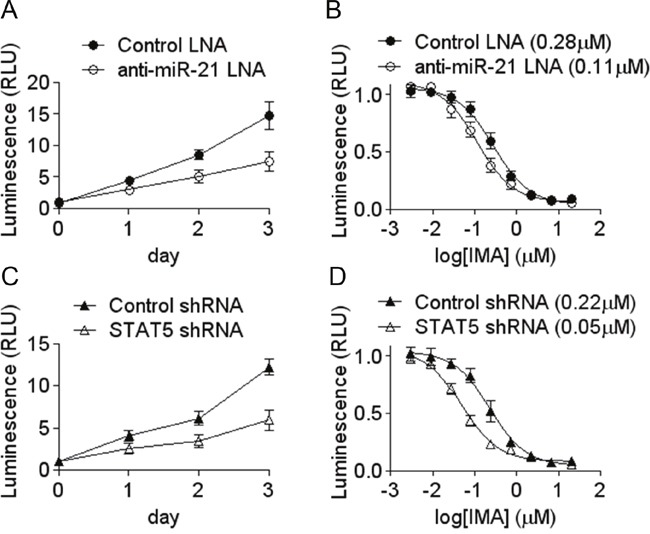
Decreased growth rate and increased sensitivity to imatinib induced by miR-21 and STAT5 knock-down The effects of an anti-miR-21 LNA oligonucleotide **(A, B)** and of STAT5 shRNA **(C, D)** were assessed on the growth of K562 **(A, C)** and on the cells sensitivity to imatinib **(B, D)**. Cells number and viability were measured indirectly by the Cell Titer Glo assay. IC_50_ are shown between parentheses. Data are mean ± s.e. from a representative experiment done in triplicate.

Several studies suggest a functional role of miR-21 in myeloid leukemias: miR-21 is over-expressed in the blastic phase of CML [25]; it is also frequently overexpressed in AML blasts [26]; miR-21 knock down sensitizes CML CD34+ to imatinib [27], K562 cells to arsenic-induced cell death [28], and induces apoptosis of K562 cells [29]. These articles confirm the likely oncogenic role of miR-21 shown by a large number of publications in other models of cancer. In order to strengthen their conclusions, we studied the effect of miR-21 down regulation on K562 cells proliferation rate and sensitivity to imatinib. In parallel, the effects of STAT5 knock down was assessed. The results shown on Figure 5 confirm that miR-21 knock down impairs cell proliferation by ≈50%. It also increases the cells sensitivity to imatinib, as shown by the shift of the IC_50_. Interestingly, these phenotypes are very similar to those produces by the knock down of STAT5, strengthening the possible functional link existing between STAT5 and miR-21.

### PDCD4 expression is regulated by BCR-ABL1 and miR-21

**Figure 6:**
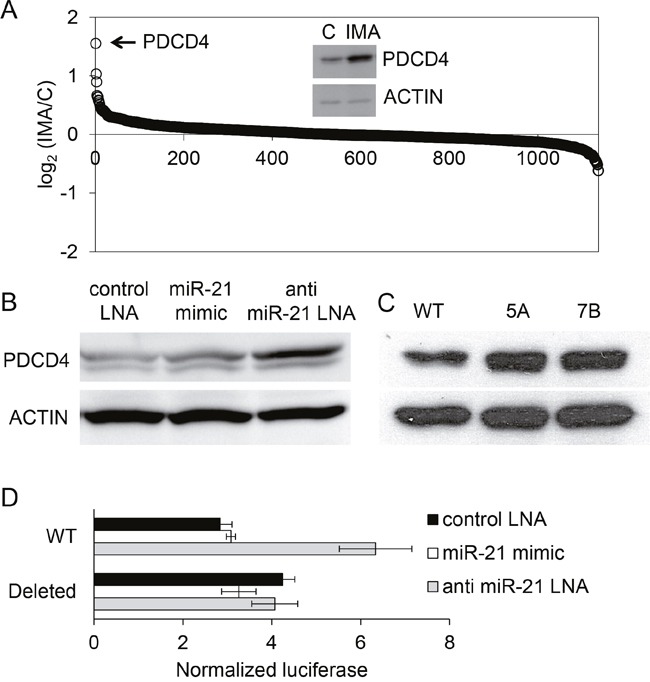
Regulation of PDCD4 expression by imatinib in K562 cells **(A)** The proteome of cells that were not treated (n=2) or treated with imatinib (1 μM, 24h, n=2) was studied using iTRAQ. A total of 1134 proteins were identified and quantified by MS/MS. For each protein (one spot on the graph), the base 2 logarithm of the ratio of its levels in the two samples (IMA/C) is plotted on the y axe. A western blot analysis of PDCD4 expression in control and treated cells confirmed the result observed using proteomics (inset). **(B)** western blot analysis of PDCD4 expressed in cells transfected by a control LNA, a miR-21 mimic or an anti-miR-21 LNA. **(C)** western blot analysis of PDCD4 expressed in normal (WT) or miR-21 KO K562 cells (5A, 7B) **(D)** Luciferase activities of K562 cells co-transfected with (i) plasmids bearing the luciferase coding sequence followed by a 554 bp fragment of the PDCD4 3'UTR containing (WT) or not (Deleted) a miR-21 binding site and (ii) antimiR-21-LNA, miR-21 mimic or control LNA oligonucleotides.

In order to gain insight into miRNA-dependent protein regulations, the proteome of control- or imatinib-treated K562 cells was analyzed by iTRAQ (*Isobaric tags for relative and absolute quantitation*), a semi-quantitative proteomic technique. The results allowed the identification (by 2 distinct peptides or more) and the relative quantification of 1137 different proteins (Supplementary Table 1). Surprisingly, the imatinib treatment (1μM for 24 hours, n=2 independent samples of treated and non-treated cells) induced the over- or under-expression of only a limited number of proteins (Figure 6A). The under-expressed proteins (mean fold-change < 0.66) were the protein kinase LYN, the tubulin beta-2A (TUBB2A) and the neutral amino acid transporter SLC1A5/AAAT. The seven over-expressed proteins (mean fold-change > 1.5) were PDCD4, HBZ, RBM12B, AFG3L2, H2AFX, PKLR and ALDH1A1. Hemoglobin zeta (HBZ) overexpression reflects the erythroid differentiation induced by BCR-ABL1 inhibition in K562 cells and was the only protein found in common in our study and in the proteomic analysis of K562 cells based on the SILAC technique (Stable isotope labeling by amino acids in cell culture) published earlier [30]. The strongest (x2.8) imatinib-induced protein up-regulation concerned PDCD4 (programmed cell death protein 4), identified in our experiments by three unique peptides. PDCD4 is a tumor suppressor protein that acts as an inhibitor of cap-dependent translation by blocking the translation initiation factor eIF4A. PDCD4 inhibits transformation, translation, invasion and intravasation, and its expression is down-regulated in several cancers [31]. Its up-regulation induced by imatinib was confirmed by western blot (Figure 6A, inset) and concerned the *PDCD4* mRNA as well, as revealed by RT-qPCR (×3.9 ± 0.8, n=7, not shown). Since it has been demonstrated in several models that PDCD4 is a characterized target of miR-21 [32], we hypothesized that the imatinib-induced PDCD4 over-expression might rely on the down-regulation of miR-21 we observed earlier.

We artificially up- or down regulated miR-21 concentration by transfecting K562 cells respectively with a synthetic miRNA (miRNA mimic, Ambion) and an anti-miR-21 oligonucleotide (LNA, Exiqon), or with a control LNA. The efficacies of these treatments were checked using cells co-transfected with the miR-21 “sensor” plasmid (not shown). Anti-miR-21 LNA transfected K562 cells expressed a higher level of PDCD4 protein than the control LNA- or miR-21 mimic-transfected cells (Figure 6B). The expression of miR-21 was also artificially abolished by CRISPR/Cas9 gene inactivation [33]: K562 were transfected with a plasmid allowing the expression of a previously validated single guide RNA [10], of Cas9 and of the *pac* gene encoding a puromycin N-acetyl-transferase. After puromycin selection, resistant cells were cloned using methylcellulose, then grown in liquid medium and further analyzed for the presence of mutations in the *miR-21* gene: two out of the seven tested K562 clones had deletions in each of the 3 *miR-21* copies (K562 cells are triploid), as revealed by sequencing (Supplementary Figure 8). The 9 to 36bp deletions, all located in the immediate proximity of the sequence of miR-21, were associated with an absence of miR-21 expression, as shown by RT-qPCR (miR-21 relative expression as compared to WT cells: x0.0001 for clone 5A, x0.002 for clone 7B). A western blot revealed that PDCD4 expression was upregulated in these miR-21 KO cells (Figure 6C), confirming the results of the miR-21 knock-down experiments described earlier.

It is likely that the effects of the anti-miR-21 LNA and of miR-21 knock-out on PDCD4 protein levels relied on a post-transcriptional regulation of *PDCD4* by miR-21: this is suggested by luciferase activity measurements in K562 cells transfected with a reporter plasmid containing a 554 bp fragment of the *PDCD4* 3’UTR placed downstream of a *Renilla* luciferase coding sequence; co-transfection of these cells with the anti-miR-21 LNA induced an increase of the luciferase activity, revealing a regulatory effect of miR-21 on the 3’UTR of *PDCD4* (Figure 6D). The specificity of this effect was verified using a reporter plasmid harboring a deletion of the miR-21 binding site that was previously discovered in the *PDCD4* 3’UTR [32]. Altogether, these results suggest that miR-21 could be an intermediary of the imatinib-induced PDCD4 up-regulation.

### Phospho-STAT5, miR-21 and PDCD4 regulation by imatinib in CD34 primary cells from CML patients and AML cell lines

**Figure 7:**
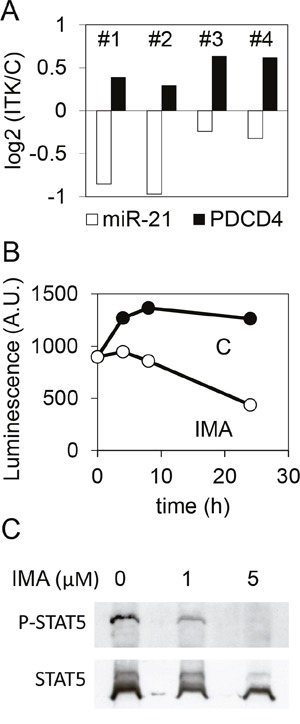
Effect of imatinib on the expression of miR-21 and PDCD4 in CML CD34^+^ cells **(A)** miR-21 and PDCD4 mRNA were quantified by RT-qPCR on CD34^+^ cells prepared from four CML donors (#1 to 4) and treated (5μM, 24h) or not with imatinib. For each CML donor, histograms represent the base 2 logarithm of the ratio (treated/not treated cells). **(B)** CD34^+^ cells from a CML donor were transduced with a lentivirus carrying a firefly luciferase coding sequence cloned downstream of the *miR-21* promoter, then treated or not treated with imatinib. Luciferase activity was measured after 4, 8 or 24 hours. **(C)** phospho-STAT5 and STAT5 were detected by western blot of protein extracts from CML CD34^+^ not treated or treated with imatinib.

In CML, the largest part of the CD34^+^ fraction expresses BCR-ABL1 and is sensitive to imatinib or others TKI. As observed on the K562 cell line, imatinib treatment decreased the expression of miR-21 and increased the expression of *PDCD4* mRNA in CD34^+^ cells prepared from four CML patients (Figure 7A), although these effects were observed at 5μM imatinib (and not at 1μM). It is likely that the effect on miR-21 expression relied on a decreased activity of the *miR-21* promoter, as shown using CML CD34^+^ cells transduced with a *miR-21* promoter-luciferase lentivirus and treated for 0-24h with either vehicle or 5μM imatinib (Figure 7B). Western blot analysis showed that STAT5 was dephosphorylated in the presence of imatinib (Figure 7C), confirming earlier studies [21] and suggesting, as this is the case in the K562 cell line, that phospho-STAT5 activates the transcription of *miR-21* in CML CD34^+^ cells.

**Figure 8:**
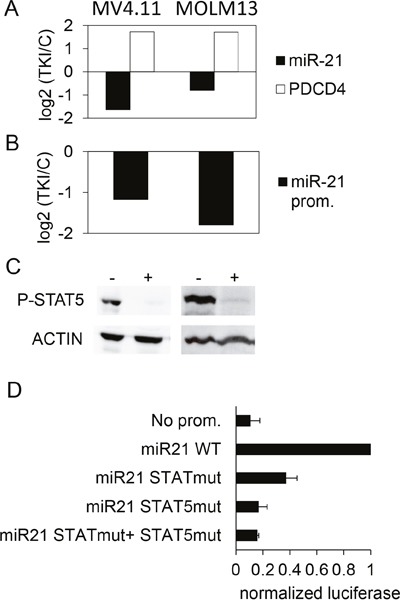
Effect of TKI treatment on the expression of miR-21 and PDCD4 in AML cells lines **(A)** MV4.11 and MOLM13 cells were treated with sunitinib (0.1μM, 24h). Then, PDCD4 mRNA and miR-21 were quantified by RT-qPCR (A), and *miR-21* promoter activity was assessed by luciferase measurements in cells transduced with the *miR-21* promoter-Luciferase lentivirus. **(B)** For each cell line, histograms represent the base 2 logarithm of the ratio (treated/not treated cells). **(C)** phospho-STAT5 levels were assessed in control (-) or TKI-treated (+) MV4.11 (left) and MOLM13 (right) cells. **(D)** Luciferase activity was measured in MOLM13 cells 24 hours after transfection with pGL4.10 plasmids having no promoter, WT or mutated *miR-21* promoters.

STAT5 is activated in acute myeloid leukemia (AML), in particular in the subtypes harboring the internal tandem duplication (ITD) mutations of the tyrosine kinase FLT3 [34]. In parallel, several studies revealed an upregulation of miR-21 across subtypes of AML [35]. It was then tempting to assess the possibility that the STAT5-miR-21 pathway concerns AML models as well. MV4.11 and MOLM13 cells, two FLT3-ITD^+^ cell lines, were treated for 24 hours with 0.1 μM sunitinib, a TKI that blocks FLT3-ITD. This treatment decreased miR-21, increased *PDCD4* mRNA levels (Figure 8A), and decreased *miR-21* promoter activity (Figure 8B). The TKI also produced a dephosphorylation of STAT5 in the two cell types (Figure 8C). MOLM13 were transfected with the pGL4.10 reporter plasmids: as this is the case in K562, the 2-nt mutations introduced in the STAT/STAT5 binding sites strongly reduced the luciferase activity of the transfected cells (Figure 8D). Taken together, these results suggested that the phosphorylation of STAT5, driven directly or indirectly by BCR-ABL1 (in CML) and FLT3-ITD (in AML), could transcriptionally up-regulate the expression of miR-21 and, in turn, contribute to the subsequent regulation of PDCD4, among other miR-21 targets.

## DISCUSSION

MiR-21 is overexpressed in almost all types of human cancers, including leukemia, and has been associated with the regulation of proliferation, growth, invasion and chemoresistance. Its anti-apoptotic properties were previously confirmed in K562 cells [28, 29]. Mir-21 is over-expressed (among other microRNAs) in primary CD34^+^ cells of TKI-resistant patients [36] and in the blastic phase of the disease [25]; it might participate in the TKI-dependent apoptosis of the CD34^+^ CML cells [27]. Using various models, several upstream regulators were shown to participate to the regulation of miR-21 expression, like for instance the histone demethylase RBP2, the androgen receptor, NF-κB, Foxo3a or AP-1 [8, 25]. The activity of factors of the STAT family have also been described: chromatin immunoprecipitation (ChIP) experiments revealed enrichment of STAT3 [17, 14] and of STAT5 [18, 19] in the regulatory regions of *miR-21*. The two STAT binding sites we worked on were previously identified [13, 14] as putative STAT3 sites. Our results extend the regulation of miR-21 by STAT5 to CML, where the role of this transcription factor seems essential, since 2-nt mutations in the binding sites significantly and strongly reduce the activity of the *miR-21* promoter.

In CML CD34^+^, TKI induce a rapid and complete dephosphorylation of STAT5. The effect on STAT3 is much less pronounced, a difference that could rely on the ability of BCR-ABL1 to directly phosphorylate STAT5 [21]. This reinforces the central role of STAT5 in the maintenance of chronic myeloid leukemia [37, 21], in accordance with the known effects of this transcription factor on cell cycle, apoptosis, production of ROS and the sensitivity of CD34+ cells to TKI [37]. Interestingly, these processes are regulated by miR-21 as well [8, 27]. Taking into account the fact that STAT5 activates miR-21 expression, it is tempting to propose that miR-21 could participate in some of the STAT5-dependant regulations in CML.

## MATERIALS AND METHODS

### Cell lines

The BCR-ABL1^+^ cell line K562 and the FLT3-ITD^+^ AML cell lines (MOLM13 and MV4.11) were obtained from the DSMZ (German Collection of Microorganisms and Cell Cultures). They were grown at 37°C / 5% CO2 in RPMI medium (Invitrogen) supplemented with 10% foetal calf serum and penicillin/streptomycin, and split every 2-3 days.

### Patient samples and CD34^+^ cell isolation

Cytapheresis samples from four CML patients were obtained at diagnosis according to the *Etablissement Français du Sang* recommendations. Mononuclear cells were obtained by Ficoll density gradient centrifugation. They were labeled with the StemSep™ Human CD34 Positive Selection Cocktail (Stemcell Technologies) and isolated on MS column (Miltenyi Biotec). CD34^+^ cells were kept for 2-3 days in StemAlpha A medium (StemAlpha) in the presence of Flt3-ligand (50ng/ml), thrombopoietin (50ng/ml) and stem cell factor (50ng/ml). Cytokines were not included during the course of imatinib treatments.

### RNA preparation, miRNA microarray hybridization and RT-qPCR

When prepared for microarray hybridization, RNAs were extracted from Trizol homogenates using a modified protocol: after chloroform extraction and phases separation, 1.5 volumes of 100% ethanol were added to the upper aqueous phase. The mix was deposited onto a RNeasy column (Qiagen), that was further processed according to manufacturer instructions. Cyanine-3 (Cy3) labeled miRNAs were prepared from 0.2 μg RNA using the miRNA complete labeling kit version 2.2 (Agilent Technologies). Labeled miRNAs were hybridized overnight at 55°C onto Human miRNA Microarray V2 (Agilent Technologies) slides. The slides were then washed as recommended by the manufacturer, and scanned on an Agilent G2565CA scanner, at 5 μm resolution and using the 20-bit scan mode. Images were processed with Feature Extraction (version 10.7). Data were quantile-normalized for inter-array comparisons and analyzed using the “BRB-Array Tools” package, version 4.2.0 [38]. Genes that were differentially expressed among the groups were identified using a F-test (Class Comparison Between groups of arrays Package, BRB-Array Tools). Normalized data for all samples have been deposited in NCBI Gene Expression Omnibus and are accessible through GEO Series accession number GSE78037.

For RT-qPCR experiments, RNAs were prepared from Trizol homogenates by precipitation from the aqueous phase following the supplier's recommendations. For RT-qPCR of miR-21 and miR-21*, RNA (200ng) were polyadenylated and reverse-transcribed using the High Specificity miRNA First Strand cDNA synthesis kit (Agilent). After reverse transcription, the PCR reaction was analyzed by real time quantitative PCR (RT-qPCR) using a universal reverse primer (GACGAGCTGCCTCAGTC) and either a miR-21 (TAGCTTATCAGACTGATGTTGA), a miR-21* (ACAGCCCATCGACTGGTGTTG) or a U6 (ATTGGAACGATACAGAGAAGATTAG) forward primer. The cycle thresholds (CT) obtained for miR-21 and miR-21* were normalized using U6 as endogenous gene. For RT-qPCR of PDCD4 and pri-miR-21, the Transcriptor First Strand cDNA synthesis kit (Roche) was used for reverse transcription. The sequences of the primers were TTTTGTTTTGCTTGGGAGGA and AGCAGACAGTCAGGCAGGAT (for pri-miR-21); GAAGGTTGCTGGATAGGCGA and TTGGTAGTCCCCTTCCTTTCC (for PDCD4); TGGAGGGTGTCCGCAATGTT and GAAGGCCTTGACCTTTTCAG (for HUPO, used to normalize the CT obtained for pri-miR-21 and PDCD4). Both types of qPCR (miR-21 and PDCD4/pri-miR-21) were performed with the GoTaq qPCR Master Mix (Promega), using a Biorad CFX96 Real-Time PCR system.

### Plasmids preparation and lentivirus production

The PCR steps were done using Q5^®^ High-Fidelity DNA Polymerase (New England Biolabs). For the miR-21 sensor plasmid construction, two 42-mer oligonucleotides (CCGCTCGAGTCAACATCAGTCTGATAAGCTACTAT CATCAACATCAGTCTGATAAGCTAGCGGCCGCATT CTTAT and ATAAGAATGCGGCCGCTAGCTTATCAGACTGATGTTGATGATAGTAGCTTATCAGACTGATG TTGACTCGAGCGG) were annealed to form a double-stranded adapter containing two miR-21-binding sequences, and that was cloned at the XhoI/NotI of the psiCheck2^TM^ (Promega) downstream of the Renilla luciferase cds. The same strategy was used to construct the miR-21* sensor plasmid with the oligonucleotides CCGCTCGAGACAGCCCATCGACTGGTGTTGCTATCAACAGCCCATCGACTGGTGTTGGCGGCCGCATTCTTAT and ATAAGAATGCGGCCGCCAACACCAGTCGATGGGCTGTTGATAGCAACACCAGTCGATGGGCTGTCTCGAGCGG). The miR-21 promoter (517bp fragment corresponding to the miR-21 studied by Fujita et al. [13]) was PCR amplified from human genomic DNA (Promega) using the primers CGGTTTAACAGCACTGCCTCCA and GTCCTCAGAGTAAGGTCAGCTC, and inserted by ligation in the pGL4.10 plasmid (Promega) downstream of the Firefly luciferase cds. The deletions and mutations of the transcription factors binding sites were introduced by PCR amplification / DpnI digestion of the WT miR-21 promoter pGL4.10 plasmid and sequence-verified. The positions of the deletions and of the mutations are depicted in Supplementary Figure 6. The WT miR-21 promoter was also inserted upstream of the Firefly luciferase cds in a lentiviral vector (lentiviral vector #165 of the Vect'UBfacility, Bordeaux, France). The PDCD4 3'UTR (554 bp fragment corresponding to the positions 1765-2319 of NM_145341) was PCR amplified from human genomic DNA and cloned in the psiCheck2^TM^ plasmid (Promega). The sequence corresponding to the miR-21 seed-binding site (ATAAGCTA) was deleted from the PDCD4 3'UTR as described earlier. The STAT5- and control-shRNA lentiviral vectors were a gift of Dr. I. Dusanter-Fourt, Institut Cochin, Paris, France). For the ChIP experiments, the coding sequences of human STAT3 (NM_139276.2) and STAT5B (NM_012448.3) were PCR amplified from K562 cDNA and cloned in phase with a 3X FLAG, under the control of a MND promoter (vector #197 of the Vect'UBfacility, Bordeaux, France). Lentiviruses were prepared and titrated by the Vect'UBfacility, Bordeaux, France.

### Transfection and transduction of cell lines

For reporter analysis (miR-21 sensor plasmid, WT and mutated miR-21 promoter plasmids, WT and mutated PDCD4 3'UTR plasmid), cells were split the day before transfection at 200.000 cells/ml, then seeded in 96-well white plates (Dutscher). They were transfected with 400ng of plasmid using the Viafect reagent (Promega). For miR-21 promoter studies (pGL4.10 transfections), 20ng of the CMV-Renilla luciferase pGL4.75 plasmid (Promega) was added in order assess and take into account the transfection efficiency. The Firefly and Renilla luciferase activities luminescence were measured 24h post-transfection using the DualGlo-Luciferase Reporter Assay System (Promega). For STAT5 knock-down experiments (STAT5- or control-shRNA lentiviruses), cells were transduced at a multiplicity of infection (MOI) of 5, then sorted 48 hours later according to the expression of GFP. For miR-21 promoter activity measurements in CD34^+^ cells and AML cell lines, cells were transduced at a MOI of 5 with the miR-21 promoter lentivirus and used thereafter without selection or sorting.

For miR-21 knock down, cells were transfected with 0.1 μM of either a control or an anti-miR-21 LNA oligonucleotide (Exiqon), using the transfection reagent Lipofectamine RNAi max (Thermofisher).

### Monitoring of K562 cell growth and sensitivity to imatinib

To monitor cell growth, transfected or transduced cells were seeded in 96-well white plates (5000 cells per well). The ATP contents of the wells were assessed every day using the Cell Titer Glo reagent (Promega). For the imatinib sensitivity studies, serial dilutions of imatinib were added to the wells directly after the seeding. ATP contents were measured 48h later. The IC_50_ were calculated using GraphPad software.

### CRISPR/Cas9 inactivation of miR-21

Two oligonucleotides (CACCGGTCTGATAAGC TACCCGACA and AAACTGTCGGGTAGCTTATCA GACC) were annealed to form a double-stranded adapter that was cloned at the BbsI of the pSpCas9n(BB)-2A-Puro plasmid (a gift from Feng Zhang, Addgene # 48141). The D10 nickase Cas9 cloned into this plasmid was replaced by the wild-type (*i.e.* nuclease) version. K562 cells were transfected with the plasmid by Amaxa nucleofection, and 24 hours later puromycin (2μg/ml) was added to the culture medium. After 48 hours of selection, live cells were diluted in methylcellulose-containing medium (1000 cells per ml). After 10 days, colonies were picked up and transferred into individual wells containing liquid medium. Mutations in the miR-21 gene were detected by cloning (into the pCR2.1-TOPO^®^ plasmid (Invitrogen)) of PCR products obtained using primers CTAGCATGTACTCTGGTTTCAACAGA and CACAAAAGACTCTAAGTGCCACCA on cells lysates. Twelve distinct plasmids obtained from each cell clone were sequenced. The quantification of miR-21 expression was performed by RT-qPCR as described earlier.

### Immunoblotting

Cells were pre-treated with 10μM Na_3_VO_4_ for 30 min before collection. After two washes in PBS-Na_3_VO_4_, pellets were suspended in RIPA lysis buffer. Supernatant were collected and proteins were quantified with the Pierce BCA kit (Biorad). Samples (50μg) were denatured by incubating 5 min at 95°C in reducing sample buffer, loaded on 10% SDS-PAGE gels. After migration, proteins were transferred onto a PVDF membrane. The antibodies (PDCD4: Abcam ref. 51495; phospho-STAT5: Cell Signaling ref. 9351; STAT5: SantaCruz ref. SC-835; phospho-STAT3: Cell Signaling ref. 9131; Actin: Sigma ref. A2066) were used at a 1/1000 dilution in TBST/5% nonfat dry milk. The horseradish peroxidase-conjugated secondary antibodies (Vector Laboratories ref. PI-1000 for anti-PDCD4, phospho-STAT5, Actin antibodies; Vector Laboratories ref. PI-9500 for anti-STAT5 antibody) were used at the dilution of 1/10000 and detected using Western Lighting Plus-ECL (Perkin Elmer).

### Semi-quantitative proteomics

K562 cells (5.10^6^) were seeded at the concentration of 500.000 cells/ml and either treated with 1μM imatinib for 24h or left untreated. After centrifugation, they were washed twice with PBS and the pellets were dissolved in 100 ul of urea (8M), thiourea (2M), Chaps (2%), Hepes (50mM), pH7.5. The undissolved materials were removed by centrifugation. Proteins were precipitated in TCA 15% (v/v), washed twice with acetone and suspended in urea (7M), thiourea (2M), Chaps (4%). Supernatant protein concentration was assessed by Bradford. A hundred μg was digested with trypsin and labeled with iTRAQ 8-plex (AB SCIEX) according to the manufacturer's instructions. Briefly, samples were reduced with TCEP and alkylated with MMTS before being digested overnight with trypsin. The labeled samples were pooled and subsequently purified on SCX chromatography, desalted on a C18 cartridge and eluted peptides were submitted to the 3100 OFFGEL Fractionator (Agilent Technologies) with the OFFGEL High Res Kit pH 3-10 (Agilent Technologies) immobilized pH gradient (IPG) DryStrips. The peptides were separated according to the manufacturer's instructions. After separation, the 24 fractions were acidified with acetic acid 0.1% (v/v) and subsequently analyzed by LC-MS/MS with a nano LC system coupled ton an LTQ Orbitrap XL (ThermoFinnigan) as previously described [39]. Ten microliters of peptide digests were desalted onto a 300-μm i.d. × 5-mm C18 PepMap™ trap column (Dionex) at a flow rate of 30 μL/min and separated onto an analytical 75-μm i.d. × 15-cm C18 PepMap column (Dionex). Mobile phases were a mix of solvent A (0.1% formic acid in 5% ACN) and solvent B (0.1% formic acid in 80% ACN). Elution was performed using a 5–40% linear gradient of solvent B for 105 min. The separation flow rate was set at 200 nL/min. Data were acquired in a data-dependent mode that alternated an MS scan survey over an m/z range of 300–2000, and three MS/MS scans of top 3 ions both in CID and PQD fragmentation mode. Monocharged ions were rejected and the dynamic exclusion duration was set to 20 sec.

Data were searched by SEQUEST through Proteome Discoverer 1.3 (Thermo Fisher Scientific Inc.) against Homo sapiens Complete Proteome Set (Uniprot version 2011-01: 58009 entries). Spectra from peptides higher than 5000 Da or lower than 350 Da were rejected. The search parameters were as follows: mass accuracy of the monoisotopic peptide precursor and peptide fragments was set to 10 ppm and 0.5 Da respectively. Only b- and y-ions were considered for mass calculation. Oxidation of methionines (+16 Da) was considered as variable modification and cysteine alkylation with MMTS (+46 Da) as fixed modification. iTRAQ 8-plex labeling of lysine and peptide N-terminal were also considered as fixed modification. Two missed trypsin cleavages were allowed. Peptide validation was performed using Percolator algorithm [40] and only “high confidence” peptides were retained corresponding to a 1% False Positive Rate at peptide level. Quantitation was performed with Proteome Discoverer: protein ratios were the average of considered peptide ratio.

### Chromatin immunoprecipitation (ChIP)

K562 cells were transfected with the control plasmid, the FLAG-tagged-STAT3 plasmid or the FLAG-tagged-STAT5 plasmid. The expression of the 3X-FLAG tagged proteins waere checked by western-blot. The Pierce Agarose ChIP Kit (Thermo Fisher) was used according to the manufacturer's instructions for the steps of cross-linking, DNA digestion, immunoprecipitation (using 6 μg of a Sigma monoclonal anti-FLAG^®^ M2 antibody or normal rabbit IgG as a negative control), DNA-antibody bound complexes isolation, DNA cleanup and qPCR.

### Statistics

RT-qPCR datas and luciferase measurements were analyzed by the GraphPad software. When shown on graphs, error bars represent S.E. of the number of determination. Two-tailed Student t tests were used for statistical analysis.

## SUPPLEMENTARY MATERIALS FIGURES AND TABLES




