# Supplementary material for: A tyrosine kinase-STAT5-miR21-PDCD4 regulatory axis in chronic and acute myeloid leukemia cells
Source: Oncotarget. 2017 Jul 12;8(44):76174–88. doi: 10.18632/oncotarget.19192 (PMC5652696; doi:10.18632/oncotarget.19192)
Supplement: Supplementary file 1 [file oncotarget-08-76174-s001.pdf]

A tyrosine kinase-STAT5-miR21-PDCD4 regulatory axis in chronic and acute myeloid leukemia cells

SUPPLEMENTARY MATERIALS

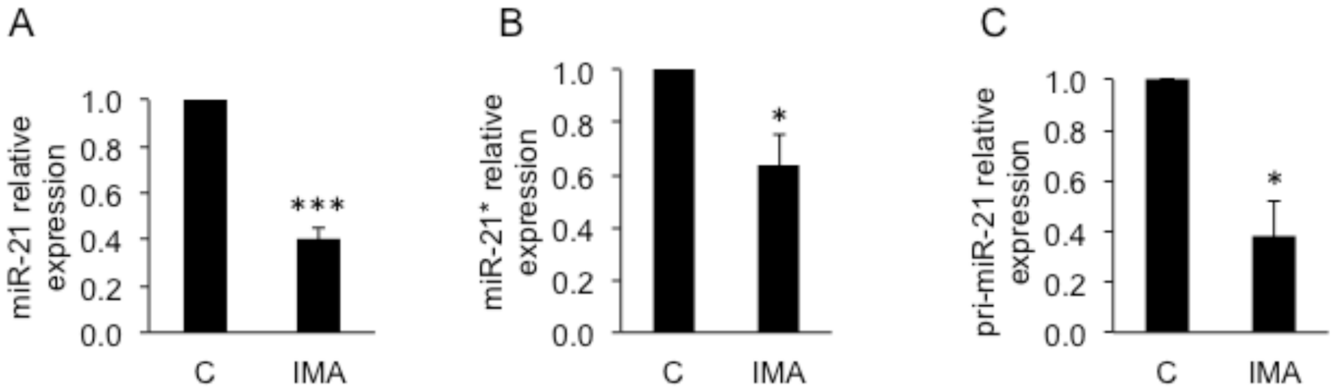

Supplementary Figure 1: Effect of imatinib treatment on LAMA-84 BCR-ABL1⁺ cells. Cells (n=3-5 independent experiments) were either not treated (C) or treated for 24 h with 1 μM imatinib (IMA) before RNA extraction, reverse transcription and quantification by qPCR of miR-21 **(A)**, miR-21* **(B)** or pri-miR-21 **(C)**. *** P<0.001, * P<0.05 (treated versus not treated cells).

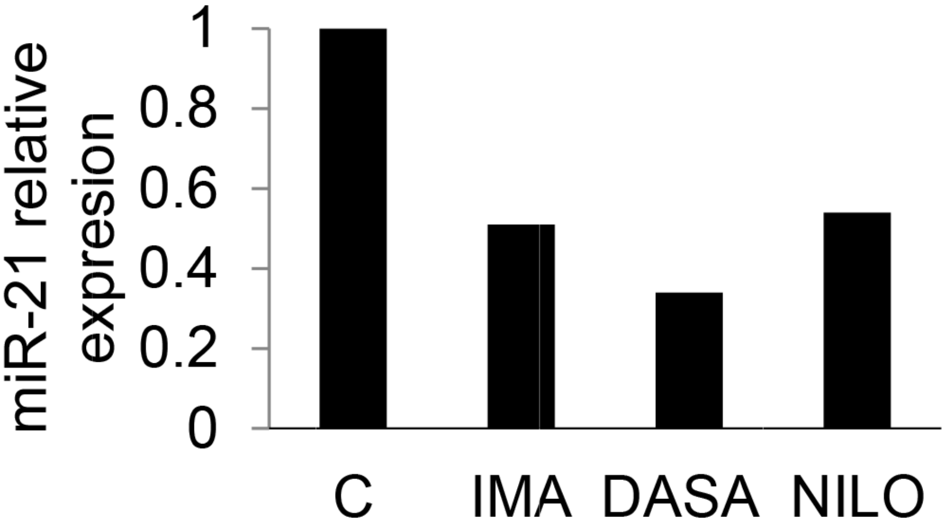

Supplementary Figure 2: Regulation of miR-21 by three tyrosine-kinase inhibitors in K562. Cells were either not treated (C) or treated for 24h with imatinib (1 µM), dasatinib (10nM) or nilotinib (20 nM). miR-21 was quantified by RT-qPCR.

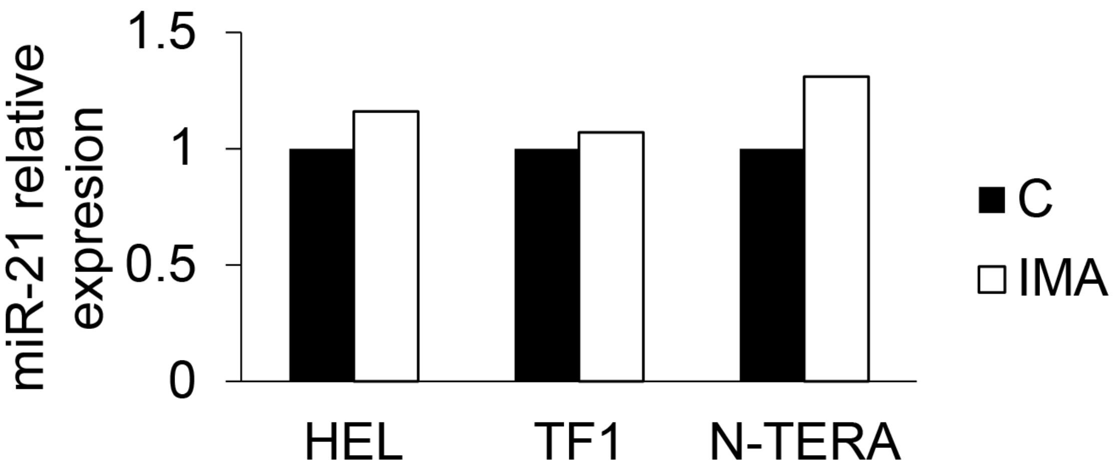

Supplementary Figure 3: Lack of effects of imatinib on BCR-ABL1-negative cell lines. The AML cell lines HEL and TF1, and the teratoma cell line N-TERA were treated with imatinib (1 µM, 24 h) before RNA extraction and miR-21 quantification by RT-qPCR.

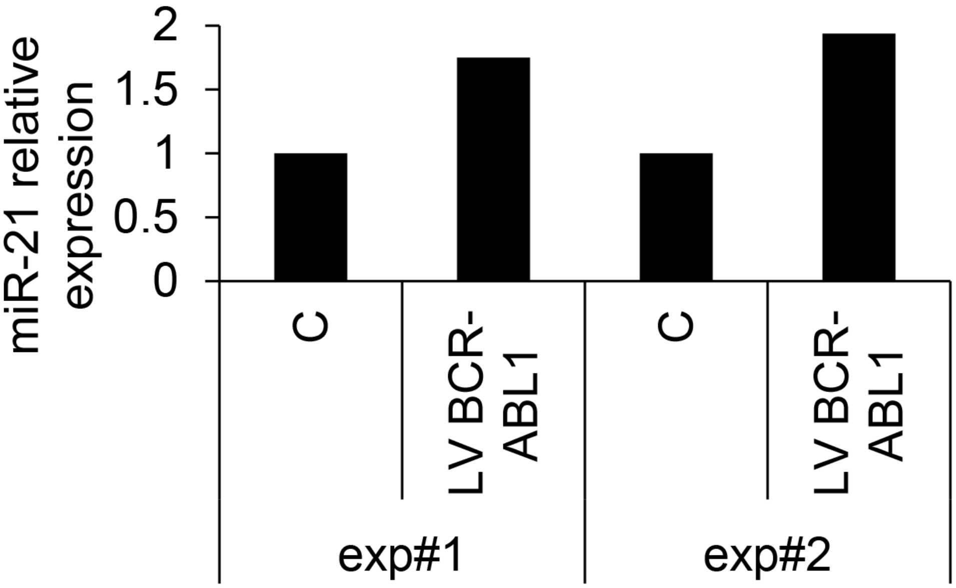

Supplementary Figure 4: Increased expression of miR-21 induced by BCR-ABL1 expression. miR-21was quantified by RT-qPCR in iPS cells produced from CD34^{+} cells that were transduced or not with a BCR-ABL1-expressing lentivirus. Exp#1 and exp#2 represent the results from two independent experiments.

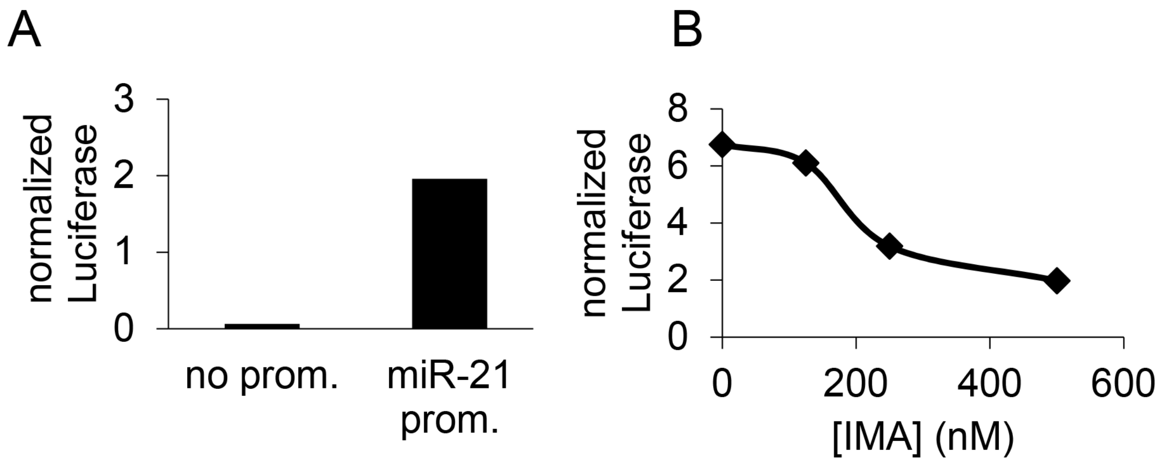

Supplementary Figure 5: Validation of the miR-21 promoter activity in K562 cells. **(A)** K562 cells were transfected with a luciferase plasmid containing either no promoter (no prom) or the 517bp fragment derived from the *miR-21* gene (miR-21 prom). The luciferase activity expressed by the transfected cells confirm the promoter activity of the cloned fragment. **(B)** On K562 cells transfected with the miR-21 promoter-luciferase plasmid, increasing concentrations of imatinib induce a dose-dependent decrease of the luciferase activity.

```
                  1                                                50
WT                CGGTTTAACA GCACTGCCTC CATGTATTCT GGGTAAGAAG GAGCTCCGAG
STATdel           ---------- ---------- ---------- ---------- ----------
STAT5del          ---------- ---------- ---------- ---------- ----------
STATmut           ---------- ---------- ---------- ---------- ----------
STAT5mut          ---------- ---------- ---------- ---------- ----------
STAT+STAT5mut     ---------- ---------- ---------- ---------- ----------

                  51                                              100
WT                TACATAAATT TATCAAAGAT CACTATCCCA ATCATCTCAG AACAAGCTGT
STATdel           ---------- ---------- ---------- ---------- ----------
STAT5del          ---------- ---------- ---------- ---------- ----------
STATmut           ---------- ---------- ---------- ---------- ----------
STAT5mut          ---------- ---------- ---------- ---------- ----------
STAT+STAT5mut     ---------- ---------- ---------- ---------- ----------

                  101                                             150
WT                TACTAATGTA CTGGAGTTTC TGTGCAAACT GTCTACCATA AACCATGAAA
STATdel           ---------- ---------- ---------- ---------- ----------
STAT5del          ---------- ---------- ---------- ---------- ----------
STATmut           ---------- ---------- ---------- ---------- ----------
STAT5mut          ---------- ---------- ---------- ---------- ----------
STAT+STAT5mut     ---------- ---------- ---------- ---------- ----------

                  151                                             200
WT                GGATTCAAAG TTCATAGTTC CTTCTTTGTT CCTTTGTTAA TCACTGACTT
STATdel           ---------- ---------- ---------- ---------- ----------
STAT5del          ---------- ---------- ---------- ---------- ----------
STATmut           ---------- ---------- ---------- ---------- ----------
STAT5mut          ---------- ---------- ---------- ---------- ----------
STAT+STAT5mut     ---------- ---------- ---------- ---------- ----------

                  201                                             250
WT                CTGACTAGTG GGAGGTGCCT CCCAAGTTTG CTAATGCATT CTTTTTGGAT
STAT3del          ---------- ---------- ---------- ---------- ----------
STAT5del          ---------- ---------- ---------- ---------- ----------
STAT3mut          ---------- ---------- ---------- ---------- ----------
STAT5mut          ---------- ---------- ---------- ---------- ----------
STAT3+STAT5mut    ---------- ---------- ---------- ---------- ----------

                  251                                             300
WT                AAGGATGACG CACAGATTGT CCTAATAAGG ACTTAGATTG AGAAAGACCG
STATdel           ---------- ---------- ---------- ---------- ----------
STAT5del          ---------- ---------- ---------- ---------- ----------
STATmut           ---------- ---------- ---------- ---------- ----------
STAT5mut          ---------- ---------- ---------- ---------- ----------
STAT+STAT5mut     ---------- ---------- ---------- ---------- ----------

                  301                                             350
WT                CCCCCTCTGA GAAGAGGGGA CAAGTCAGAG AGAGGGCGGG CAGTTTCTTT
STATdel           ---------- ---------- ---------- ---------- ----------
STAT5del          ---------- ---------- ---------- ---------- ----------
STATmut           ---------- ---------- ---------- ---------- ----------
STAT5mut          ---------- ---------- ---------- ---------- ----------
STAT+STAT5mut     ---------- ---------- ---------- ---------- ----------

                  351                                             400
WT                TTTAACTAGG GATGACACAA GCATAAGTCA TTTCCTTATT AATTGGTTCA
STATdel           ---------- ---------- ---------- ---------- ----------
STAT5del          ---------- ---------- ---------- ---------- ----------
STATmut           ---------- ---------- ---------- ---------- ----------
STAT5mut          ---------- ---------- ---------- ---------- ----------
STAT+STAT5mut     ---------- ---------- ---------- ---------- ----------

                  401                                             450
WT                AACCAGTTCT TACAGGAACT AGTGGTGATA AATGTGGGAC TTCTGAGAAG
STATdel           ---------. .......... .--------- ---------- ----------
STAT5del          ---------- ---------- ---------- --------.. ..........
STATmut           ---------- -----CT--- ---------- ---------- ----------
STAT5mut          ---------- ---------- ---------- ---------- -AG-------
STAT+STAT5mut     ---------- -----CT--- ---------- ---------- -AG-------

                  451                                             500
WT                TCATTCATTT TATTCTTTGT GCCATACCAG AGTACAGTAT CAGCTGAGCT
STATdel           ---------- ---------- ---------- ---------- ----------
STAT5del          .....----- ---------- ---------- ---------- ----------
STATmut           ---------- ---------- ---------- ---------- ----------
STAT5mut          ---------- ---------- ---------- ---------- ----------
STAT+STAT5mut     ---------- ---------- ---------- ---------- ----------

                  501            517
WT                GACCTTACTC TGAGGAC
STATdel           ---------- -------
STAT5del          ---------- -------
STATmut           ---------- -------
STAT5mut          ---------- -------
STAT+STAT5mut     ---------- -------
```

Supplementary Figure 6: Alignment of the deleted and mutated variants of the *miR-21* promoter. The STAT (position 410-418) and STAT5 (position 441-449) sites predicted by *Genomatix* are depicted in grey in the WT sequence. Deletions or mutations were produced by PCR and sequence-verified.

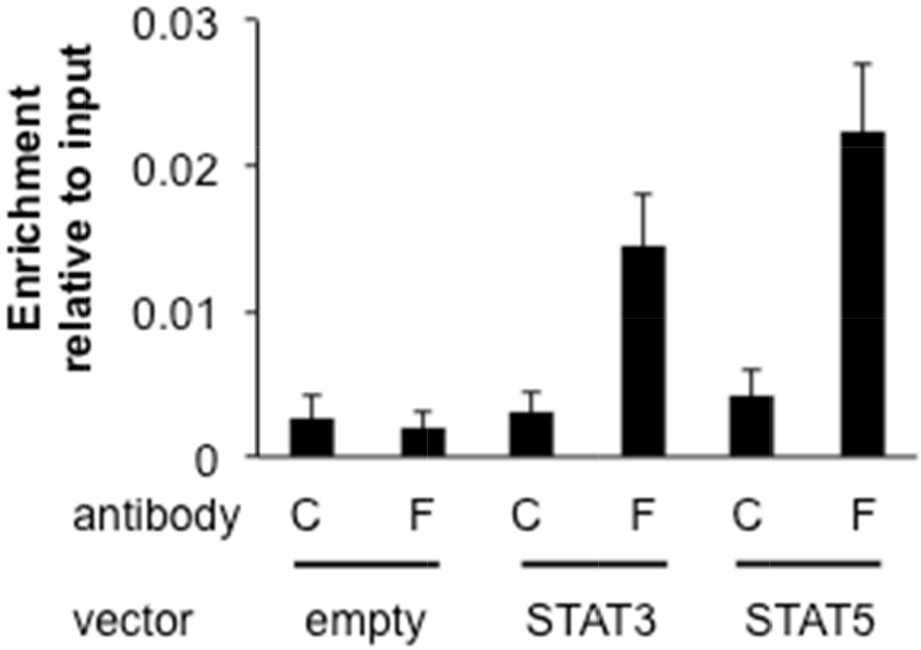

Supplementary Figure 7: Direct interaction between STAT3, STAT5 and the *miR-21* promoter. ChIP was performed with an anti-FLAG antibody (F) or with a control IgG (C) on the cell lysates of K562 cells transfected with 3×FLAG-tagged STAT3 or STAT5. The level of immunoprecipitated *miR-21* promoter was assessed by RT-qPCR. The results are presented as percentage of the input DNA.

```
                   sgRNA                   miR-21
           __________________   _______________________
WT   TCTCCATGGCTGTACCACCTTGTCGGGTAGCTTATCAGACTGATGTTGACTGTTGAATCT
5A-1 TCTCCATGGCTGTACCACCTT............ATCAGACTGATGTTGACTGTTGAATCT
5A-2 TCTCCATGGCTGTACCACCTT..............CAGACTGATGTTGACTGTTGAATCT
5A-3 TCTCCATGGCTGTA.......................GACTGATGTTGACTGTTGAATCT
```

```
                   sgRNA                   miR-21
           __________________   _______________________
 WT  TCTCCATGGCTGTACCACCTTGTCGGGTAGCTTATCAGACTGATGTTGACTGTTGAATCT
7B-1 TCTCCATGGCTGTAC.........GGGTAGCTTATCAGACTGATGTTGACTGTTGAATCT
7B-2 TCTCCATGG.................GTAGCTTATCAGACTGATGTTGACTGTTGAATCT
7B-3 TCTC....................................TGATGTCGACTGTTGAATCT
```

Supplementary Figure 8: CRISPR/Cas9-induced deletions in the *miR-21* gene. K562 were transiently transfected with the sgRNA/Cas9/Puro plasmid and selected for 48 h with puromycin. After cloning in semi-solid medium, clones were picked up and grown. A portion of the *miR-21* gene was PCR-amplified from the genomic DNA of two clones (5A and 7B). PCR products were cloned into the pCR2.1-TOPO vector, and a total of 24 plasmids were sequenced (12 for 5A and 12 for 7B). The different sequences obtained are aligned. The positions of the hybridization site of the shRNA, and of miR-21 are shown.

Supplementary Table 1: iTraq identification and relative quantification of proteins expressed in not treated and imatinib-treated K562 cells. Non treated cells (n=2 cell cultures) were labeled with iTRAQ Reagents 117 and 118. Treated cells (n=2 cell cultures) were labeled with iTRAQ Reagents 119 and 121. Only proteins that were identified by 2 peptides or more are listed. Protein ratios were the average of considered peptides ratio. Normalization between conditions was performed based on the protein ration average.

See Supplementary File 1
