# Supplementary material for: A tyrosine kinase-STAT5-miR21-PDCD4 regulatory axis in chronic and acute myeloid leukemia cells
Source: Oncotarget. 2017 Jul 12;8(44):76174–88. doi: 10.18632/oncotarget.19192 (PMC5652696; doi:10.18632/oncotarget.19192)
Supplement: Supplementary file 2 [file oncotarget-08-76174-s002.docx]

**‘**

| **Accession** | **Gene symbol** | **Coverage** | **# Proteins** | **# Unique peptides** | **# Peptides** | **# PSMs** | **118/117** | **119/117** | **119/118** | **121/117** | **121/118** | **121/119** |
| --- | --- | --- | --- | --- | --- | --- | --- | --- | --- | --- | --- | --- |
| Q5VZS7 | PDCD4 | 20.78 | 3 | 3 | 3 | 3 | 1.073 | 2.896 | 2.717 | 3.200 | 2.974 | 1.112 |
| P02008 | HBAZ | 80.28 | 2 | 9 | 10 | 192 | 1.036 | 2.011 | 2.013 | 2.159 | 2.079 | 1.090 |
| P16104 | H2AX | 37.76 | 6 | 2 | 5 | 86 | 1.316 | 1.872 | 1.420 | 1.638 | 1.240 | 0.871 |
| B9ZVT1 | B9ZVT1 | 3.18 | 6 | 2 | 2 | 3 | 1.220 | 1.807 | 1.228 | 1.997 | 1.915 | 1.100 |
| F5H5F6 | F5H5F6 | 5.38 | 5 | 2 | 2 | 4 | 1.059 | 1.687 | 1.314 | 1.513 | 1.177 | 1.043 |
| E9PDQ8 | E9PDQ8 | 5.28 | 3 | 2 | 2 | 2 | 1.012 | 1.623 | 1.602 | 1.097 | 1.081 | 0.673 |
| Q9NTZ6 | RBM12 | 2.36 | 1 | 2 | 2 | 3 | 0.963 | 1.596 | 1.628 | 0.911 | 0.944 | 0.794 |
| E7ER57 | E7ER57 | 12.31 | 5 | 3 | 5 | 11 | 1.078 | 1.587 | 1.416 | 1.243 | 1.413 | 0.893 |
| E7EUH1 | E7EUH1 | 5.84 | 5 | 3 | 3 | 4 | 1.538 | 1.515 | 1.091 | 1.193 | 0.858 | 0.807 |
| F5GZJ1 | F5GZJ1 | 1.55 | 4 | 2 | 2 | 3 | 0.992 | 1.514 | 1.523 | 1.251 | 1.257 | 0.822 |
| Q96D30 | Q96D30 | 6.75 | 9 | 2 | 2 | 4 | 1.090 | 1.492 | 1.252 | 1.063 | 1.174 | 0.826 |
| G3V1N8 | G3V1N8 | 12.50 | 3 | 2 | 2 | 2 | 1.388 | 1.488 | 1.070 | 0.997 | 0.716 | 0.667 |
| B4E0V0 | B4E0V0 | 24.10 | 4 | 2 | 2 | 2 | 0.814 | 1.459 | 1.662 |  |  |  |
| P30613 | KPYR | 12.20 | 2 | 4 | 6 | 12 | 0.918 | 1.455 | 1.598 | 1.507 | 1.651 | 1.006 |
| F8WAN8 | F8WAN8 | 7.38 | 2 | 3 | 3 | 3 | 1.194 | 1.442 | 1.221 | 0.972 | 1.069 | 0.757 |
| O75396 | SC22B | 19.07 | 1 | 3 | 3 | 5 | 1.339 | 1.432 | 0.979 | 1.333 | 0.892 | 0.884 |
| P02100 | HBE | 63.27 | 7 | 6 | 8 | 84 | 1.088 | 1.415 | 1.310 | 1.345 | 1.270 | 1.002 |
| A6NKB8 | A6NKB8 | 3.27 | 2 | 2 | 2 | 4 | 1.080 | 1.412 | 1.206 | 1.161 | 1.045 | 0.819 |
| Q16836 | HCDH | 16.24 | 2 | 4 | 4 | 19 | 0.919 | 1.406 | 1.457 | 1.049 | 1.267 | 0.855 |
| P42765 | THIM | 8.06 | 1 | 2 | 2 | 2 | 1.010 | 1.401 | 1.385 | 1.692 | 1.581 | 1.339 |
| Q8TEM1 | PO210 | 4.50 | 1 | 6 | 6 | 6 | 1.006 | 1.391 | 0.978 | 1.129 | 0.880 | 0.913 |
| P06132 | DCUP | 8.45 | 1 | 3 | 3 | 9 | 1.192 | 1.387 | 1.158 | 1.245 | 1.047 | 0.959 |
| P11387 | TOP1 | 4.05 | 1 | 3 | 3 | 10 | 1.082 | 1.377 | 0.736 | 1.327 | 1.038 | 1.114 |
| P47985 | UCRI | 10.58 | 2 | 2 | 2 | 2 | 1.176 | 1.374 | 1.166 | 1.213 | 1.028 | 0.879 |
| Q5T8U5 | Q5T8U5 | 12.90 | 6 | 2 | 2 | 6 | 1.121 | 1.365 | 0.984 | 1.181 | 0.950 | 0.980 |
| C9JIM8 | C9JIM8 | 3.60 | 3 | 2 | 2 | 11 | 1.093 | 1.364 | 1.252 | 1.334 | 1.244 | 0.976 |
| B4DZN0 | B4DZN0 | 11.39 | 4 | 2 | 2 | 3 | 1.328 | 1.347 | 0.714 | 1.650 | 0.873 | 1.219 |
| P02549 | SPTA1 | 1.86 | 1 | 3 | 4 | 4 | 0.864 | 1.346 | 1.104 | 0.908 | 0.996 | 0.899 |
| Q07021 | C1QBP | 9.57 | 1 | 2 | 2 | 3 | 1.154 | 1.339 | 1.072 | 1.144 | 1.062 | 0.988 |
| P24928 | RPB1 | 2.28 | 2 | 3 | 3 | 3 | 1.341 | 1.337 | 0.973 | 1.241 | 0.903 | 0.924 |
| E9PCI9 | E9PCI9 | 7.65 | 2 | 2 | 2 | 3 | 1.039 | 1.336 | 1.205 | 1.142 | 1.080 | 0.910 |
| E9PCB6 | E9PCB6 | 4.33 | 3 | 2 | 2 | 7 | 1.372 | 1.329 | 0.996 | 1.517 | 1.145 | 1.155 |
| E9PC79 | E9PC79 | 5.17 | 4 | 2 | 2 | 2 | 1.097 | 1.322 | 1.203 | 1.210 | 1.100 | 0.911 |
| E7EMK1 | E7EMK1 | 12.73 | 3 | 4 | 4 | 6 | 0.931 | 1.321 | 1.413 | 1.240 | 1.340 | 0.921 |
| Q13011 | ECH1 | 20.43 | 1 | 5 | 5 | 9 | 1.062 | 1.320 | 1.149 | 1.420 | 1.167 | 1.035 |
| F2Z2V0 | F2Z2V0 | 10.32 | 12 | 4 | 4 | 5 | 0.997 | 1.317 | 1.185 | 1.052 | 1.012 | 0.786 |
| F5H3P3 | F5H3P3 | 8.81 | 2 | 2 | 2 | 4 | 1.293 | 1.316 | 1.019 | 1.063 | 0.970 | 0.949 |
| F5GXD5 | F5GXD5 | 10.39 | 9 | 5 | 5 | 5 | 0.924 | 1.311 | 1.175 | 1.208 | 1.076 | 0.932 |
| Q92973 | TNPO1 | 10.91 | 6 | 9 | 9 | 13 | 1.029 | 1.310 | 1.077 | 1.222 | 1.036 | 0.936 |
| F8W8R3 | F8W8R3 | 6.81 | 6 | 2 | 2 | 2 | 1.114 | 1.309 | 1.173 | 1.325 | 1.186 | 1.007 |
| F5H1F9 | F5H1F9 | 7.64 | 3 | 2 | 2 | 5 | 1.024 | 1.305 | 1.233 | 0.988 | 0.978 | 0.690 |
| P13645 | K1C10 | 3.60 | 4 | 2 | 2 | 2 | 1.013 | 1.300 | 1.281 | 1.283 | 1.262 | 0.982 |
| Q9Y2X3 | NOP58 | 10.21 | 1 | 3 | 3 | 16 | 1.260 | 1.299 | 1.095 | 1.370 | 1.145 | 1.057 |
| E7EW33 | E7EW33 | 2.18 | 9 | 2 | 2 | 2 | 0.989 | 1.292 | 1.305 | 1.057 | 1.066 | 0.814 |
| P46977 | STT3A | 5.96 | 5 | 5 | 5 | 6 | 1.261 | 1.288 | 0.947 | 1.456 | 1.000 | 0.992 |
| P21912 | DHSB | 10.71 | 1 | 3 | 3 | 5 | 1.066 | 1.285 | 1.152 | 1.227 | 1.163 | 0.976 |
| F8WJN3 | F8WJN3 | 10.88 | 4 | 3 | 3 | 3 | 0.956 | 1.283 | 1.340 | 0.990 | 1.033 | 0.768 |
| P49458 | SRP09 | 18.60 | 2 | 2 | 2 | 10 | 1.034 | 1.283 | 1.152 | 1.598 | 1.457 | 1.276 |
| Q9NRW3 | ABC3C | 12.63 | 4 | 2 | 2 | 8 | 0.957 | 1.278 | 1.282 | 1.234 | 1.152 | 0.907 |
| E9PEI9 | E9PEI9 | 4.58 | 3 | 3 | 3 | 3 | 0.960 | 1.277 | 1.138 | 1.280 | 0.998 | 0.903 |
| B4DQI6 | B4DQI6 | 11.11 | 3 | 2 | 2 | 6 | 1.067 | 1.275 | 1.167 | 1.260 | 1.180 | 1.015 |
| Q9BPX3 | CND3 | 6.21 | 2 | 6 | 6 | 9 | 1.029 | 1.274 | 1.205 | 1.078 | 1.059 | 0.960 |
| C9J4M6 | C9J4M6 | 4.09 | 3 | 4 | 4 | 8 | 1.172 | 1.271 | 1.060 | 1.178 | 1.015 | 0.925 |
| Q86UX7 | URP2 | 8.85 | 2 | 4 | 4 | 4 | 1.072 | 1.268 | 1.310 | 1.301 | 1.168 | 1.021 |
| B3KUK2 | B3KUK2 | 12.50 | 7 | 2 | 2 | 5 | 1.038 | 1.263 | 1.217 | 1.237 | 1.191 | 0.975 |
| P49321 | NASP | 13.58 | 8 | 8 | 9 | 18 | 1.043 | 1.262 | 1.136 | 1.361 | 1.248 | 0.968 |
| P04181 | OAT | 37.13 | 2 | 14 | 14 | 46 | 1.039 | 1.260 | 1.236 | 1.227 | 1.168 | 0.970 |
| Q15427 | SF3B4 | 12.74 | 2 | 3 | 3 | 4 | 1.152 | 1.258 | 1.091 | 0.992 | 0.961 | 0.784 |
| B4DJC3 | B4DJC3 | 9.50 | 5 | 2 | 2 | 5 | 1.350 | 1.258 | 0.797 | 1.291 | 0.953 | 1.202 |
| F5H315 | F5H315 | 2.93 | 2 | 2 | 2 | 2 | 0.970 | 1.257 | 1.294 | 0.900 | 0.925 | 0.713 |
| B4DFM5 | B4DFM5 | 20.52 | 6 | 5 | 5 | 10 | 0.914 | 1.255 | 1.573 | 1.002 | 1.067 | 0.638 |
| P52907 | CAZA1 | 18.53 | 1 | 3 | 3 | 4 | 1.064 | 1.252 | 1.150 | 1.062 | 1.083 | 0.939 |
| B4DM74 | B4DM74 | 11.04 | 2 | 2 | 2 | 13 | 2.089 | 1.252 | 0.629 | 1.981 | 0.916 | 1.530 |
| Q16864 | VATF | 24.37 | 2 | 2 | 2 | 7 | 1.134 | 1.251 | 1.104 | 1.300 | 1.023 | 1.034 |
| Q9BQA1 | MEP50 | 6.14 | 2 | 2 | 2 | 4 | 1.143 | 1.248 | 1.130 | 1.174 | 1.055 | 0.954 |
| Q5TGE0 | Q5TGE0 | 16.19 | 7 | 2 | 2 | 3 | 0.997 | 1.248 | 1.249 | 1.242 | 1.241 | 0.991 |
| D6RDM7 | D6RDM7 | 21.95 | 4 | 2 | 2 | 2 | 1.192 | 1.242 | 1.040 | 1.415 | 1.183 | 1.134 |
| D6RFH4 | D6RFH4 | 16.03 | 2 | 2 | 2 | 3 | 1.118 | 1.241 | 1.132 | 1.072 | 0.956 | 0.892 |
| Q9H0S4 | DDX47 | 4.18 | 2 | 2 | 2 | 2 | 1.165 | 1.239 | 1.062 | 1.099 | 0.940 | 0.883 |
| B7ZM62 | B7ZM62 | 2.75 | 7 | 2 | 2 | 2 | 1.029 | 1.237 | 1.200 | 0.881 | 0.853 | 0.709 |
| Q9Y570 | PPME1 | 8.03 | 1 | 3 | 3 | 4 | 1.056 | 1.236 | 1.169 | 1.244 | 1.087 | 0.974 |
| P22033 | MUTA | 3.73 | 1 | 2 | 2 | 2 | 1.362 | 1.236 | 0.906 | 1.119 | 0.819 | 0.901 |
| Q9H4M9 | EHD1 | 10.67 | 13 | 3 | 5 | 12 | 1.016 | 1.234 | 1.286 | 1.051 | 0.933 | 0.864 |
| A6NHR9 | SMHD1 | 2.19 | 1 | 4 | 4 | 4 | 0.998 | 1.233 | 1.101 | 1.315 | 1.172 | 1.061 |
| P17858 | K6PL | 13.59 | 4 | 7 | 8 | 14 | 1.048 | 1.233 | 1.205 | 0.990 | 0.983 | 0.869 |
| P00568 | KAD1 | 18.56 | 2 | 3 | 3 | 21 | 0.963 | 1.232 | 1.330 | 1.320 | 1.422 | 0.992 |
| Q1KMD3 | HNRL2 | 6.69 | 1 | 3 | 3 | 3 | 0.999 | 1.232 | 1.156 | 1.219 | 1.210 | 1.044 |
| P42224 | STAT1 | 5.07 | 5 | 3 | 3 | 3 | 1.046 | 1.225 | 1.132 | 1.321 | 1.219 | 1.073 |
| F5H2E2 | F5H2E2 | 3.37 | 17 | 3 | 3 | 4 | 1.176 | 1.224 | 0.950 | 1.344 | 1.021 | 1.005 |
| Q92905 | CSN5 | 5.99 | 2 | 2 | 2 | 3 | 1.097 | 1.224 | 1.114 | 1.109 | 1.008 | 0.902 |
| Q02978 | M2OM | 9.87 | 2 | 3 | 3 | 6 | 1.047 | 1.223 | 1.125 | 1.204 | 1.059 | 0.953 |
| P16435 | NCPR | 6.20 | 7 | 3 | 3 | 5 | 0.828 | 1.223 | 1.272 | 1.030 | 1.157 | 0.842 |
| Q5T2B5 | Q5T2B5 | 4.39 | 5 | 3 | 3 | 4 | 0.848 | 1.221 | 1.536 | 0.961 | 1.207 | 0.794 |
| F5H6P7 | F5H6P7 | 29.41 | 7 | 2 | 2 | 5 | 1.040 | 1.218 | 1.255 | 1.082 | 1.007 | 1.014 |
| G3V126 | G3V126 | 14.67 | 3 | 4 | 4 | 4 | 0.999 | 1.217 | 1.132 | 0.853 | 0.852 | 0.824 |
| Q96I25 | SPF45 | 4.49 | 5 | 2 | 2 | 3 | 1.182 | 1.217 | 1.094 | 1.090 | 0.973 | 0.891 |
| P17612 | KAPCA | 13.39 | 15 | 3 | 3 | 7 | 1.035 | 1.215 | 1.084 | 0.984 | 0.865 | 0.858 |
| P36551 | HEM6 | 7.05 | 1 | 3 | 3 | 4 | 1.074 | 1.215 | 1.117 | 1.052 | 0.977 | 0.861 |
| B4DU58 | B4DU58 | 18.04 | 5 | 5 | 5 | 6 | 1.010 | 1.215 | 1.091 | 1.126 | 0.954 | 0.959 |
| Q6NUK1 | SCMC1 | 5.03 | 1 | 2 | 2 | 4 | 1.100 | 1.214 | 1.223 | 1.079 | 0.917 | 0.806 |
| Q9H0P0 | 5NT3 | 8.63 | 1 | 2 | 2 | 2 | 1.137 | 1.213 | 1.065 | 1.246 | 1.092 | 1.022 |
| Q96T37 | RBM15 | 2.66 | 1 | 2 | 2 | 9 | 0.906 | 1.212 | 1.235 | 1.036 | 1.179 | 0.982 |
| B3KST5 | B3KST5 | 8.24 | 5 | 2 | 2 | 2 | 1.168 | 1.210 | 1.034 | 1.345 | 1.148 | 1.106 |
| Q15691 | MARE1 | 8.21 | 1 | 2 | 2 | 8 | 1.153 | 1.206 | 1.033 | 1.210 | 1.136 | 0.924 |
| P48147 | PPCE | 4.65 | 1 | 4 | 4 | 6 | 1.256 | 1.204 | 0.957 | 1.115 | 0.885 | 0.922 |
| B4DG89 | B4DG89 | 6.36 | 2 | 2 | 2 | 2 | 0.996 | 1.200 | 1.105 | 1.085 | 0.998 | 0.900 |
| B4E3S0 | B4E3S0 | 13.82 | 10 | 5 | 5 | 17 | 1.041 | 1.198 | 1.148 | 1.150 | 1.060 | 0.927 |
| P51159 | RB27A | 10.86 | 1 | 2 | 2 | 2 | 0.801 | 1.197 | 1.492 | 0.979 | 1.218 | 0.814 |
| B4DM17 | B4DM17 | 3.65 | 7 | 2 | 2 | 3 | 1.040 | 1.196 | 1.488 | 1.290 | 1.473 | 1.073 |
| P49721 | PSB2 | 18.91 | 1 | 3 | 3 | 22 | 1.064 | 1.193 | 1.127 | 1.131 | 1.025 | 0.934 |
| Q9NUQ9 | FA49B | 8.02 | 12 | 2 | 2 | 6 | 1.131 | 1.193 | 1.089 | 0.955 | 0.964 | 0.813 |
| Q13243 | SRSF5 | 10.29 | 5 | 2 | 3 | 5 | 1.021 | 1.185 | 0.984 | 1.106 | 0.927 | 0.999 |
| Q5T6W5 | Q5T6W5 | 46.03 | 2 | 3 | 19 | 149 | 1.214 | 1.184 | 1.031 | 1.145 | 0.949 | 0.938 |
| G3V0F2 | G3V0F2 | 6.56 | 6 | 2 | 2 | 3 | 1.095 | 1.184 | 1.015 | 1.183 | 1.011 | 0.993 |
| Q92835 | SHIP1 | 2.61 | 2 | 2 | 2 | 2 | 0.960 | 1.184 | 1.231 | 0.997 | 1.035 | 0.838 |
| Q9UJX3 | APC7 | 5.84 | 3 | 3 | 3 | 7 | 0.886 | 1.178 | 1.267 | 1.062 | 1.304 | 0.997 |
| Q14CF1 | Q14CF1 | 3.25 | 3 | 4 | 5 | 13 | 1.059 | 1.178 | 1.111 | 1.143 | 1.055 | 0.884 |
| Q9BUB1 | Q9BUB1 | 16.49 | 3 | 3 | 4 | 8 | 1.034 | 1.176 | 1.157 | 1.206 | 1.176 | 0.960 |
| Q8WYJ6 | sept-01 | 13.62 | 1 | 4 | 4 | 5 | 0.765 | 1.172 | 1.449 | 0.837 | 1.102 | 0.710 |
| Q9UJZ1 | STML2 | 18.54 | 2 | 5 | 5 | 12 | 1.003 | 1.170 | 1.047 | 1.119 | 1.050 | 0.909 |
| Q13573 | SNW1 | 8.77 | 5 | 2 | 2 | 2 | 1.048 | 1.169 | 1.113 | 1.487 | 1.414 | 1.266 |
| P22059 | OSBP1 | 3.47 | 2 | 3 | 3 | 4 | 1.052 | 1.169 | 1.033 | 1.236 | 1.018 | 1.028 |
| Q9BVP2 | GNL3 | 4.74 | 1 | 2 | 2 | 5 | 1.182 | 1.168 | 1.059 | 1.057 | 0.859 | 0.808 |
| F5H6R2 | F5H6R2 | 2.70 | 6 | 4 | 4 | 4 | 1.170 | 1.166 | 0.995 | 1.360 | 1.153 | 1.032 |
| P55060 | XPO2 | 6.80 | 7 | 7 | 7 | 15 | 1.110 | 1.165 | 1.069 | 1.052 | 0.961 | 0.913 |
| F5H6J5 | F5H6J5 | 11.89 | 3 | 2 | 2 | 2 | 1.073 | 1.163 | 1.058 | 1.087 | 0.987 | 0.929 |
| O43865 | SAHH2 | 4.34 | 1 | 2 | 2 | 3 | 0.845 | 1.163 | 1.373 | 1.101 | 1.298 | 0.942 |
| Q15428 | SF3A2 | 7.11 | 1 | 3 | 3 | 9 | 1.031 | 1.163 | 1.298 | 1.186 | 1.202 | 0.855 |
| P51784 | UBP11 | 2.49 | 1 | 2 | 2 | 2 | 1.006 | 1.161 | 1.152 | 0.831 | 0.823 | 0.712 |
| P30043 | BLVRB | 35.44 | 1 | 5 | 5 | 35 | 0.977 | 1.160 | 1.191 | 1.185 | 1.215 | 0.986 |
| G3V1R9 | G3V1R9 | 7.60 | 2 | 3 | 3 | 28 | 1.123 | 1.159 | 1.096 | 1.119 | 1.012 | 0.973 |
| P61019 | RAB2A | 30.66 | 6 | 5 | 5 | 5 | 0.947 | 1.158 | 1.127 | 1.160 | 1.075 | 0.942 |
| P53985 | MOT1 | 5.00 | 4 | 2 | 2 | 3 | 1.160 | 1.157 | 1.021 | 1.081 | 0.929 | 0.930 |
| F5H1R5 | F5H1R5 | 17.53 | 2 | 4 | 5 | 10 | 1.048 | 1.156 | 1.116 | 1.155 | 1.120 | 1.071 |
| Q8IYJ9 | Q8IYJ9 | 16.67 | 2 | 2 | 2 | 4 | 0.886 | 1.155 | 1.344 | 0.863 | 1.004 | 0.744 |
| Q9BZE4 | NOG1 | 2.68 | 3 | 2 | 2 | 2 | 1.255 | 1.152 | 0.916 | 1.267 | 1.007 | 1.095 |
| E9PH64 | E9PH64 | 14.88 | 4 | 2 | 2 | 3 | 0.978 | 1.152 | 1.177 | 1.011 | 1.118 | 0.873 |
| A6NG06 | A6NG06 | 38.82 | 3 | 6 | 6 | 24 | 1.076 | 1.151 | 1.122 | 1.143 | 1.130 | 0.969 |
| F5GZ76 | F5GZ76 | 5.27 | 4 | 4 | 4 | 4 | 1.099 | 1.151 | 1.089 | 0.961 | 0.938 | 0.884 |
| F5H2G1 | F5H2G1 | 4.61 | 8 | 6 | 6 | 13 | 1.038 | 1.151 | 1.029 | 1.043 | 0.939 | 0.895 |
| P61158 | ARP3 | 21.77 | 7 | 8 | 8 | 30 | 1.080 | 1.150 | 1.091 | 1.125 | 1.004 | 0.932 |
| P20618 | PSB1 | 37.34 | 1 | 8 | 8 | 20 | 1.034 | 1.150 | 1.179 | 1.058 | 1.045 | 0.936 |
| B4DIH5 | B4DIH5 | 13.46 | 2 | 5 | 5 | 10 | 1.033 | 1.149 | 1.082 | 0.942 | 0.983 | 0.897 |
| F5GYF5 | F5GYF5 | 7.55 | 2 | 4 | 4 | 29 | 0.962 | 1.148 | 1.209 | 1.025 | 1.077 | 0.886 |
| P11766 | ADHX | 4.55 | 1 | 2 | 2 | 3 | 1.016 | 1.147 | 1.122 | 1.223 | 1.124 | 1.062 |
| P62280 | RS11 | 20.25 | 1 | 4 | 4 | 16 | 1.290 | 1.147 | 0.901 | 1.320 | 1.036 | 1.101 |
| Q8IXW7 | Q8IXW7 | 9.09 | 4 | 2 | 2 | 2 | 0.960 | 1.146 | 1.192 | 0.889 | 0.924 | 0.772 |
| P56385 | ATP5I | 34.78 | 1 | 2 | 2 | 4 | 0.989 | 1.145 | 1.100 | 1.279 | 1.060 | 0.961 |
| Q7L9L4 | MOL1A | 10.65 | 4 | 2 | 2 | 2 | 1.052 | 1.144 | 1.086 | 1.234 | 1.170 | 1.074 |
| Q8WX92 | NELFB | 3.97 | 1 | 2 | 2 | 3 | 0.968 | 1.142 | 1.129 | 0.893 | 0.945 | 0.834 |
| Q13085 | ACACA | 3.07 | 5 | 5 | 5 | 17 | 1.063 | 1.141 | 1.025 | 0.975 | 0.939 | 0.945 |
| Q86VP6 | CAND1 | 14.55 | 3 | 15 | 15 | 28 | 1.069 | 1.141 | 1.047 | 1.085 | 0.957 | 0.916 |
| B4DNR3 | B4DNR3 | 12.43 | 4 | 2 | 2 | 6 | 1.225 | 1.139 | 0.750 | 1.325 | 0.861 | 1.188 |
| G3V0E8 | G3V0E8 | 18.87 | 33 | 2 | 5 | 17 | 0.956 | 1.139 | 1.065 | 1.061 | 1.068 | 1.008 |
| O15372 | EIF3H | 13.64 | 3 | 4 | 4 | 6 | 1.006 | 1.137 | 1.082 | 1.122 | 1.046 | 0.979 |
| O00425 | IF2B3 | 12.78 | 1 | 2 | 6 | 17 | 0.962 | 1.137 | 1.180 | 1.034 | 1.071 | 0.905 |
| E9PBR7 | E9PBR7 | 3.68 | 2 | 2 | 2 | 2 | 1.186 | 1.137 | 0.956 | 1.251 | 1.052 | 1.096 |
| B4E2W0 | B4E2W0 | 21.68 | 7 | 9 | 9 | 24 | 1.102 | 1.135 | 1.022 | 1.160 | 1.085 | 1.014 |
| O43598 | RCL | 19.54 | 1 | 2 | 2 | 5 | 0.946 | 1.135 | 1.315 | 0.982 | 1.022 | 0.839 |
| P61106 | RAB14 | 12.09 | 2 | 2 | 3 | 4 | 1.042 | 1.134 | 0.948 | 1.217 | 1.060 | 1.294 |
| Q8WUM4 | PDC6I | 17.40 | 3 | 14 | 14 | 20 | 1.124 | 1.134 | 1.051 | 1.109 | 0.982 | 0.901 |
| B1A8Z4 | B1A8Z4 | 1.43 | 8 | 2 | 2 | 2 | 1.155 | 1.133 | 0.980 | 1.028 | 0.887 | 0.902 |
| P14678 | RSMB | 15.83 | 5 | 5 | 5 | 37 | 1.138 | 1.132 | 0.981 | 1.119 | 0.981 | 0.997 |
| B4DM63 | B4DM63 | 18.56 | 4 | 3 | 3 | 6 | 1.012 | 1.132 | 1.219 | 0.885 | 0.964 | 1.085 |
| C9JFC3 | C9JFC3 | 3.78 | 5 | 2 | 2 | 8 | 1.088 | 1.131 | 1.135 | 1.109 | 1.009 | 0.834 |
| P55265 | DSRAD | 3.26 | 2 | 3 | 3 | 4 | 1.103 | 1.131 | 1.034 | 1.103 | 0.944 | 0.926 |
| P46108 | CRK | 10.53 | 1 | 2 | 2 | 3 | 0.940 | 1.130 | 1.143 | 0.970 | 0.943 | 1.009 |
| D6RCN7 | D6RCN7 | 16.78 | 4 | 2 | 2 | 2 | 1.046 | 1.130 | 1.079 | 1.174 | 1.119 | 1.034 |
| G3V1P4 | G3V1P4 | 15.46 | 5 | 3 | 3 | 7 | 0.959 | 1.129 | 1.100 | 1.235 | 1.464 | 1.048 |
| E7ENC1 | E7ENC1 | 12.21 | 14 | 2 | 2 | 2 | 0.884 | 1.129 | 1.274 | 1.169 | 1.318 | 1.031 |
| P25788 | PSA3 | 12.94 | 2 | 3 | 3 | 15 | 1.101 | 1.125 | 1.016 | 1.123 | 1.023 | 1.001 |
| P41240 | CSK | 6.89 | 2 | 3 | 3 | 5 | 0.991 | 1.124 | 1.154 | 0.853 | 0.983 | 0.842 |
| P33176 | KINH | 10.28 | 4 | 7 | 7 | 12 | 0.993 | 1.124 | 1.026 | 0.964 | 1.032 | 0.907 |
| F8WD96 | F8WD96 | 11.23 | 3 | 2 | 2 | 5 | 1.095 | 1.123 | 1.114 | 1.259 | 1.015 | 0.956 |
| E7ERV9 | E7ERV9 | 9.51 | 4 | 2 | 2 | 2 | 1.121 | 1.122 | 0.999 | 1.372 | 1.219 | 1.216 |
| Q12905 | ILF2 | 33.33 | 1 | 9 | 9 | 27 | 1.064 | 1.122 | 1.040 | 1.109 | 1.064 | 0.993 |
| A6NJA2 | A6NJA2 | 17.86 | 3 | 6 | 6 | 20 | 1.160 | 1.121 | 0.992 | 1.058 | 1.028 | 1.086 |
| E7EUY5 | E7EUY5 | 28.15 | 3 | 4 | 4 | 34 | 1.026 | 1.120 | 0.963 | 0.979 | 0.961 | 0.900 |
| B4DFL2 | B4DFL2 | 18.25 | 3 | 6 | 6 | 26 | 1.037 | 1.120 | 1.124 | 1.164 | 1.122 | 1.017 |
| Q9HB71 | CYBP | 16.67 | 4 | 5 | 5 | 11 | 1.006 | 1.120 | 1.076 | 1.094 | 1.078 | 1.047 |
| E7EWC2 | E7EWC2 | 1.36 | 4 | 2 | 2 | 3 | 1.084 | 1.120 | 1.180 | 1.066 | 1.179 | 0.996 |
| B4DFA2 | B4DFA2 | 11.65 | 3 | 6 | 7 | 10 | 0.961 | 1.120 | 1.053 | 1.065 | 1.113 | 0.968 |
| C9JTI6 | C9JTI6 | 9.04 | 2 | 2 | 2 | 15 | 1.045 | 1.120 | 1.056 | 1.031 | 0.990 | 0.942 |
| P49750 | YLPM1 | 1.44 | 2 | 2 | 2 | 3 | 0.825 | 1.119 | 1.285 | 0.980 | 1.204 | 0.926 |
| O60341 | KDM1A | 7.28 | 2 | 5 | 5 | 8 | 0.991 | 1.119 | 1.057 | 1.078 | 1.079 | 0.936 |
| E9PEX0 | E9PEX0 | 4.40 | 4 | 3 | 3 | 5 | 1.125 | 1.118 | 0.950 | 1.228 | 1.033 | 1.028 |
| C9J3L8 | C9J3L8 | 12.83 | 6 | 3 | 3 | 15 | 1.021 | 1.117 | 1.099 | 0.973 | 0.942 | 0.972 |
| C9IZN7 | C9IZN7 | 8.13 | 3 | 2 | 2 | 3 | 0.956 | 1.116 | 1.088 | 0.916 | 0.955 | 0.817 |
| Q96A35 | RM24 | 10.65 | 1 | 2 | 2 | 2 | 1.099 | 1.115 | 1.013 | 1.236 | 1.121 | 1.104 |
| D6RG15 | D6RG15 | 10.24 | 4 | 2 | 2 | 3 | 1.168 | 1.115 | 0.953 | 1.120 | 0.956 | 1.000 |
| E9PDK5 | E9PDK5 | 13.83 | 4 | 4 | 4 | 5 | 0.973 | 1.115 | 1.326 | 1.019 | 1.044 | 0.917 |
| F8W1I6 | F8W1I6 | 6.98 | 9 | 2 | 2 | 2 | 0.928 | 1.114 | 1.198 | 1.232 | 1.323 | 1.100 |
| P20645 | MPRD | 14.08 | 4 | 2 | 2 | 2 | 0.739 | 1.113 | 1.346 | 0.967 | 1.305 | 0.870 |
| Q07960 | RHG01 | 7.97 | 3 | 3 | 3 | 3 | 1.198 | 1.113 | 0.937 | 1.171 | 0.950 | 1.078 |
| E7EVX7 | E7EVX7 | 2.83 | 8 | 4 | 4 | 6 | 1.086 | 1.112 | 0.892 | 1.308 | 1.201 | 1.135 |
| P25789 | PSA4 | 6.51 | 2 | 2 | 2 | 11 | 1.027 | 1.112 | 1.162 | 1.110 | 1.120 | 0.958 |
| O95336 | 6PGL | 12.02 | 1 | 2 | 2 | 7 | 0.741 | 1.111 | 1.285 | 1.072 | 1.289 | 0.960 |
| P24752 | THIL | 18.97 | 3 | 6 | 6 | 16 | 0.981 | 1.111 | 1.084 | 0.999 | 1.016 | 0.969 |
| Q9UNM6 | PSD13 | 20.48 | 5 | 7 | 7 | 9 | 1.007 | 1.111 | 1.176 | 0.961 | 0.999 | 0.992 |
| C0H5W9 | C0H5W9 | 7.24 | 4 | 2 | 2 | 2 | 1.010 | 1.111 | 1.097 | 1.160 | 1.144 | 1.039 |
| O75368 | SH3L1 | 40.35 | 1 | 4 | 4 | 5 | 0.971 | 1.110 | 1.053 | 1.018 | 0.964 | 0.966 |
| P50213 | IDH3A | 13.93 | 2 | 4 | 4 | 9 | 1.078 | 1.110 | 1.030 | 0.956 | 0.884 | 0.786 |
| P62841 | RS15 | 28.28 | 1 | 2 | 2 | 21 | 1.053 | 1.110 | 1.253 | 1.274 | 1.212 | 0.980 |
| Q9P0J7 | KCMF1 | 10.50 | 2 | 3 | 3 | 9 | 1.285 | 1.110 | 1.033 | 1.160 | 1.071 | 1.034 |
| Q8N163 | K1967 | 8.13 | 4 | 6 | 6 | 7 | 1.040 | 1.110 | 1.007 | 1.023 | 0.962 | 0.960 |
| Q92499 | DDX1 | 9.73 | 3 | 4 | 4 | 12 | 0.992 | 1.108 | 1.287 | 0.872 | 0.938 | 0.815 |
| P12277 | KCRB | 35.43 | 4 | 10 | 10 | 67 | 0.963 | 1.108 | 1.151 | 1.075 | 1.087 | 0.938 |
| E7ENF9 | E7ENF9 | 11.72 | 4 | 2 | 2 | 2 | 1.046 | 1.107 | 1.056 | 1.156 | 1.102 | 1.040 |
| A6PVN9 | A6PVN9 | 6.08 | 10 | 2 | 2 | 3 | 1.088 | 1.106 | 1.102 | 1.050 | 1.019 | 1.000 |
| O75844 | FACE1 | 5.68 | 1 | 2 | 2 | 2 | 1.109 | 1.104 | 0.994 | 1.349 | 1.002 | 1.219 |
| B4DWJ2 | B4DWJ2 | 7.72 | 7 | 5 | 5 | 7 | 1.007 | 1.103 | 1.045 | 1.049 | 0.990 | 0.919 |
| O43615 | TIM44 | 18.81 | 1 | 7 | 7 | 18 | 1.106 | 1.103 | 1.011 | 1.111 | 1.051 | 1.044 |
| Q5URX0 | Q5URX0 | 6.65 | 2 | 2 | 2 | 2 | 0.961 | 1.102 | 1.145 | 0.884 | 0.916 | 0.798 |
| Q8WVJ2 | NUDC2 | 22.93 | 2 | 3 | 3 | 3 | 1.248 | 1.101 | 0.881 | 1.037 | 0.829 | 0.938 |
| Q08379 | GOGA2 | 1.70 | 2 | 2 | 2 | 4 | 0.895 | 1.101 | 1.144 | 0.989 | 1.110 | 1.020 |
| P48047 | ATPO | 33.33 | 1 | 5 | 5 | 22 | 1.044 | 1.100 | 1.059 | 1.075 | 1.011 | 0.966 |
| P26196 | DDX6 | 7.87 | 2 | 3 | 3 | 4 | 1.047 | 1.099 | 1.063 | 1.062 | 1.011 | 0.958 |
| B7Z1R5 | B7Z1R5 | 23.63 | 5 | 10 | 10 | 28 | 1.053 | 1.098 | 1.047 | 0.942 | 0.960 | 0.890 |
| Q02818 | NUCB1 | 7.16 | 2 | 3 | 3 | 21 | 0.910 | 1.097 | 1.148 | 1.098 | 1.135 | 0.960 |
| P22314 | UBA1 | 10.59 | 13 | 8 | 8 | 17 | 0.953 | 1.097 | 1.114 | 1.073 | 1.039 | 0.988 |
| Q04917 | 1433F | 41.87 | 6 | 5 | 10 | 76 | 1.157 | 1.097 | 0.981 | 1.278 | 1.187 | 1.087 |
| Q7LBC6 | KDM3B | 2.27 | 2 | 3 | 3 | 3 | 0.861 | 1.096 | 1.271 | 0.982 | 1.137 | 0.891 |
| Q10570 | CPSF1 | 4.44 | 2 | 5 | 5 | 5 | 1.008 | 1.096 | 1.156 | 0.830 | 0.928 | 0.664 |
| E7ESU4 | E7ESU4 | 9.97 | 3 | 7 | 7 | 10 | 1.018 | 1.095 | 1.048 | 0.979 | 1.046 | 0.982 |
| P08758 | ANXA5 | 34.69 | 6 | 10 | 11 | 43 | 0.939 | 1.095 | 1.157 | 1.191 | 1.253 | 1.067 |
| B4DG62 | B4DG62 | 7.76 | 6 | 4 | 6 | 14 | 0.971 | 1.093 | 1.070 | 0.968 | 1.072 | 0.861 |
| Q92945 | FUBP2 | 21.10 | 9 | 13 | 15 | 35 | 0.964 | 1.093 | 1.085 | 1.108 | 1.093 | 0.981 |
| P16615 | AT2A2 | 5.85 | 3 | 3 | 3 | 4 | 1.238 | 1.093 | 0.799 | 1.099 | 1.003 | 0.837 |
| Q9BWS9 | CHID1 | 6.11 | 6 | 2 | 2 | 2 | 1.012 | 1.092 | 1.077 | 0.983 | 0.968 | 0.896 |
| P51149 | RAB7A | 47.83 | 7 | 8 | 8 | 33 | 0.975 | 1.091 | 1.070 | 0.988 | 0.996 | 0.957 |
| P54727 | RD23B | 14.43 | 4 | 5 | 5 | 11 | 0.962 | 1.090 | 1.102 | 1.026 | 1.043 | 0.932 |
| E7EQT4 | E7EQT4 | 2.31 | 4 | 3 | 3 | 6 | 1.026 | 1.090 | 1.163 | 1.003 | 1.066 | 0.936 |
| E7EUB4 | E7EUB4 | 5.42 | 2 | 3 | 3 | 3 | 0.942 | 1.090 | 1.108 | 0.849 | 0.862 | 0.775 |
| Q92922 | SMRC1 | 3.62 | 4 | 2 | 4 | 5 | 1.168 | 1.089 | 0.931 | 1.138 | 0.971 | 1.040 |
| Q99856 | ARI3A | 9.78 | 2 | 4 | 4 | 10 | 1.019 | 1.089 | 1.104 | 1.181 | 1.130 | 0.985 |
| B4DMT5 | B4DMT5 | 19.54 | 4 | 5 | 5 | 12 | 1.058 | 1.089 | 0.955 | 1.059 | 1.025 | 0.967 |
| Q9BXJ9 | NAA15 | 16.74 | 3 | 12 | 12 | 22 | 1.034 | 1.088 | 0.994 | 1.044 | 0.965 | 0.981 |
| P35237 | SPB6 | 25.80 | 4 | 8 | 8 | 14 | 0.940 | 1.087 | 1.122 | 1.102 | 1.081 | 0.934 |
| Q9H9B4 | SFXN1 | 16.46 | 3 | 4 | 4 | 13 | 1.225 | 1.087 | 0.931 | 1.378 | 1.118 | 1.097 |
| B4DT43 | B4DT43 | 25.35 | 2 | 5 | 5 | 16 | 0.944 | 1.087 | 1.181 | 1.075 | 1.066 | 0.950 |
| E7ERZ4 | E7ERZ4 | 10.42 | 5 | 3 | 3 | 5 | 0.937 | 1.087 | 1.149 | 1.048 | 1.044 | 0.896 |
| F2Z2K0 | F2Z2K0 | 9.85 | 2 | 2 | 2 | 2 | 1.253 | 1.087 | 0.866 | 1.127 | 0.897 | 1.032 |
| Q9Y3I0 | RTCB | 17.03 | 4 | 7 | 7 | 15 | 1.004 | 1.085 | 1.074 | 1.072 | 1.021 | 0.974 |
| A8KA46 | A8KA46 | 23.21 | 3 | 3 | 3 | 5 | 1.277 | 1.085 | 0.919 | 1.362 | 1.006 | 1.120 |
| Q9Y3A5 | SBDS | 15.60 | 1 | 4 | 4 | 5 | 0.917 | 1.084 | 1.121 | 1.095 | 1.128 | 0.926 |
| Q6UYC3 | Q6UYC3 | 35.50 | 7 | 21 | 24 | 59 | 1.027 | 1.084 | 1.052 | 1.077 | 1.071 | 0.997 |
| Q53GQ0 | DHB12 | 11.86 | 3 | 3 | 3 | 3 | 0.932 | 1.083 | 1.172 | 0.812 | 0.877 | 0.746 |
| B4E0K5 | B4E0K5 | 16.96 | 4 | 3 | 3 | 5 | 1.017 | 1.083 | 1.208 | 0.997 | 1.085 | 0.926 |
| Q92841 | DDX17 | 23.54 | 2 | 10 | 14 | 55 | 1.006 | 1.083 | 1.080 | 1.060 | 1.039 | 0.986 |
| Q9BR76 | COR1B | 3.48 | 2 | 2 | 2 | 2 | 0.910 | 1.083 | 1.188 | 1.070 | 1.172 | 0.983 |
| B4DWN1 | B4DWN1 | 11.23 | 4 | 2 | 2 | 2 | 1.140 | 1.083 | 0.948 | 0.875 | 0.766 | 0.805 |
| Q13907 | IDI1 | 13.66 | 3 | 2 | 2 | 6 | 1.070 | 1.083 | 1.019 | 0.929 | 0.873 | 0.854 |
| P35219 | CAH8 | 11.03 | 1 | 2 | 2 | 2 | 0.771 | 1.082 | 1.401 | 1.015 | 1.312 | 0.933 |
| P15311 | EZRI | 30.55 | 3 | 13 | 19 | 90 | 1.026 | 1.082 | 1.051 | 1.141 | 1.111 | 1.010 |
| Q9Y4W6 | AFG32 | 2.38 | 1 | 2 | 2 | 4 | 0.921 | 1.081 | 1.081 | 2.106 | 2.093 | 1.938 |
| B4DHG0 | B4DHG0 | 18.29 | 5 | 6 | 6 | 12 | 0.962 | 1.081 | 1.126 | 1.013 | 1.109 | 0.995 |
| Q9BQE3 | TBA1C | 47.22 | 27 | 4 | 18 | 233 | 1.047 | 1.081 | 1.051 | 1.082 | 1.096 | 1.020 |
| F8W7K3 | F8W7K3 | 5.91 | 5 | 12 | 12 | 13 | 1.059 | 1.081 | 1.060 | 1.147 | 1.150 | 1.040 |
| Q8WYA6 | CTBL1 | 4.97 | 1 | 3 | 3 | 4 | 0.902 | 1.080 | 1.198 | 0.880 | 0.939 | 0.809 |
| B4DZ87 | B4DZ87 | 9.58 | 2 | 5 | 5 | 5 | 1.059 | 1.080 | 1.072 | 1.029 | 0.968 | 0.982 |
| P51858 | HDGF | 17.92 | 9 | 3 | 4 | 15 | 0.980 | 1.080 | 1.005 | 0.952 | 0.962 | 0.965 |
| B4DJV2 | B4DJV2 | 18.32 | 15 | 7 | 7 | 32 | 1.045 | 1.080 | 1.009 | 1.057 | 0.988 | 1.026 |
| P14735 | IDE | 6.08 | 2 | 6 | 6 | 16 | 0.956 | 1.078 | 1.168 | 1.006 | 1.085 | 0.962 |
| O60888 | CUTA | 29.61 | 2 | 3 | 3 | 4 | 1.154 | 1.078 | 0.933 | 0.956 | 0.827 | 0.883 |
| Q13098 | CSN1 | 7.74 | 2 | 3 | 3 | 3 | 1.031 | 1.077 | 0.991 | 1.065 | 0.977 | 0.983 |
| P35580 | MYH10 | 10.02 | 7 | 9 | 17 | 26 | 0.939 | 1.077 | 1.172 | 1.101 | 1.190 | 1.015 |
| P37108 | SRP14 | 16.91 | 1 | 2 | 2 | 13 | 1.085 | 1.077 | 0.966 | 1.165 | 1.044 | 1.081 |
| P40763 | STAT3 | 6.36 | 1 | 4 | 4 | 5 | 1.127 | 1.076 | 1.004 | 1.092 | 1.064 | 0.931 |
| Q14019 | COTL1 | 32.39 | 1 | 5 | 5 | 6 | 1.101 | 1.076 | 1.003 | 1.094 | 1.016 | 1.025 |
| F8W181 | F8W181 | 15.93 | 4 | 3 | 3 | 26 | 1.695 | 1.076 | 0.697 | 1.613 | 0.869 | 1.436 |
| B2WTI4 | B2WTI4 | 9.42 | 2 | 3 | 3 | 3 | 1.134 | 1.076 | 0.822 | 0.994 | 0.887 | 0.987 |
| P51665 | PSD7 | 11.11 | 2 | 3 | 3 | 28 | 1.020 | 1.076 | 0.996 | 1.052 | 1.075 | 1.048 |
| Q9BXK5 | B2L13 | 5.15 | 5 | 2 | 2 | 3 | 1.015 | 1.075 | 1.057 | 0.967 | 1.120 | 0.940 |
| Q9NZ01 | TECR | 11.69 | 2 | 4 | 4 | 7 | 1.281 | 1.075 | 0.929 | 1.080 | 0.910 | 0.976 |
| E7EQK9 | E7EQK9 | 7.88 | 4 | 4 | 4 | 6 | 0.788 | 1.074 | 1.361 | 0.887 | 1.040 | 0.935 |
| F8W6E4 | F8W6E4 | 3.20 | 3 | 2 | 2 | 7 | 1.046 | 1.074 | 1.077 | 1.217 | 1.154 | 1.165 |
| E9PC28 | E9PC28 | 1.91 | 7 | 2 | 2 | 3 | 0.928 | 1.074 | 1.267 | 0.951 | 1.070 | 0.842 |
| P48637 | GSHB | 16.24 | 3 | 5 | 5 | 5 | 1.146 | 1.074 | 1.017 | 1.105 | 1.016 | 0.987 |
| B7Z9I1 | B7Z9I1 | 13.25 | 6 | 4 | 4 | 20 | 0.967 | 1.074 | 1.095 | 1.049 | 1.154 | 1.040 |
| Q15102 | PA1B3 | 7.36 | 1 | 2 | 2 | 9 | 0.836 | 1.073 | 1.248 | 1.083 | 1.166 | 0.944 |
| Q00610 | CLH1 | 23.16 | 3 | 33 | 33 | 171 | 0.960 | 1.073 | 1.086 | 1.007 | 1.063 | 0.951 |
| F5H4W8 | F5H4W8 | 13.82 | 2 | 2 | 2 | 2 | 0.882 | 1.073 | 1.215 | 0.886 | 1.002 | 0.822 |
| C9JQD4 | C9JQD4 | 27.78 | 5 | 3 | 5 | 17 | 1.209 | 1.073 | 0.896 | 1.104 | 0.924 | 1.039 |
| F2Z2Y6 | F2Z2Y6 | 38.81 | 4 | 2 | 2 | 6 | 0.991 | 1.073 | 1.095 | 1.057 | 1.044 | 1.030 |
| E9PKG1 | E9PKG1 | 13.23 | 7 | 4 | 4 | 4 | 0.925 | 1.073 | 1.130 | 1.028 | 1.038 | 0.954 |
| P31930 | QCR1 | 12.50 | 1 | 6 | 6 | 10 | 1.020 | 1.073 | 1.055 | 0.990 | 1.009 | 0.919 |
| Q99436 | PSB7 | 28.52 | 3 | 6 | 6 | 9 | 1.015 | 1.072 | 1.193 | 1.160 | 1.158 | 0.979 |
| P85037 | FOXK1 | 3.41 | 2 | 2 | 2 | 2 | 0.823 | 1.072 | 1.300 | 1.052 | 1.274 | 0.976 |
| Q13838 | DX39B | 30.37 | 23 | 4 | 13 | 96 | 1.062 | 1.071 | 1.006 | 1.017 | 1.016 | 0.993 |
| P46781 | RS9 | 30.41 | 7 | 6 | 7 | 15 | 1.373 | 1.071 | 0.755 | 1.241 | 0.914 | 1.272 |
| P46060 | RAGP1 | 9.20 | 4 | 4 | 4 | 5 | 1.050 | 1.071 | 0.961 | 0.960 | 0.941 | 0.976 |
| P37837 | TALDO | 34.42 | 4 | 12 | 12 | 82 | 1.005 | 1.071 | 1.084 | 1.105 | 1.109 | 1.031 |
| C9J8H9 | C9J8H9 | 48.98 | 7 | 2 | 2 | 14 | 1.198 | 1.070 | 0.844 | 1.029 | 0.848 | 1.003 |
| Q9HC38 | GLOD4 | 7.67 | 3 | 3 | 3 | 6 | 0.971 | 1.070 | 1.052 | 1.039 | 0.999 | 0.996 |
| Q14204 | DYHC1 | 7.32 | 1 | 30 | 30 | 44 | 0.954 | 1.069 | 1.108 | 0.993 | 1.041 | 0.975 |
| B1ALW1 | B1ALW1 | 38.82 | 2 | 4 | 4 | 17 | 1.108 | 1.069 | 0.972 | 1.093 | 0.991 | 1.011 |
| Q15393 | SF3B3 | 15.61 | 1 | 14 | 14 | 23 | 1.135 | 1.069 | 1.024 | 0.990 | 0.989 | 0.963 |
| Q8IX01 | SUGP2 | 2.50 | 1 | 2 | 2 | 2 | 0.930 | 1.069 | 1.147 | 0.998 | 1.070 | 0.930 |
| B7Z1I0 | B7Z1I0 | 7.55 | 3 | 2 | 2 | 9 | 0.917 | 1.069 | 1.162 | 1.034 | 1.072 | 0.919 |
| E9PBF6 | E9PBF6 | 54.26 | 2 | 20 | 23 | 55 | 1.021 | 1.069 | 1.032 | 0.997 | 0.974 | 0.953 |
| P40939 | ECHA | 26.21 | 2 | 17 | 17 | 83 | 1.002 | 1.069 | 1.074 | 1.094 | 1.063 | 1.024 |
| Q96HR9 | REEP6 | 11.41 | 2 | 2 | 2 | 12 | 0.977 | 1.068 | 1.116 | 1.109 | 1.190 | 1.104 |
| B4DDF7 | B4DDF7 | 22.11 | 14 | 11 | 11 | 52 | 1.018 | 1.067 | 1.012 | 1.034 | 1.052 | 1.014 |
| F5H265 | F5H265 | 79.19 | 19 | 7 | 7 | 113 | 1.375 | 1.067 | 0.776 | 1.199 | 0.867 | 1.146 |
| E9PGT0 | E9PGT0 | 1.51 | 7 | 2 | 3 | 4 | 1.140 | 1.067 | 0.934 | 1.553 | 1.357 | 1.448 |
| P12829 | MYL4 | 12.18 | 2 | 2 | 2 | 3 | 1.061 | 1.067 | 0.988 | 1.051 | 0.988 | 0.996 |
| F5H769 | F5H769 | 7.32 | 9 | 7 | 7 | 9 | 1.002 | 1.067 | 1.062 | 0.946 | 1.013 | 0.993 |
| P33993 | MCM7 | 32.82 | 5 | 17 | 17 | 53 | 1.036 | 1.067 | 1.059 | 1.004 | 1.037 | 0.975 |
| Q9NX55 | HYPK | 43.41 | 1 | 5 | 5 | 10 | 0.957 | 1.067 | 0.940 | 1.084 | 0.933 | 1.032 |
| E7EVE6 | E7EVE6 | 34.25 | 4 | 6 | 6 | 31 | 0.970 | 1.066 | 1.039 | 1.036 | 0.953 | 0.948 |
| P06730 | IF4E | 9.68 | 5 | 2 | 2 | 12 | 1.019 | 1.066 | 1.026 | 0.964 | 0.980 | 0.965 |
| Q13951 | PEBB | 9.34 | 1 | 2 | 2 | 2 | 1.043 | 1.066 | 1.020 | 1.043 | 0.996 | 0.974 |
| F5H8C7 | F5H8C7 | 2.83 | 5 | 2 | 2 | 4 | 0.959 | 1.065 | 1.300 | 1.191 | 1.250 | 1.015 |
| E7EW34 | E7EW34 | 19.92 | 4 | 12 | 12 | 50 | 0.898 | 1.065 | 1.246 | 1.015 | 1.102 | 0.936 |
| P23193 | TCEA1 | 6.98 | 1 | 2 | 2 | 2 | 0.995 | 1.065 | 1.068 | 0.953 | 0.955 | 0.891 |
| O43396 | TXNL1 | 14.53 | 2 | 4 | 4 | 19 | 0.967 | 1.064 | 1.073 | 1.006 | 0.994 | 0.962 |
| O95881 | TXD12 | 16.86 | 1 | 2 | 2 | 2 | 1.121 | 1.063 | 0.947 | 1.209 | 1.075 | 1.131 |
| O00410 | IPO5 | 8.30 | 7 | 5 | 7 | 22 | 1.110 | 1.063 | 0.990 | 1.015 | 0.960 | 0.922 |
| Q9H3U1 | UN45A | 3.39 | 1 | 2 | 2 | 2 | 0.809 | 1.063 | 1.312 | 1.042 | 1.285 | 0.976 |
| P62714 | PP2AB | 6.15 | 3 | 2 | 2 | 3 | 1.033 | 1.062 | 1.026 | 1.159 | 1.197 | 0.978 |
| P07384 | CAN1 | 8.68 | 11 | 5 | 5 | 6 | 1.039 | 1.062 | 1.110 | 1.075 | 1.148 | 1.012 |
| Q8WXX5 | DNJC9 | 22.31 | 1 | 4 | 4 | 4 | 0.962 | 1.062 | 1.098 | 1.083 | 1.092 | 1.018 |
| Q99623 | PHB2 | 49.83 | 9 | 12 | 12 | 91 | 0.991 | 1.061 | 1.033 | 0.980 | 0.967 | 0.951 |
| E9PM46 | E9PM46 | 1.48 | 2 | 2 | 2 | 2 | 1.009 | 1.061 | 1.050 | 1.069 | 1.056 | 1.003 |
| P62191 | PRS4 | 19.55 | 2 | 7 | 7 | 27 | 0.970 | 1.061 | 1.081 | 0.969 | 1.026 | 0.948 |
| Q9BWF3 | RBM4 | 18.68 | 7 | 7 | 7 | 7 | 1.071 | 1.060 | 1.027 | 1.021 | 1.043 | 0.999 |
| P25685 | DNJB1 | 7.94 | 2 | 3 | 3 | 3 | 1.062 | 1.060 | 0.922 | 0.911 | 0.889 | 0.961 |
| F5H1W9 | F5H1W9 | 8.05 | 5 | 2 | 2 | 2 | 1.039 | 1.059 | 1.018 | 0.998 | 0.958 | 0.938 |
| E5RHW4 | E5RHW4 | 11.54 | 5 | 5 | 5 | 7 | 1.018 | 1.058 | 1.045 | 1.005 | 1.035 | 0.968 |
| Q14974 | IMB1 | 17.69 | 3 | 12 | 12 | 51 | 0.975 | 1.058 | 1.070 | 0.979 | 1.035 | 0.921 |
| P14550 | AK1A1 | 7.69 | 1 | 2 | 2 | 3 | 0.956 | 1.057 | 1.104 | 1.203 | 1.140 | 1.014 |
| A6NNK5 | A6NNK5 | 1.14 | 4 | 2 | 2 | 2 | 0.958 | 1.057 | 1.102 | 1.101 | 1.146 | 1.036 |
| F5GX33 | F5GX33 | 9.17 | 5 | 4 | 4 | 5 | 1.170 | 1.056 | 0.867 | 1.155 | 1.020 | 1.139 |
| Q9NYU2 | UGGG1 | 3.41 | 1 | 4 | 4 | 7 | 1.015 | 1.056 | 0.986 | 1.074 | 1.055 | 1.006 |
| P60900 | PSA6 | 16.26 | 7 | 3 | 3 | 15 | 1.028 | 1.056 | 1.051 | 1.032 | 1.036 | 0.985 |
| Q9BUQ8 | DDX23 | 4.51 | 2 | 4 | 4 | 5 | 1.044 | 1.056 | 1.020 | 0.844 | 0.804 | 0.787 |
| P08727 | K1C19 | 14.50 | 2 | 5 | 5 | 6 | 1.060 | 1.056 | 0.938 | 1.012 | 0.927 | 0.954 |
| O95831 | AIFM1 | 13.87 | 5 | 6 | 6 | 10 | 0.943 | 1.055 | 1.014 | 1.043 | 1.068 | 1.017 |
| Q9HBB2 | Q9HBB2 | 3.29 | 3 | 2 | 2 | 2 | 0.930 | 1.055 | 1.133 | 0.882 | 0.946 | 0.832 |
| P11233 | RALA | 13.11 | 8 | 2 | 2 | 4 | 1.192 | 1.055 | 0.871 | 1.096 | 0.912 | 1.043 |
| Q5T3Q7 | Q5T3Q7 | 1.99 | 2 | 3 | 3 | 5 | 0.852 | 1.055 | 0.951 | 0.758 | 0.680 | 0.578 |
| P32969 | RL9 | 19.27 | 4 | 5 | 5 | 18 | 1.060 | 1.055 | 0.951 | 1.067 | 0.895 | 0.927 |
| B4DUW6 | B4DUW6 | 10.33 | 4 | 4 | 4 | 4 | 0.915 | 1.055 | 1.120 | 1.172 | 1.243 | 1.106 |
| Q15046 | SYK | 15.75 | 3 | 10 | 10 | 18 | 1.007 | 1.054 | 1.014 | 1.075 | 1.050 | 1.060 |
| O60306 | AQR | 5.39 | 1 | 5 | 6 | 7 | 0.949 | 1.054 | 1.239 | 1.028 | 1.067 | 0.972 |
| P07954 | FUMH | 14.31 | 1 | 6 | 6 | 14 | 0.919 | 1.054 | 1.051 | 1.038 | 0.989 | 0.916 |
| P23258 | TBG1 | 7.76 | 2 | 3 | 3 | 3 | 0.971 | 1.054 | 1.083 | 0.964 | 0.948 | 0.872 |
| Q99460 | PSMD1 | 11.65 | 1 | 7 | 7 | 42 | 0.956 | 1.053 | 1.109 | 1.070 | 1.099 | 1.003 |
| B4DEB1 | B4DEB1 | 21.14 | 7 | 4 | 4 | 59 | 1.222 | 1.053 | 0.862 | 1.385 | 1.111 | 1.309 |
| P00491 | PNPH | 24.91 | 5 | 6 | 6 | 7 | 1.114 | 1.053 | 0.913 | 1.037 | 0.883 | 0.964 |
| E7EQD1 | E7EQD1 | 18.65 | 2 | 4 | 4 | 11 | 0.988 | 1.051 | 1.096 | 1.068 | 1.089 | 0.991 |
| P16949 | STMN1 | 45.64 | 12 | 9 | 9 | 48 | 0.996 | 1.051 | 1.061 | 1.107 | 1.133 | 1.020 |
| Q9Y2Z0 | SUGT1 | 9.32 | 2 | 3 | 3 | 3 | 0.945 | 1.050 | 1.043 | 0.982 | 0.938 | 0.807 |
| Q13547 | HDAC1 | 14.73 | 3 | 3 | 6 | 15 | 0.954 | 1.050 | 1.164 | 1.098 | 1.186 | 0.985 |
| B4DEJ6 | B4DEJ6 | 11.52 | 5 | 5 | 5 | 7 | 1.046 | 1.049 | 1.082 | 0.992 | 0.929 | 0.952 |
| P78527 | PRKDC | 5.47 | 3 | 19 | 19 | 29 | 1.065 | 1.049 | 0.969 | 0.957 | 0.924 | 0.942 |
| Q9ULC4 | MCTS1 | 13.81 | 1 | 2 | 2 | 15 | 0.988 | 1.049 | 1.091 | 1.109 | 1.106 | 1.115 |
| P30042 | ES1 | 7.46 | 2 | 2 | 2 | 2 | 1.098 | 1.048 | 0.953 | 1.226 | 1.113 | 1.164 |
| E7EUT8 | E7EUT8 | 10.94 | 2 | 4 | 4 | 4 | 1.076 | 1.048 | 1.122 | 0.791 | 0.813 | 0.783 |
| Q99733 | NP1L4 | 17.07 | 11 | 5 | 6 | 38 | 0.880 | 1.048 | 1.409 | 1.006 | 1.078 | 0.998 |
| E7EM48 | E7EM48 | 17.74 | 6 | 12 | 12 | 62 | 0.946 | 1.047 | 1.076 | 1.028 | 1.116 | 1.001 |
| P28066 | PSA5 | 29.05 | 2 | 6 | 6 | 29 | 1.008 | 1.047 | 1.070 | 1.022 | 0.970 | 0.961 |
| Q03252 | LMNB2 | 18.17 | 1 | 10 | 14 | 15 | 0.999 | 1.047 | 1.042 | 1.033 | 1.051 | 0.967 |
| P30419 | NMT1 | 12.30 | 8 | 5 | 5 | 10 | 1.062 | 1.047 | 0.954 | 1.003 | 0.870 | 0.972 |
| B4DDD7 | B4DDD7 | 14.08 | 4 | 3 | 3 | 7 | 0.964 | 1.047 | 1.128 | 1.056 | 0.936 | 0.881 |
| P06865 | HEXA | 7.75 | 3 | 4 | 4 | 6 | 0.928 | 1.046 | 1.197 | 1.226 | 1.310 | 1.110 |
| Q9NUU7 | DD19A | 13.39 | 7 | 5 | 5 | 5 | 1.184 | 1.046 | 0.882 | 1.085 | 0.914 | 0.986 |
| P27635 | RL10 | 21.96 | 4 | 6 | 6 | 21 | 1.308 | 1.046 | 0.798 | 1.348 | 0.998 | 1.237 |
| B4E327 | B4E327 | 9.69 | 6 | 3 | 3 | 3 | 1.098 | 1.045 | 0.958 | 1.069 | 0.968 | 1.054 |
| Q8NE71 | ABCF1 | 11.95 | 7 | 9 | 9 | 24 | 1.078 | 1.045 | 1.004 | 1.111 | 1.007 | 0.988 |
| Q13151 | ROA0 | 14.43 | 1 | 3 | 5 | 13 | 1.157 | 1.045 | 0.917 | 1.135 | 0.978 | 1.041 |
| Q9H307 | PININ | 4.18 | 1 | 3 | 3 | 4 | 1.145 | 1.045 | 0.911 | 1.085 | 0.982 | 0.962 |
| B9A067 | B9A067 | 14.49 | 8 | 9 | 9 | 14 | 1.020 | 1.045 | 1.058 | 1.050 | 1.098 | 1.063 |
| D6RFM5 | D6RFM5 | 4.46 | 4 | 2 | 2 | 20 | 0.940 | 1.044 | 1.118 | 1.069 | 1.097 | 0.967 |
| D3YLG5 | D3YLG5 | 27.27 | 3 | 4 | 4 | 4 | 0.973 | 1.044 | 0.997 | 1.084 | 0.981 | 0.963 |
| B4E022 | B4E022 | 24.13 | 6 | 11 | 11 | 47 | 1.036 | 1.044 | 0.993 | 1.044 | 0.974 | 1.067 |
| P42345 | MTOR | 2.20 | 4 | 4 | 4 | 4 | 0.901 | 1.044 | 1.148 | 1.066 | 1.109 | 0.972 |
| Q7Z451 | Q7Z451 | 12.05 | 3 | 5 | 7 | 17 | 0.945 | 1.044 | 1.083 | 1.142 | 1.039 | 0.996 |
| Q9Y224 | CN166 | 28.69 | 3 | 6 | 6 | 19 | 0.911 | 1.044 | 1.170 | 0.970 | 1.106 | 1.073 |
| Q12788 | TBL3 | 3.59 | 1 | 2 | 2 | 7 | 0.875 | 1.044 | 1.128 | 0.884 | 0.990 | 0.891 |
| Q13242 | SRSF9 | 22.62 | 1 | 5 | 5 | 15 | 0.961 | 1.043 | 1.036 | 0.988 | 0.935 | 0.885 |
| P25205 | MCM3 | 22.52 | 2 | 14 | 14 | 63 | 0.955 | 1.043 | 1.065 | 0.974 | 1.044 | 0.985 |
| P78417 | GSTO1 | 24.48 | 5 | 7 | 7 | 54 | 0.959 | 1.042 | 1.062 | 1.119 | 1.130 | 1.049 |
| P49916 | DNLI3 | 2.58 | 1 | 2 | 2 | 2 | 1.043 | 1.042 | 0.997 | 0.914 | 0.873 | 0.873 |
| B4DLR8 | B4DLR8 | 13.86 | 3 | 2 | 2 | 2 | 0.927 | 1.042 | 1.122 | 1.088 | 1.169 | 1.039 |
| C9J8E1 | C9J8E1 | 4.82 | 6 | 2 | 2 | 2 | 0.989 | 1.041 | 1.051 | 1.063 | 1.072 | 1.016 |
| Q92918 | M4K1 | 2.16 | 1 | 2 | 2 | 2 | 1.029 | 1.040 | 1.010 | 1.142 | 1.106 | 1.092 |
| P49593 | PPM1F | 15.42 | 6 | 4 | 4 | 12 | 0.933 | 1.040 | 1.065 | 0.954 | 0.997 | 1.007 |
| Q16658 | FSCN1 | 25.35 | 4 | 10 | 10 | 80 | 1.048 | 1.040 | 0.988 | 1.019 | 0.969 | 0.970 |
| Q92621 | NU205 | 1.74 | 2 | 4 | 4 | 4 | 1.053 | 1.040 | 0.997 | 1.077 | 1.062 | 1.061 |
| G3XAL7 | G3XAL7 | 6.52 | 4 | 2 | 2 | 2 | 0.938 | 1.039 | 1.107 | 0.808 | 0.859 | 0.774 |
| P62316 | SMD2 | 28.81 | 1 | 4 | 4 | 13 | 1.071 | 1.039 | 0.948 | 1.063 | 0.950 | 1.018 |
| B3KXS5 | B3KXS5 | 14.30 | 3 | 14 | 14 | 50 | 1.029 | 1.038 | 1.005 | 1.021 | 1.024 | 1.015 |
| P35241 | RADI | 27.62 | 10 | 9 | 15 | 76 | 0.988 | 1.038 | 1.067 | 1.028 | 1.097 | 0.991 |
| B4DR70 | B4DR70 | 11.89 | 6 | 5 | 5 | 17 | 1.025 | 1.038 | 1.123 | 1.046 | 1.017 | 1.054 |
| B1AKP7 | B1AKP7 | 7.12 | 4 | 2 | 2 | 18 | 1.001 | 1.037 | 1.112 | 0.965 | 1.020 | 0.967 |
| P51572 | BAP31 | 13.01 | 5 | 3 | 3 | 3 | 1.072 | 1.037 | 0.966 | 0.937 | 0.872 | 0.899 |
| B4E363 | B4E363 | 7.97 | 2 | 3 | 3 | 11 | 0.949 | 1.037 | 1.215 | 0.946 | 0.962 | 0.808 |
| Q9Y237 | PIN4 | 22.90 | 2 | 3 | 3 | 10 | 1.005 | 1.037 | 0.992 | 1.110 | 1.110 | 1.087 |
| Q9UL46 | PSME2 | 14.64 | 3 | 3 | 3 | 11 | 0.953 | 1.036 | 1.007 | 1.048 | 1.040 | 1.041 |
| B4DZM8 | B4DZM8 | 21.04 | 3 | 8 | 8 | 14 | 0.945 | 1.036 | 1.052 | 0.986 | 1.005 | 0.983 |
| F8W9S7 | F8W9S7 | 1.56 | 5 | 2 | 2 | 2 | 0.943 | 1.036 | 1.096 | 1.066 | 1.127 | 1.024 |
| C9JTZ5 | C9JTZ5 | 24.76 | 11 | 2 | 2 | 2 | 1.000 | 1.035 | 1.034 | 0.970 | 0.967 | 0.932 |
| Q9UHD8 | sept-09 | 7.68 | 2 | 4 | 4 | 4 | 0.992 | 1.035 | 1.170 | 1.049 | 1.044 | 1.004 |
| F5GWT9 | F5GWT9 | 8.97 | 2 | 5 | 5 | 6 | 0.987 | 1.035 | 0.989 | 1.098 | 0.964 | 0.931 |
| Q9Y490 | TLN1 | 19.56 | 2 | 40 | 40 | 111 | 1.024 | 1.035 | 1.018 | 1.009 | 0.975 | 0.993 |
| F5GYN4 | F5GYN4 | 10.37 | 5 | 2 | 2 | 3 | 0.886 | 1.034 | 1.147 | 1.051 | 1.091 | 0.862 |
| F5H2U1 | F5H2U1 | 6.48 | 4 | 3 | 3 | 3 | 1.112 | 1.034 | 0.933 | 1.068 | 0.960 | 1.028 |
| P24539 | AT5F1 | 12.11 | 2 | 3 | 3 | 22 | 1.039 | 1.034 | 1.036 | 0.996 | 1.023 | 0.940 |
| O14929 | HAT1 | 10.98 | 1 | 4 | 4 | 5 | 1.101 | 1.034 | 1.096 | 0.956 | 1.029 | 0.936 |
| E7EU42 | E7EU42 | 5.66 | 2 | 2 | 2 | 5 | 1.052 | 1.034 | 1.081 | 1.344 | 1.273 | 1.725 |
| P45973 | CBX5 | 9.95 | 2 | 2 | 2 | 2 | 0.955 | 1.033 | 1.080 | 0.924 | 0.964 | 0.890 |
| P10606 | COX5B | 18.60 | 1 | 2 | 2 | 2 | 0.937 | 1.033 | 1.101 | 1.081 | 1.150 | 1.041 |
| F8VXY0 | F8VXY0 | 39.14 | 11 | 8 | 13 | 157 | 1.033 | 1.033 | 1.001 | 1.039 | 0.988 | 1.005 |
| Q9NT62 | ATG3 | 10.51 | 2 | 3 | 3 | 6 | 1.111 | 1.033 | 0.923 | 0.987 | 0.845 | 0.893 |
| F5H2T0 | F5H2T0 | 2.24 | 2 | 2 | 2 | 2 | 1.053 | 1.033 | 0.979 | 0.939 | 0.889 | 0.905 |
| P25786 | PSA1 | 16.35 | 3 | 4 | 4 | 7 | 1.085 | 1.032 | 1.051 | 1.172 | 1.039 | 0.983 |
| Q96DG6 | CMBL | 13.47 | 1 | 3 | 3 | 10 | 1.116 | 1.032 | 1.114 | 1.173 | 1.126 | 1.140 |
| P62314 | SMD1 | 36.97 | 1 | 3 | 3 | 25 | 1.233 | 1.031 | 0.847 | 1.360 | 1.074 | 1.064 |
| F5H6K0 | F5H6K0 | 14.80 | 2 | 9 | 9 | 43 | 0.995 | 1.031 | 1.097 | 0.983 | 1.036 | 0.966 |
| D6RC06 | D6RC06 | 32.31 | 5 | 2 | 2 | 4 | 0.997 | 1.030 | 1.044 | 0.996 | 0.985 | 0.967 |
| A6NN80 | A6NN80 | 22.79 | 12 | 13 | 13 | 28 | 0.940 | 1.029 | 1.081 | 1.092 | 1.158 | 1.026 |
| E7EVW7 | E7EVW7 | 5.57 | 3 | 2 | 2 | 2 | 1.044 | 1.029 | 0.984 | 0.911 | 0.870 | 0.881 |
| P62158 | CALM | 28.19 | 11 | 4 | 4 | 10 | 1.179 | 1.029 | 0.971 | 1.030 | 0.980 | 0.975 |
| O60884 | DNJA2 | 7.28 | 1 | 3 | 3 | 7 | 1.080 | 1.028 | 0.969 | 0.984 | 0.911 | 0.980 |
| F5GZL7 | F5GZL7 | 1.15 | 15 | 4 | 4 | 4 | 1.083 | 1.028 | 0.967 | 0.986 | 0.885 | 0.884 |
| A8K1B6 | A8K1B6 | 7.74 | 10 | 6 | 6 | 11 | 1.019 | 1.027 | 0.975 | 1.091 | 1.099 | 1.011 |
| P06576 | ATPB | 53.12 | 5 | 20 | 20 | 262 | 0.999 | 1.027 | 1.051 | 1.040 | 1.046 | 0.987 |
| E9PKH6 | E9PKH6 | 27.54 | 5 | 4 | 4 | 23 | 0.976 | 1.026 | 1.022 | 0.955 | 0.980 | 0.953 |
| O75531 | BAF | 21.35 | 1 | 2 | 2 | 26 | 0.929 | 1.026 | 1.049 | 1.046 | 1.114 | 1.037 |
| Q04828 | AK1C1 | 19.81 | 9 | 3 | 6 | 13 | 0.810 | 1.026 | 1.264 | 1.265 | 1.556 | 1.227 |
| Q6DRA6 | H2B2D | 27.44 | 10 | 3 | 6 | 49 | 1.095 | 1.026 | 0.929 | 1.182 | 1.076 | 1.184 |
| Q9Y2Z4 | SYYM | 6.71 | 1 | 3 | 3 | 5 | 0.962 | 1.026 | 1.044 | 1.014 | 1.104 | 1.151 |
| Q14980 | NUMA1 | 4.96 | 5 | 8 | 8 | 8 | 1.010 | 1.026 | 1.014 | 1.069 | 1.050 | 1.020 |
| Q8N1G4 | LRC47 | 3.77 | 1 | 2 | 2 | 2 | 0.924 | 1.025 | 1.107 | 0.988 | 1.066 | 0.959 |
| Q9Y6U3 | ADSV | 6.57 | 1 | 5 | 5 | 9 | 1.006 | 1.025 | 1.017 | 1.096 | 1.094 | 1.075 |
| B1AH89 | B1AH89 | 13.21 | 3 | 6 | 6 | 17 | 0.981 | 1.025 | 1.071 | 0.938 | 1.002 | 0.944 |
| Q96CG1 | Q96CG1 | 18.81 | 5 | 6 | 6 | 12 | 0.933 | 1.025 | 1.045 | 1.013 | 0.933 | 1.020 |
| B4DUM2 | B4DUM2 | 18.42 | 3 | 4 | 4 | 7 | 0.819 | 1.025 | 1.158 | 1.006 | 1.085 | 0.890 |
| P21796 | VDAC1 | 21.55 | 3 | 5 | 6 | 33 | 1.070 | 1.025 | 0.963 | 1.114 | 1.049 | 1.101 |
| B4DZ20 | B4DZ20 | 3.76 | 10 | 2 | 2 | 2 | 0.733 | 1.024 | 1.395 | 0.945 | 1.286 | 0.918 |
| P38159 | HNRPG | 31.46 | 6 | 12 | 12 | 62 | 1.010 | 1.024 | 1.018 | 1.018 | 0.986 | 0.982 |
| P52565 | GDIR1 | 28.92 | 2 | 5 | 5 | 11 | 1.029 | 1.024 | 0.972 | 1.038 | 0.970 | 1.011 |
| P35579 | MYH9 | 26.63 | 11 | 40 | 49 | 143 | 1.026 | 1.024 | 1.029 | 1.035 | 1.038 | 0.976 |
| Q9BYC5 | FUT8 | 6.78 | 4 | 3 | 3 | 3 | 1.135 | 1.023 | 0.900 | 0.782 | 1.081 | 1.242 |
| P41252 | SYIC | 13.95 | 8 | 16 | 16 | 42 | 1.003 | 1.023 | 1.017 | 0.946 | 0.930 | 0.958 |
| E7ET15 | E7ET15 | 8.66 | 6 | 7 | 7 | 19 | 1.013 | 1.022 | 1.000 | 1.078 | 1.041 | 1.028 |
| B4DW94 | B4DW94 | 29.20 | 24 | 5 | 5 | 38 | 0.994 | 1.021 | 1.019 | 0.965 | 0.959 | 0.941 |
| P09382 | LEG1 | 31.11 | 1 | 4 | 4 | 18 | 0.987 | 1.021 | 1.032 | 0.901 | 0.891 | 0.935 |
| P55081 | MFAP1 | 6.38 | 1 | 3 | 3 | 3 | 1.083 | 1.021 | 1.006 | 1.002 | 1.003 | 0.993 |
| P35232 | PHB | 46.69 | 7 | 11 | 11 | 47 | 1.015 | 1.020 | 0.990 | 0.945 | 0.944 | 0.931 |
| P53007 | TXTP | 5.47 | 2 | 2 | 2 | 4 | 1.212 | 1.020 | 0.845 | 1.107 | 0.909 | 1.202 |
| B4DXZ6 | B4DXZ6 | 10.36 | 7 | 5 | 5 | 8 | 1.084 | 1.020 | 0.904 | 1.097 | 0.927 | 1.071 |
| O60664 | PLIN3 | 23.04 | 1 | 7 | 7 | 29 | 0.962 | 1.020 | 1.039 | 0.984 | 1.034 | 0.982 |
| O14980 | XPO1 | 14.75 | 6 | 12 | 12 | 79 | 1.008 | 1.019 | 1.070 | 1.033 | 1.040 | 1.023 |
| P61353 | RL27 | 20.59 | 1 | 3 | 3 | 14 | 1.217 | 1.019 | 0.835 | 1.319 | 1.100 | 1.249 |
| P46940 | IQGA1 | 9.29 | 2 | 13 | 13 | 18 | 0.991 | 1.019 | 1.070 | 1.051 | 1.111 | 0.986 |
| B7Z1K2 | B7Z1K2 | 7.35 | 4 | 2 | 2 | 2 | 0.994 | 1.019 | 1.023 | 0.935 | 0.938 | 0.914 |
| Q9Y3Q8 | T22D4 | 5.57 | 5 | 2 | 2 | 2 | 0.811 | 1.019 | 1.070 | 0.888 | 1.091 | 1.243 |
| F2Z3H3 | F2Z3H3 | 14.29 | 3 | 2 | 4 | 12 | 1.027 | 1.018 | 1.039 | 1.030 | 1.054 | 1.056 |
| P38919 | IF4A3 | 23.84 | 3 | 6 | 8 | 24 | 1.091 | 1.018 | 0.957 | 1.019 | 0.963 | 1.003 |
| Q99798 | ACON | 7.82 | 5 | 4 | 4 | 5 | 0.866 | 1.017 | 1.123 | 0.931 | 0.953 | 0.852 |
| P26639 | SYTC | 10.37 | 10 | 8 | 8 | 20 | 1.067 | 1.017 | 1.006 | 0.993 | 0.919 | 0.986 |
| P46109 | CRKL | 31.02 | 1 | 7 | 7 | 36 | 0.990 | 1.017 | 0.984 | 1.050 | 1.016 | 0.976 |
| P22234 | PUR6 | 24.47 | 3 | 9 | 9 | 43 | 0.924 | 1.017 | 1.009 | 0.973 | 0.986 | 0.974 |
| P40926 | MDHM | 35.80 | 3 | 11 | 11 | 111 | 1.025 | 1.017 | 0.985 | 1.021 | 1.007 | 1.009 |
| Q9P0J0 | NDUAD | 26.39 | 2 | 3 | 3 | 9 | 0.915 | 1.017 | 1.110 | 0.985 | 1.073 | 0.952 |
| D6RAF8 | D6RAF8 | 32.58 | 7 | 6 | 7 | 76 | 1.014 | 1.017 | 0.995 | 1.010 | 1.010 | 0.993 |
| P30405 | PPIF | 14.49 | 2 | 2 | 4 | 6 | 0.900 | 1.016 | 1.128 | 1.023 | 1.134 | 1.002 |
| Q06124 | PTN11 | 5.19 | 1 | 3 | 3 | 4 | 0.960 | 1.016 | 1.084 | 0.954 | 1.028 | 0.982 |
| Q14566 | MCM6 | 21.07 | 1 | 16 | 16 | 51 | 0.978 | 1.016 | 1.034 | 0.986 | 1.036 | 0.979 |
| Q9Y383 | LC7L2 | 22.96 | 8 | 6 | 9 | 28 | 0.997 | 1.016 | 0.979 | 0.999 | 1.012 | 0.983 |
| B4DUR9 | B4DUR9 | 27.08 | 4 | 6 | 6 | 11 | 1.098 | 1.015 | 0.906 | 0.972 | 0.874 | 0.981 |
| C9J9K3 | C9J9K3 | 36.74 | 4 | 7 | 8 | 47 | 0.985 | 1.015 | 0.969 | 0.988 | 0.974 | 1.011 |
| P34932 | HSP74 | 27.26 | 3 | 17 | 19 | 47 | 0.997 | 1.015 | 0.999 | 0.971 | 0.960 | 0.985 |
| Q9Y2S7 | PDIP2 | 7.07 | 1 | 2 | 2 | 2 | 0.780 | 1.015 | 1.299 | 0.882 | 1.127 | 0.865 |
| F5GYQ4 | F5GYQ4 | 24.29 | 7 | 11 | 11 | 19 | 0.953 | 1.015 | 1.059 | 1.083 | 1.180 | 1.068 |
| Q7L014 | DDX46 | 9.80 | 3 | 11 | 11 | 23 | 0.996 | 1.015 | 1.054 | 0.932 | 0.906 | 0.955 |
| P62888 | RL30 | 29.57 | 3 | 3 | 4 | 9 | 1.037 | 1.015 | 0.897 | 0.986 | 0.991 | 1.058 |
| B4DIT7 | B4DIT7 | 4.29 | 3 | 2 | 2 | 2 | 0.819 | 1.015 | 1.236 | 1.004 | 1.221 | 0.985 |
| Q2TAY7 | SMU1 | 3.90 | 1 | 2 | 2 | 11 | 0.901 | 1.015 | 0.975 | 0.900 | 0.907 | 1.048 |
| F5GZI1 | F5GZI1 | 5.73 | 5 | 5 | 5 | 6 | 0.939 | 1.014 | 1.014 | 1.121 | 1.111 | 1.043 |
| A6NJH9 | A6NJH9 | 13.39 | 3 | 2 | 2 | 3 | 1.077 | 1.014 | 0.917 | 1.050 | 0.972 | 1.067 |
| P55072 | TERA | 37.22 | 3 | 26 | 26 | 109 | 0.991 | 1.014 | 0.986 | 1.003 | 1.010 | 1.006 |
| P23246 | SFPQ | 27.58 | 1 | 17 | 18 | 68 | 1.005 | 1.014 | 1.000 | 0.995 | 0.987 | 1.018 |
| F5H0G6 | F5H0G6 | 5.23 | 2 | 2 | 2 | 3 | 1.002 | 1.014 | 1.271 | 1.173 | 1.112 | 1.152 |
| P46821 | MAP1B | 1.99 | 1 | 4 | 4 | 4 | 1.016 | 1.013 | 0.991 | 0.980 | 0.896 | 0.968 |
| P32119 | PRDX2 | 36.36 | 3 | 8 | 9 | 55 | 0.984 | 1.013 | 1.030 | 1.034 | 1.047 | 1.011 |
| E7EVX8 | E7EVX8 | 7.10 | 6 | 3 | 3 | 5 | 1.045 | 1.012 | 1.052 | 0.972 | 0.944 | 0.954 |
| P13796 | PLSL | 44.18 | 10 | 21 | 21 | 61 | 0.978 | 1.012 | 1.049 | 1.015 | 1.039 | 1.022 |
| P23526 | SAHH | 25.46 | 2 | 11 | 11 | 41 | 1.024 | 1.012 | 0.964 | 0.955 | 0.928 | 0.982 |
| P04083 | ANXA1 | 25.43 | 3 | 9 | 9 | 44 | 0.974 | 1.011 | 1.022 | 1.128 | 1.100 | 1.074 |
| E7EPK1 | E7EPK1 | 18.35 | 12 | 5 | 7 | 8 | 0.993 | 1.011 | 1.005 | 1.125 | 1.084 | 1.037 |
| E9PGR0 | E9PGR0 | 12.30 | 7 | 3 | 3 | 3 | 1.064 | 1.011 | 1.025 | 1.084 | 0.971 | 1.138 |
| E5RH50 | E5RH50 | 3.27 | 2 | 2 | 2 | 3 | 1.050 | 1.011 | 0.979 | 0.975 | 0.993 | 1.007 |
| Q9BV20 | MTNA | 5.69 | 1 | 2 | 2 | 2 | 1.036 | 1.011 | 0.974 | 1.126 | 1.084 | 1.109 |
| P61247 | RS3A | 42.80 | 11 | 12 | 12 | 20 | 1.190 | 1.011 | 0.851 | 1.121 | 0.987 | 1.130 |
| E9PFH8 | E9PFH8 | 23.27 | 6 | 8 | 8 | 35 | 0.986 | 1.010 | 1.064 | 1.049 | 1.066 | 1.016 |
| B4DJ10 | B4DJ10 | 10.96 | 6 | 9 | 9 | 38 | 0.957 | 1.010 | 1.009 | 0.977 | 1.074 | 1.016 |
| Q15008 | PSMD6 | 9.00 | 6 | 3 | 3 | 13 | 0.881 | 1.010 | 1.056 | 0.994 | 1.109 | 0.938 |
| Q7Z2W4 | ZCCHV | 2.77 | 3 | 2 | 2 | 2 | 0.911 | 1.010 | 1.106 | 0.975 | 1.067 | 0.961 |
| B5MC59 | B5MC59 | 32.93 | 2 | 2 | 2 | 10 | 0.952 | 1.009 | 1.024 | 1.033 | 1.078 | 1.109 |
| Q14677 | EPN4 | 3.20 | 2 | 2 | 2 | 2 | 1.204 | 1.009 | 0.836 | 0.922 | 0.763 | 0.909 |
| F5HGG6 | F5HGG6 | 5.16 | 2 | 2 | 2 | 4 | 1.024 | 1.009 | 0.967 | 1.096 | 1.008 | 1.026 |
| P12270 | TPR | 5.54 | 1 | 10 | 10 | 13 | 1.013 | 1.009 | 0.971 | 1.021 | 0.975 | 1.006 |
| Q92878 | RAD50 | 2.13 | 3 | 3 | 3 | 3 | 1.314 | 1.009 | 0.766 | 1.067 | 0.809 | 1.053 |
| D3YTB1 | D3YTB1 | 11.28 | 3 | 2 | 2 | 4 | 1.508 | 1.008 | 0.591 | 1.467 | 0.821 | 1.448 |
| Q92616 | GCN1L | 7.53 | 1 | 13 | 13 | 26 | 0.965 | 1.008 | 1.008 | 0.881 | 0.979 | 0.936 |
| C9IZ01 | C9IZ01 | 5.58 | 3 | 3 | 3 | 3 | 1.144 | 1.008 | 0.972 | 1.033 | 0.900 | 0.919 |
| Q15637 | SF01 | 10.49 | 6 | 5 | 5 | 22 | 1.042 | 1.008 | 0.912 | 0.932 | 0.965 | 0.995 |
| G3V4W0 | G3V4W0 | 38.17 | 20 | 10 | 10 | 95 | 1.003 | 1.008 | 1.000 | 1.066 | 1.072 | 1.071 |
| P06493 | CDK1 | 28.62 | 33 | 7 | 8 | 24 | 0.944 | 1.008 | 1.111 | 0.925 | 1.007 | 0.970 |
| G3XAI4 | G3XAI4 | 18.52 | 4 | 15 | 15 | 31 | 0.997 | 1.008 | 1.029 | 0.985 | 0.965 | 0.909 |
| Q7Z784 | Q7Z784 | 7.99 | 2 | 4 | 4 | 9 | 1.057 | 1.007 | 1.034 | 1.009 | 1.106 | 0.954 |
| Q9UBT2 | SAE2 | 9.69 | 3 | 6 | 6 | 6 | 0.983 | 1.007 | 0.974 | 0.982 | 1.000 | 0.986 |
| F8VXJ7 | F8VXJ7 | 19.05 | 6 | 2 | 2 | 2 | 1.053 | 1.007 | 0.955 | 1.078 | 1.021 | 1.065 |
| E9PH29 | E9PH29 | 16.81 | 2 | 4 | 4 | 22 | 1.020 | 1.007 | 1.021 | 1.032 | 1.025 | 1.061 |
| O75874 | IDHC | 18.36 | 4 | 7 | 7 | 35 | 0.960 | 1.007 | 1.044 | 1.029 | 1.072 | 1.001 |
| P25705 | ATPA | 51.72 | 2 | 25 | 26 | 138 | 0.978 | 1.007 | 0.992 | 0.997 | 1.020 | 1.013 |
| Q86XP3 | DDX42 | 8.10 | 1 | 6 | 6 | 11 | 1.003 | 1.006 | 1.004 | 0.984 | 0.970 | 0.899 |
| Q9NRN7 | ADPPT | 10.03 | 4 | 3 | 3 | 5 | 0.958 | 1.006 | 0.997 | 0.883 | 0.913 | 0.929 |
| E7EWT1 | E7EWT1 | 16.47 | 2 | 6 | 6 | 16 | 1.044 | 1.006 | 0.966 | 0.991 | 0.976 | 1.053 |
| Q92688 | AN32B | 17.13 | 2 | 4 | 6 | 36 | 0.978 | 1.006 | 1.059 | 0.989 | 1.037 | 0.984 |
| P07355 | ANXA2 | 44.25 | 3 | 16 | 16 | 62 | 0.999 | 1.006 | 0.999 | 1.047 | 1.044 | 1.022 |
| F5H569 | F5H569 | 2.39 | 5 | 2 | 2 | 3 | 1.130 | 1.006 | 0.846 | 1.193 | 1.155 | 1.210 |
| Q9UHX1 | PUF60 | 20.75 | 5 | 8 | 8 | 9 | 0.995 | 1.005 | 0.897 | 0.940 | 0.931 | 1.035 |
| Q14152 | EIF3A | 18.74 | 2 | 24 | 24 | 62 | 1.011 | 1.005 | 0.958 | 0.982 | 0.960 | 0.941 |
| P04040 | CATA | 15.56 | 1 | 6 | 6 | 17 | 0.956 | 1.005 | 1.040 | 0.954 | 1.161 | 0.951 |
| Q15149 | PLEC | 6.00 | 4 | 24 | 25 | 45 | 0.989 | 1.004 | 0.975 | 1.035 | 1.024 | 0.997 |
| P63151 | 2ABA | 17.45 | 11 | 7 | 7 | 17 | 0.931 | 1.004 | 1.102 | 0.882 | 0.966 | 0.860 |
| O00429 | DNM1L | 11.96 | 7 | 6 | 6 | 14 | 1.000 | 1.004 | 1.022 | 0.968 | 0.978 | 0.950 |
| F8VWB4 | F8VWB4 | 4.24 | 7 | 3 | 3 | 4 | 1.101 | 1.004 | 0.947 | 1.063 | 0.963 | 1.054 |
| P62081 | RS7 | 26.29 | 2 | 6 | 6 | 27 | 1.071 | 1.004 | 0.912 | 1.045 | 1.008 | 1.082 |
| P63244 | GBLP | 23.97 | 16 | 8 | 8 | 29 | 1.076 | 1.004 | 0.939 | 0.986 | 0.908 | 0.979 |
| O14776 | TCRG1 | 3.83 | 2 | 4 | 4 | 7 | 0.964 | 1.004 | 1.037 | 0.836 | 0.927 | 0.922 |
| P54577 | SYYC | 15.34 | 1 | 9 | 9 | 23 | 1.036 | 1.004 | 0.987 | 0.997 | 0.929 | 1.016 |
| Q07157 | ZO1 | 3.72 | 2 | 4 | 4 | 8 | 0.867 | 1.003 | 1.334 | 1.026 | 1.158 | 0.991 |
| Q96HE7 | ERO1A | 7.05 | 3 | 3 | 3 | 4 | 0.966 | 1.003 | 0.832 | 0.934 | 0.823 | 0.986 |
| P61026 | RAB10 | 22.50 | 4 | 2 | 4 | 20 | 1.110 | 1.003 | 0.911 | 1.040 | 1.025 | 0.998 |
| P41250 | SYG | 12.18 | 1 | 8 | 8 | 28 | 1.006 | 1.003 | 0.958 | 0.967 | 1.024 | 0.985 |
| P48643 | TCPE | 31.24 | 7 | 14 | 14 | 146 | 0.996 | 1.003 | 1.012 | 0.964 | 0.958 | 0.959 |
| P51991 | ROA3 | 25.93 | 3 | 9 | 12 | 32 | 1.080 | 1.003 | 0.937 | 0.999 | 0.955 | 1.009 |
| P00492 | HPRT | 26.61 | 1 | 5 | 5 | 20 | 1.018 | 1.003 | 0.939 | 0.954 | 0.956 | 0.903 |
| Q4VBZ6 | Q4VBZ6 | 22.96 | 17 | 5 | 5 | 24 | 0.960 | 1.003 | 1.044 | 1.056 | 1.115 | 1.026 |
| P30041 | PRDX6 | 31.25 | 1 | 6 | 6 | 26 | 1.042 | 1.002 | 0.967 | 1.101 | 1.013 | 1.039 |
| B7Z382 | B7Z382 | 9.96 | 4 | 3 | 3 | 11 | 0.960 | 1.002 | 1.118 | 1.021 | 1.135 | 1.035 |
| P46783 | RS10 | 46.67 | 3 | 7 | 8 | 103 | 1.068 | 1.002 | 0.946 | 1.053 | 0.985 | 1.037 |
| E7EMI4 | E7EMI4 | 4.01 | 5 | 2 | 3 | 5 | 0.944 | 1.002 | 1.029 | 1.005 | 0.944 | 0.932 |
| P19105 | ML12A | 37.43 | 4 | 7 | 7 | 28 | 0.986 | 1.002 | 0.979 | 1.006 | 1.006 | 1.022 |
| P33992 | MCM5 | 19.75 | 7 | 12 | 12 | 23 | 1.044 | 1.001 | 0.947 | 1.092 | 1.018 | 1.037 |
| P78371 | TCPB | 53.27 | 4 | 21 | 21 | 117 | 0.988 | 1.001 | 1.031 | 0.999 | 0.972 | 0.957 |
| Q9BSL1 | UBAC1 | 9.14 | 1 | 3 | 3 | 5 | 0.887 | 1.000 | 1.126 | 1.016 | 1.107 | 0.979 |
| P49770 | EI2BB | 8.83 | 1 | 2 | 2 | 2 | 0.944 | 1.000 | 1.058 | 0.975 | 1.029 | 0.970 |
| P09874 | PARP1 | 15.29 | 4 | 13 | 13 | 33 | 1.033 | 1.000 | 0.976 | 1.057 | 0.996 | 1.055 |
| P55010 | IF5 | 20.19 | 1 | 8 | 8 | 43 | 0.961 | 1.000 | 1.004 | 1.001 | 1.071 | 1.045 |
| F5GXX7 | F5GXX7 | 26.32 | 5 | 6 | 6 | 7 | 0.960 | 1.000 | 1.047 | 1.172 | 1.180 | 1.166 |
| P42167 | LAP2B | 7.71 | 2 | 3 | 3 | 20 | 0.910 | 0.999 | 1.044 | 0.881 | 0.966 | 0.957 |
| E9PD53 | E9PD53 | 4.67 | 9 | 5 | 5 | 5 | 0.908 | 0.999 | 1.168 | 1.045 | 1.067 | 0.837 |
| O60885 | BRD4 | 2.13 | 5 | 3 | 3 | 5 | 1.014 | 0.999 | 0.879 | 0.953 | 0.837 | 1.024 |
| O43809 | CPSF5 | 7.49 | 1 | 2 | 2 | 3 | 0.931 | 0.999 | 0.915 | 1.498 | 1.604 | 2.438 |
| Q00688 | FKBP3 | 13.39 | 2 | 3 | 3 | 8 | 1.154 | 0.999 | 0.988 | 1.010 | 0.935 | 1.000 |
| Q8N1G2 | MTR1 | 2.87 | 2 | 2 | 2 | 2 | 0.805 | 0.999 | 1.238 | 0.912 | 1.128 | 0.909 |
| Q09028 | RBBP4 | 19.29 | 8 | 2 | 10 | 33 | 1.084 | 0.998 | 0.920 | 1.023 | 0.941 | 1.020 |
| P78344 | IF4G2 | 4.85 | 3 | 5 | 5 | 24 | 1.062 | 0.998 | 0.936 | 1.021 | 0.980 | 1.032 |
| E9PE82 | E9PE82 | 5.15 | 2 | 2 | 2 | 8 | 1.078 | 0.998 | 0.908 | 1.210 | 1.250 | 0.891 |
| P12814 | ACTN1 | 12.67 | 10 | 3 | 11 | 87 | 1.088 | 0.997 | 1.047 | 1.065 | 0.976 | 1.039 |
| Q16543 | CDC37 | 13.23 | 1 | 5 | 5 | 6 | 1.000 | 0.997 | 1.044 | 1.105 | 1.037 | 1.029 |
| F8W1R7 | F8W1R7 | 46.90 | 12 | 7 | 7 | 32 | 1.015 | 0.997 | 0.992 | 1.019 | 1.028 | 1.020 |
| P16401 | H15 | 12.39 | 1 | 2 | 3 | 30 | 1.042 | 0.997 | 0.835 | 1.259 | 1.223 | 1.272 |
| F8W851 | F8W851 | 8.80 | 11 | 2 | 2 | 2 | 0.939 | 0.997 | 1.060 | 0.848 | 0.900 | 0.846 |
| A8MX94 | A8MX94 | 26.44 | 2 | 3 | 3 | 16 | 0.982 | 0.997 | 1.012 | 1.016 | 1.047 | 1.036 |
| P62258 | 1433E | 37.25 | 1 | 6 | 9 | 83 | 1.083 | 0.997 | 0.974 | 1.061 | 0.995 | 1.018 |
| P07737 | PROF1 | 55.00 | 1 | 8 | 8 | 106 | 1.033 | 0.997 | 0.969 | 1.040 | 1.011 | 1.047 |
| P06733 | ENOA | 51.61 | 6 | 20 | 21 | 446 | 1.023 | 0.996 | 0.968 | 1.014 | 0.991 | 1.010 |
| Q9UN86 | G3BP2 | 8.71 | 5 | 3 | 4 | 10 | 0.985 | 0.996 | 0.936 | 0.983 | 0.927 | 0.908 |
| P07814 | SYEP | 8.20 | 5 | 12 | 12 | 14 | 1.053 | 0.996 | 0.944 | 0.996 | 0.971 | 0.970 |
| O15355 | PPM1G | 4.76 | 2 | 2 | 2 | 23 | 0.963 | 0.996 | 0.995 | 0.899 | 1.010 | 0.972 |
| A3KFL1 | A3KFL1 | 13.00 | 6 | 2 | 2 | 2 | 1.095 | 0.996 | 0.907 | 1.056 | 0.961 | 1.056 |
| O95202 | LETM1 | 5.28 | 2 | 3 | 3 | 3 | 1.000 | 0.995 | 0.941 | 1.104 | 1.014 | 1.023 |
| C9J938 | C9J938 | 36.49 | 15 | 3 | 4 | 6 | 0.993 | 0.995 | 0.969 | 1.049 | 1.070 | 1.009 |
| E9PM69 | E9PM69 | 38.04 | 10 | 11 | 11 | 21 | 0.922 | 0.995 | 1.003 | 1.005 | 0.986 | 0.991 |
| P50502 | F10A1 | 23.31 | 7 | 8 | 8 | 48 | 0.944 | 0.995 | 1.106 | 1.032 | 1.121 | 1.008 |
| Q14683 | SMC1A | 6.00 | 2 | 7 | 7 | 10 | 1.075 | 0.995 | 0.916 | 0.920 | 0.871 | 0.904 |
| P62829 | RL23 | 23.57 | 3 | 3 | 3 | 27 | 1.045 | 0.995 | 0.899 | 0.979 | 0.934 | 1.019 |
| P22087 | FBRL | 5.61 | 2 | 2 | 2 | 17 | 1.136 | 0.995 | 0.903 | 1.404 | 1.205 | 1.398 |
| P14618 | KPYM | 46.33 | 5 | 20 | 22 | 161 | 0.987 | 0.994 | 1.003 | 0.968 | 0.984 | 0.977 |
| P60866 | RS20 | 19.33 | 5 | 2 | 2 | 16 | 1.037 | 0.994 | 0.927 | 0.938 | 0.971 | 1.016 |
| O43707 | ACTN4 | 27.66 | 12 | 15 | 23 | 121 | 0.973 | 0.994 | 0.983 | 0.973 | 0.994 | 0.979 |
| Q6P2Q9 | PRP8 | 8.22 | 4 | 18 | 18 | 23 | 1.145 | 0.994 | 0.903 | 1.045 | 0.947 | 1.045 |
| F8W720 | F8W720 | 9.26 | 3 | 5 | 5 | 21 | 1.034 | 0.994 | 0.981 | 0.929 | 0.948 | 0.947 |
| E7ESM6 | E7ESM6 | 22.00 | 2 | 17 | 17 | 49 | 0.956 | 0.994 | 1.019 | 1.000 | 1.074 | 1.027 |
| B4DGD5 | B4DGD5 | 9.17 | 5 | 4 | 4 | 4 | 1.002 | 0.994 | 1.067 | 1.047 | 1.066 | 1.003 |
| B3KY27 | B3KY27 | 8.00 | 6 | 2 | 2 | 3 | 0.853 | 0.993 | 1.265 | 0.875 | 0.951 | 0.748 |
| P53999 | TCP4 | 37.01 | 2 | 4 | 4 | 11 | 1.051 | 0.993 | 0.988 | 1.147 | 1.073 | 1.079 |
| P36542 | ATPG | 19.80 | 2 | 6 | 6 | 16 | 1.011 | 0.993 | 0.995 | 1.046 | 0.935 | 0.962 |
| C9JFV5 | C9JFV5 | 18.98 | 3 | 11 | 12 | 30 | 0.991 | 0.993 | 1.001 | 0.943 | 0.981 | 0.973 |
| Q14011 | CIRBP | 20.93 | 3 | 3 | 3 | 3 | 0.998 | 0.993 | 1.011 | 1.223 | 1.187 | 1.292 |
| O00232 | PSD12 | 25.00 | 2 | 9 | 9 | 15 | 0.928 | 0.992 | 1.029 | 0.867 | 0.961 | 0.930 |
| F5GX39 | F5GX39 | 18.97 | 5 | 2 | 2 | 4 | 0.969 | 0.992 | 0.931 | 0.967 | 0.929 | 0.953 |
| Q8WVC2 | Q8WVC2 | 22.22 | 2 | 3 | 3 | 10 | 1.071 | 0.992 | 0.944 | 0.970 | 0.903 | 0.993 |
| O15144 | ARPC2 | 18.00 | 3 | 4 | 4 | 11 | 0.978 | 0.992 | 1.013 | 0.983 | 1.093 | 0.975 |
| O00299 | CLIC1 | 31.95 | 1 | 7 | 7 | 54 | 0.995 | 0.992 | 0.981 | 0.957 | 0.988 | 0.987 |
| E9PLK3 | E9PLK3 | 17.16 | 12 | 13 | 13 | 22 | 0.996 | 0.991 | 0.976 | 1.035 | 1.104 | 0.990 |
| O00567 | NOP56 | 8.92 | 3 | 4 | 4 | 7 | 1.136 | 0.991 | 1.015 | 1.014 | 0.985 | 1.056 |
| Q14690 | RRP5 | 2.03 | 2 | 3 | 3 | 6 | 1.159 | 0.991 | 0.831 | 1.117 | 1.004 | 1.014 |
| P12004 | PCNA | 23.75 | 1 | 6 | 6 | 28 | 1.041 | 0.991 | 0.966 | 0.986 | 0.991 | 1.017 |
| Q14697 | GANAB | 20.87 | 4 | 15 | 15 | 34 | 1.013 | 0.991 | 0.985 | 0.962 | 0.994 | 0.927 |
| P30040 | ERP29 | 16.09 | 4 | 4 | 4 | 37 | 0.910 | 0.991 | 0.958 | 0.931 | 0.969 | 0.950 |
| B4DR64 | B4DR64 | 11.67 | 3 | 3 | 3 | 11 | 0.801 | 0.991 | 1.078 | 0.950 | 1.117 | 1.001 |
| F5H3V0 | F5H3V0 | 24.88 | 3 | 16 | 16 | 59 | 0.994 | 0.991 | 1.009 | 1.031 | 1.012 | 0.986 |
| Q9Y230 | RUVB2 | 21.81 | 2 | 9 | 9 | 22 | 0.977 | 0.990 | 0.982 | 0.995 | 0.972 | 0.988 |
| O95573 | ACSL3 | 4.03 | 2 | 3 | 3 | 5 | 1.088 | 0.990 | 0.889 | 1.053 | 1.005 | 1.127 |
| E9PK91 | E9PK91 | 4.82 | 6 | 5 | 5 | 5 | 1.017 | 0.990 | 0.961 | 1.097 | 1.037 | 0.989 |
| O43390 | HNRPR | 20.06 | 4 | 7 | 12 | 35 | 1.001 | 0.990 | 0.980 | 0.997 | 1.004 | 1.004 |
| P62753 | RS6 | 13.25 | 3 | 3 | 3 | 8 | 1.516 | 0.990 | 0.665 | 1.249 | 0.811 | 1.274 |
| E9PC52 | E9PC52 | 18.03 | 7 | 2 | 9 | 32 | 1.002 | 0.989 | 0.985 | 0.957 | 0.951 | 0.962 |
| B0V043 | B0V043 | 10.76 | 7 | 11 | 11 | 28 | 1.011 | 0.989 | 0.933 | 0.974 | 0.923 | 0.958 |
| F8VV40 | F8VV40 | 9.03 | 2 | 5 | 5 | 20 | 0.981 | 0.989 | 1.033 | 1.090 | 0.982 | 0.911 |
| Q07955 | SRSF1 | 32.26 | 1 | 9 | 9 | 33 | 1.001 | 0.989 | 0.988 | 1.008 | 0.994 | 1.012 |
| E9PFS5 | E9PFS5 | 4.45 | 15 | 5 | 5 | 5 | 0.899 | 0.989 | 1.161 | 1.027 | 1.174 | 0.964 |
| O75533 | SF3B1 | 11.43 | 4 | 9 | 9 | 17 | 0.980 | 0.988 | 1.028 | 0.970 | 0.956 | 0.936 |
| O76003 | GLRX3 | 30.75 | 1 | 9 | 9 | 30 | 1.050 | 0.988 | 0.920 | 1.074 | 1.047 | 0.999 |
| E9PL01 | E9PL01 | 22.29 | 4 | 3 | 3 | 5 | 0.845 | 0.988 | 1.283 | 1.099 | 1.269 | 1.083 |
| Q96HS1 | PGAM5 | 6.57 | 1 | 2 | 2 | 2 | 0.989 | 0.988 | 0.997 | 0.860 | 0.867 | 0.867 |
| P62993 | GRB2 | 34.56 | 1 | 8 | 8 | 14 | 1.003 | 0.987 | 0.990 | 0.959 | 0.931 | 0.975 |
| Q9UQE7 | SMC3 | 5.67 | 1 | 6 | 6 | 7 | 1.011 | 0.987 | 1.021 | 1.106 | 0.998 | 0.988 |
| B7Z9F1 | B7Z9F1 | 21.95 | 20 | 9 | 9 | 30 | 1.017 | 0.987 | 0.932 | 0.951 | 0.909 | 0.956 |
| P12956 | XRCC6 | 40.23 | 5 | 19 | 19 | 64 | 0.921 | 0.987 | 1.047 | 0.993 | 1.074 | 0.992 |
| P17987 | TCPA | 33.09 | 11 | 14 | 14 | 74 | 1.021 | 0.987 | 0.982 | 0.985 | 0.986 | 1.018 |
| E9PID8 | E9PID8 | 7.99 | 5 | 3 | 3 | 3 | 1.138 | 0.987 | 0.806 | 1.043 | 0.850 | 0.980 |
| D6RD69 | D6RD69 | 13.53 | 8 | 2 | 2 | 5 | 0.960 | 0.987 | 0.943 | 1.108 | 1.058 | 1.118 |
| P27824 | CALX | 18.58 | 12 | 10 | 10 | 27 | 0.932 | 0.986 | 1.015 | 0.924 | 1.045 | 1.026 |
| F8W726 | F8W726 | 3.53 | 5 | 3 | 3 | 20 | 0.857 | 0.986 | 1.135 | 0.894 | 1.076 | 0.911 |
| P68402 | PA1B2 | 12.23 | 4 | 2 | 2 | 25 | 0.868 | 0.986 | 1.119 | 0.882 | 1.004 | 0.901 |
| Q9BXP5 | SRRT | 6.85 | 2 | 6 | 6 | 8 | 1.029 | 0.986 | 0.984 | 0.984 | 1.058 | 1.015 |
| Q01082 | SPTB2 | 8.04 | 4 | 17 | 17 | 26 | 0.907 | 0.986 | 0.968 | 0.895 | 0.943 | 1.029 |
| A8K3Z3 | A8K3Z3 | 32.91 | 2 | 10 | 10 | 24 | 0.967 | 0.986 | 1.000 | 1.033 | 1.013 | 1.029 |
| P30046 | DOPD | 22.88 | 4 | 3 | 3 | 23 | 0.976 | 0.986 | 1.004 | 0.955 | 1.000 | 0.988 |
| P56192 | SYMC | 9.11 | 3 | 7 | 7 | 11 | 0.951 | 0.986 | 0.980 | 0.921 | 0.949 | 0.957 |
| B1ANR0 | B1ANR0 | 12.85 | 4 | 5 | 7 | 19 | 0.961 | 0.985 | 1.025 | 1.056 | 1.169 | 1.100 |
| Q01130 | SRSF2 | 19.00 | 4 | 5 | 5 | 17 | 1.060 | 0.985 | 0.900 | 1.112 | 1.004 | 1.007 |
| O60506 | HNRPQ | 25.04 | 2 | 10 | 15 | 53 | 0.931 | 0.985 | 1.018 | 0.931 | 0.995 | 1.000 |
| B7Z1N6 | B7Z1N6 | 28.92 | 8 | 5 | 7 | 21 | 0.931 | 0.984 | 1.082 | 0.953 | 1.145 | 0.978 |
| P60891 | PRPS1 | 18.55 | 8 | 6 | 6 | 10 | 1.012 | 0.984 | 0.996 | 0.866 | 0.867 | 0.902 |
| Q13177 | PAK2 | 9.35 | 3 | 5 | 5 | 7 | 1.072 | 0.984 | 0.872 | 1.035 | 0.824 | 1.046 |
| P35998 | PRS7 | 24.25 | 3 | 7 | 8 | 24 | 0.972 | 0.984 | 0.992 | 1.009 | 1.005 | 0.974 |
| B4DHT4 | B4DHT4 | 9.52 | 4 | 3 | 3 | 3 | 1.082 | 0.984 | 0.923 | 0.901 | 0.699 | 0.938 |
| Q06210 | GFPT1 | 11.02 | 2 | 6 | 6 | 10 | 0.909 | 0.984 | 0.949 | 0.957 | 1.143 | 0.999 |
| E7ERW2 | E7ERW2 | 10.85 | 3 | 4 | 4 | 6 | 1.078 | 0.984 | 0.932 | 1.077 | 1.000 | 1.038 |
| P13073 | COX41 | 23.67 | 1 | 4 | 4 | 6 | 1.040 | 0.984 | 0.974 | 1.052 | 1.054 | 1.079 |
| P31942 | HNRH3 | 19.65 | 2 | 6 | 6 | 11 | 1.033 | 0.984 | 0.968 | 1.029 | 1.072 | 1.006 |
| E7EMJ6 | E7EMJ6 | 11.68 | 2 | 3 | 3 | 7 | 1.001 | 0.984 | 0.989 | 1.008 | 1.000 | 1.025 |
| P60709 | ACTB | 52.00 | 21 | 5 | 19 | 469 | 1.010 | 0.983 | 0.980 | 0.990 | 0.980 | 0.991 |
| P48444 | COPD | 14.29 | 5 | 6 | 6 | 17 | 0.956 | 0.983 | 1.084 | 0.974 | 0.999 | 1.030 |
| Q13247 | SRSF6 | 8.72 | 4 | 2 | 3 | 4 | 1.042 | 0.983 | 0.941 | 1.015 | 0.970 | 1.028 |
| P20042 | IF2B | 11.11 | 1 | 3 | 3 | 5 | 1.117 | 0.983 | 0.931 | 0.868 | 0.869 | 0.948 |
| Q86Y56 | HEAT2 | 7.37 | 3 | 5 | 5 | 8 | 1.005 | 0.982 | 1.016 | 1.018 | 1.008 | 0.985 |
| Q07812 | BAX | 7.29 | 1 | 2 | 2 | 2 | 0.864 | 0.982 | 1.134 | 0.997 | 1.151 | 1.011 |
| P05787 | K2C8 | 36.44 | 34 | 13 | 19 | 43 | 1.090 | 0.982 | 0.847 | 0.941 | 0.842 | 0.957 |
| B3KX72 | B3KX72 | 18.27 | 3 | 15 | 15 | 107 | 1.048 | 0.982 | 0.923 | 1.028 | 1.001 | 1.091 |
| B4DY73 | B4DY73 | 13.22 | 20 | 6 | 6 | 6 | 0.967 | 0.982 | 1.019 | 1.005 | 0.988 | 0.928 |
| Q6UB35 | C1TM | 2.76 | 2 | 2 | 3 | 3 | 0.931 | 0.981 | 1.052 | 1.058 | 1.132 | 1.073 |
| P49756 | RBM25 | 8.30 | 6 | 5 | 6 | 7 | 0.950 | 0.981 | 1.044 | 0.991 | 0.990 | 1.001 |
| P07195 | LDHB | 30.24 | 4 | 9 | 9 | 66 | 0.977 | 0.981 | 0.997 | 0.987 | 1.002 | 1.034 |
| Q06830 | PRDX1 | 49.75 | 1 | 7 | 10 | 138 | 1.001 | 0.981 | 0.990 | 1.017 | 1.014 | 1.026 |
| P61221 | ABCE1 | 14.19 | 3 | 8 | 8 | 19 | 1.103 | 0.981 | 0.918 | 1.104 | 0.999 | 1.132 |
| Q14978 | NOLC1 | 9.73 | 1 | 6 | 6 | 42 | 1.118 | 0.980 | 0.841 | 0.940 | 0.770 | 1.006 |
| Q9Y295 | DRG1 | 9.54 | 1 | 3 | 3 | 3 | 0.967 | 0.980 | 1.012 | 0.988 | 1.026 | 1.008 |
| P60174 | TPIS | 33.57 | 2 | 9 | 9 | 65 | 1.006 | 0.980 | 0.992 | 1.006 | 1.007 | 1.030 |
| P62269 | RS18 | 30.92 | 2 | 6 | 6 | 17 | 1.256 | 0.980 | 0.761 | 1.205 | 1.005 | 1.264 |
| P30049 | ATPD | 13.69 | 1 | 2 | 2 | 3 | 0.959 | 0.980 | 0.875 | 1.077 | 1.119 | 1.035 |
| O75643 | U520 | 12.13 | 6 | 20 | 20 | 32 | 0.939 | 0.979 | 1.014 | 0.989 | 1.057 | 1.010 |
| Q06203 | PUR1 | 6.19 | 2 | 3 | 3 | 4 | 0.999 | 0.979 | 0.991 | 0.966 | 0.983 | 1.027 |
| P62304 | RUXE | 29.35 | 3 | 2 | 2 | 29 | 1.074 | 0.979 | 0.952 | 0.966 | 0.897 | 0.965 |
| P63104 | 1433Z | 46.53 | 10 | 7 | 11 | 75 | 0.982 | 0.979 | 1.064 | 1.020 | 1.035 | 1.010 |
| E7ET84 | E7ET84 | 5.31 | 4 | 2 | 2 | 2 | 1.038 | 0.979 | 0.941 | 1.031 | 0.990 | 1.049 |
| P43034 | LIS1 | 6.34 | 3 | 2 | 2 | 2 | 0.969 | 0.978 | 1.007 | 0.975 | 1.003 | 0.992 |
| Q9UII2 | ATIF1 | 14.15 | 1 | 2 | 2 | 4 | 0.861 | 0.978 | 1.164 | 0.977 | 1.132 | 0.969 |
| E9PAV2 | E9PAV2 | 40.63 | 2 | 6 | 6 | 19 | 0.964 | 0.978 | 0.989 | 0.989 | 1.013 | 1.012 |
| P20674 | COX5A | 32.00 | 1 | 5 | 5 | 7 | 0.954 | 0.978 | 0.964 | 0.956 | 1.011 | 0.989 |
| P28070 | PSB4 | 28.03 | 1 | 4 | 4 | 19 | 0.949 | 0.978 | 1.033 | 1.029 | 1.068 | 1.015 |
| C9JCJ5 | C9JCJ5 | 14.55 | 2 | 2 | 2 | 3 | 1.140 | 0.977 | 0.859 | 0.956 | 1.009 | 1.141 |
| P21964 | COMT | 9.23 | 4 | 3 | 3 | 6 | 0.911 | 0.977 | 0.997 | 0.827 | 0.984 | 0.921 |
| O95373 | IPO7 | 5.39 | 2 | 4 | 4 | 7 | 0.940 | 0.977 | 1.000 | 0.941 | 0.987 | 0.988 |
| Q9NZI8 | IF2B1 | 17.50 | 2 | 6 | 9 | 35 | 0.946 | 0.977 | 1.043 | 0.952 | 1.010 | 1.036 |
| B8ZZ51 | B8ZZ51 | 16.57 | 5 | 3 | 3 | 3 | 1.052 | 0.977 | 0.963 | 1.011 | 0.985 | 0.995 |
| P52272 | HNRPM | 31.23 | 2 | 19 | 19 | 92 | 0.963 | 0.976 | 1.017 | 0.996 | 1.000 | 0.976 |
| P07741 | APT | 33.89 | 1 | 7 | 7 | 41 | 0.990 | 0.976 | 1.007 | 0.933 | 0.957 | 0.962 |
| P49411 | EFTU | 29.87 | 1 | 12 | 12 | 48 | 0.997 | 0.976 | 0.953 | 0.979 | 0.985 | 1.023 |
| E7ERW8 | E7ERW8 | 9.38 | 10 | 9 | 9 | 19 | 0.964 | 0.976 | 1.043 | 0.865 | 0.986 | 0.916 |
| Q9H9T3 | ELP3 | 5.30 | 10 | 2 | 2 | 2 | 1.050 | 0.976 | 0.927 | 1.143 | 1.085 | 1.166 |
| F8WA87 | F8WA87 | 2.60 | 3 | 5 | 5 | 6 | 0.950 | 0.975 | 1.079 | 1.080 | 1.013 | 1.040 |
| Q01085 | TIAR | 9.87 | 7 | 2 | 3 | 3 | 1.017 | 0.975 | 0.957 | 0.760 | 0.744 | 0.775 |
| P38117 | ETFB | 29.02 | 1 | 7 | 7 | 17 | 1.096 | 0.975 | 0.943 | 1.024 | 0.976 | 1.050 |
| B4DJP7 | B4DJP7 | 15.83 | 2 | 2 | 2 | 22 | 1.166 | 0.975 | 0.835 | 1.211 | 1.030 | 1.225 |
| P62899 | RL31 | 40.00 | 7 | 5 | 5 | 20 | 1.132 | 0.975 | 0.851 | 1.141 | 1.010 | 1.173 |
| Q96H99 | Q96H99 | 4.87 | 3 | 2 | 2 | 2 | 1.036 | 0.975 | 0.939 | 1.059 | 1.018 | 1.081 |
| P11413 | G6PD | 28.54 | 5 | 14 | 14 | 44 | 0.969 | 0.974 | 1.022 | 1.011 | 1.019 | 0.987 |
| P14868 | SYDC | 23.75 | 4 | 10 | 10 | 26 | 0.965 | 0.974 | 1.023 | 1.049 | 1.006 | 0.971 |
| P04406 | G3P | 36.72 | 6 | 11 | 11 | 233 | 1.007 | 0.974 | 0.963 | 1.014 | 0.995 | 1.026 |
| Q96PK6 | RBM14 | 11.66 | 6 | 7 | 7 | 8 | 1.009 | 0.974 | 0.974 | 0.958 | 0.919 | 0.979 |
| P09543 | CN37 | 8.31 | 2 | 3 | 3 | 3 | 0.814 | 0.974 | 1.193 | 0.927 | 1.149 | 0.872 |
| O75821 | EIF3G | 17.19 | 1 | 5 | 5 | 16 | 1.002 | 0.974 | 0.960 | 0.862 | 0.883 | 0.901 |
| O60502 | NCOAT | 6.66 | 2 | 4 | 4 | 7 | 0.834 | 0.973 | 1.146 | 0.839 | 1.042 | 0.869 |
| O95816 | BAG2 | 36.97 | 3 | 7 | 7 | 17 | 1.011 | 0.973 | 0.924 | 0.978 | 0.937 | 1.023 |
| B7Z9L0 | B7Z9L0 | 42.03 | 4 | 16 | 16 | 50 | 0.959 | 0.973 | 1.000 | 0.947 | 0.945 | 0.982 |
| P25398 | RS12 | 13.64 | 1 | 2 | 2 | 34 | 0.989 | 0.973 | 1.001 | 1.050 | 1.073 | 1.068 |
| P22102 | PUR2 | 12.48 | 7 | 12 | 12 | 33 | 1.001 | 0.973 | 0.934 | 0.963 | 0.957 | 1.012 |
| P31153 | METK2 | 11.90 | 4 | 5 | 5 | 12 | 1.006 | 0.973 | 0.982 | 1.014 | 1.020 | 1.038 |
| Q9BSJ8 | ESYT1 | 17.12 | 3 | 13 | 13 | 39 | 1.018 | 0.972 | 0.958 | 0.950 | 0.890 | 1.029 |
| P06744 | G6PI | 27.42 | 3 | 13 | 13 | 53 | 1.020 | 0.972 | 0.946 | 1.002 | 0.984 | 1.015 |
| P62917 | RL8 | 15.95 | 7 | 5 | 5 | 5 | 2.109 | 0.972 | 0.414 | 1.983 | 0.943 | 2.018 |
| O14818 | PSA7 | 23.79 | 6 | 5 | 5 | 7 | 1.045 | 0.971 | 0.977 | 1.023 | 0.976 | 0.982 |
| P00558 | PGK1 | 39.09 | 5 | 12 | 15 | 101 | 1.002 | 0.971 | 0.981 | 1.005 | 1.005 | 1.019 |
| Q99497 | PARK7 | 38.62 | 1 | 6 | 6 | 40 | 0.977 | 0.971 | 1.028 | 0.987 | 1.034 | 0.998 |
| P23528 | COF1 | 42.77 | 11 | 7 | 8 | 76 | 1.019 | 0.971 | 0.990 | 1.014 | 1.013 | 1.035 |
| Q13185 | CBX3 | 20.77 | 4 | 3 | 3 | 14 | 0.973 | 0.970 | 1.040 | 0.829 | 0.905 | 0.806 |
| Q75MG1 | Q75MG1 | 22.63 | 12 | 4 | 5 | 10 | 1.074 | 0.970 | 0.914 | 0.857 | 0.794 | 0.863 |
| Q13123 | RED | 5.21 | 6 | 3 | 3 | 3 | 0.869 | 0.970 | 1.126 | 0.902 | 0.927 | 0.917 |
| Q86V81 | THOC4 | 8.17 | 2 | 2 | 2 | 13 | 0.975 | 0.970 | 0.965 | 0.952 | 1.000 | 1.032 |
| P62333 | PRS10 | 25.96 | 1 | 8 | 8 | 24 | 0.970 | 0.970 | 0.995 | 0.987 | 1.008 | 1.004 |
| P26038 | MOES | 43.15 | 2 | 24 | 30 | 154 | 0.957 | 0.969 | 1.009 | 0.947 | 1.003 | 0.995 |
| Q16531 | DDB1 | 8.25 | 16 | 8 | 9 | 21 | 0.997 | 0.969 | 1.019 | 1.068 | 1.047 | 1.059 |
| Q15233 | NONO | 26.96 | 5 | 9 | 11 | 42 | 1.012 | 0.969 | 1.001 | 1.061 | 1.060 | 1.055 |
| P55769 | NH2L1 | 14.84 | 2 | 2 | 2 | 10 | 0.960 | 0.969 | 1.020 | 0.992 | 1.033 | 1.009 |
| P62701 | RS4X | 34.22 | 6 | 9 | 9 | 31 | 1.217 | 0.969 | 0.821 | 1.214 | 0.969 | 1.239 |
| P39748 | FEN1 | 4.74 | 1 | 2 | 2 | 3 | 1.031 | 0.969 | 0.871 | 1.121 | 1.077 | 1.127 |
| O15143 | ARC1B | 8.60 | 2 | 2 | 2 | 2 | 1.007 | 0.968 | 0.960 | 0.998 | 0.988 | 1.026 |
| Q9BUF5 | TBB6 | 33.63 | 3 | 2 | 12 | 174 | 1.104 | 0.968 | 0.875 | 0.963 | 0.870 | 0.990 |
| Q9P258 | RCC2 | 11.49 | 1 | 4 | 4 | 32 | 0.902 | 0.968 | 1.064 | 0.970 | 1.020 | 1.024 |
| P04843 | RPN1 | 28.67 | 5 | 17 | 17 | 51 | 0.994 | 0.968 | 0.982 | 0.982 | 0.996 | 1.025 |
| P10644 | KAP0 | 15.49 | 1 | 4 | 4 | 7 | 0.999 | 0.968 | 1.025 | 0.943 | 0.982 | 0.829 |
| E9PCQ3 | E9PCQ3 | 4.60 | 6 | 4 | 4 | 5 | 1.095 | 0.968 | 0.889 | 0.946 | 0.872 | 1.020 |
| Q96KP4 | CNDP2 | 7.58 | 1 | 3 | 3 | 4 | 1.090 | 0.967 | 0.886 | 1.038 | 0.908 | 0.862 |
| P42285 | SK2L2 | 7.01 | 2 | 6 | 6 | 8 | 1.096 | 0.967 | 0.991 | 1.093 | 0.954 | 1.133 |
| P28072 | PSB6 | 8.79 | 1 | 2 | 2 | 3 | 1.068 | 0.967 | 0.903 | 1.007 | 0.940 | 1.042 |
| P54136 | SYRC | 15.00 | 4 | 10 | 10 | 28 | 0.952 | 0.967 | 1.026 | 0.974 | 1.007 | 1.027 |
| P49588 | SYAC | 17.98 | 2 | 14 | 14 | 50 | 0.977 | 0.966 | 1.070 | 0.943 | 0.999 | 0.966 |
| Q08752 | PPID | 26.22 | 1 | 10 | 11 | 28 | 1.024 | 0.966 | 0.985 | 0.975 | 0.957 | 0.980 |
| A8K318 | A8K318 | 10.67 | 3 | 8 | 8 | 11 | 0.933 | 0.966 | 1.018 | 0.935 | 1.021 | 1.010 |
| F2Z2J9 | F2Z2J9 | 30.71 | 5 | 7 | 7 | 63 | 1.025 | 0.966 | 0.937 | 1.013 | 0.993 | 1.034 |
| O75937 | DNJC8 | 12.65 | 1 | 4 | 4 | 5 | 0.917 | 0.965 | 1.173 | 0.848 | 0.957 | 0.892 |
| F5H801 | F5H801 | 8.52 | 9 | 6 | 6 | 14 | 1.122 | 0.965 | 1.050 | 1.169 | 1.095 | 1.049 |
| Q9UQ80 | PA2G4 | 30.96 | 6 | 13 | 14 | 60 | 0.991 | 0.965 | 0.993 | 0.967 | 0.979 | 1.013 |
| P23381 | SYWC | 20.38 | 7 | 7 | 7 | 22 | 0.964 | 0.965 | 0.934 | 0.962 | 0.876 | 0.927 |
| C9JHK9 | C9JHK9 | 8.33 | 3 | 2 | 2 | 15 | 1.040 | 0.964 | 0.970 | 0.961 | 0.907 | 1.068 |
| P62263 | RS14 | 38.41 | 2 | 5 | 5 | 26 | 1.173 | 0.964 | 0.806 | 0.994 | 0.899 | 1.019 |
| P22626 | ROA2 | 57.51 | 1 | 17 | 21 | 170 | 0.979 | 0.963 | 1.008 | 0.998 | 1.015 | 1.022 |
| P53621 | COPA | 8.09 | 1 | 8 | 8 | 10 | 0.912 | 0.963 | 0.853 | 0.925 | 0.987 | 0.938 |
| Q9GZU8 | F192A | 8.66 | 2 | 2 | 2 | 2 | 1.212 | 0.963 | 0.793 | 1.138 | 0.936 | 1.176 |
| F5H8E5 | F5H8E5 | 3.79 | 3 | 3 | 3 | 20 | 1.019 | 0.963 | 0.998 | 0.959 | 0.891 | 0.955 |
| O75822 | EIF3J | 30.62 | 3 | 6 | 6 | 13 | 1.014 | 0.963 | 0.937 | 0.870 | 0.912 | 0.980 |
| E7EQH7 | E7EQH7 | 6.24 | 2 | 2 | 2 | 4 | 0.881 | 0.963 | 1.159 | 1.145 | 1.067 | 0.881 |
| E9PPJ0 | E9PPJ0 | 8.77 | 5 | 7 | 7 | 7 | 1.036 | 0.962 | 0.911 | 1.025 | 1.002 | 1.056 |
| F5GXH1 | F5GXH1 | 10.00 | 2 | 4 | 5 | 20 | 0.880 | 0.962 | 1.067 | 1.053 | 1.197 | 1.089 |
| P14866 | HNRPL | 12.56 | 3 | 6 | 6 | 18 | 1.050 | 0.962 | 0.923 | 1.010 | 0.931 | 1.037 |
| Q32Q12 | Q32Q12 | 57.53 | 7 | 4 | 12 | 115 | 1.002 | 0.962 | 0.936 | 0.975 | 0.953 | 1.041 |
| P62937 | PPIA | 40.00 | 9 | 8 | 10 | 155 | 0.973 | 0.962 | 0.981 | 1.007 | 1.009 | 1.038 |
| Q08211 | DHX9 | 19.84 | 2 | 20 | 20 | 94 | 0.977 | 0.962 | 0.935 | 0.994 | 1.010 | 1.029 |
| P05164 | PERM | 9.80 | 1 | 7 | 7 | 56 | 1.014 | 0.961 | 0.926 | 0.990 | 0.994 | 1.017 |
| P18206 | VINC | 19.05 | 4 | 18 | 18 | 37 | 1.014 | 0.961 | 1.030 | 0.966 | 0.995 | 1.007 |
| P23396 | RS3 | 62.14 | 10 | 15 | 15 | 70 | 1.022 | 0.961 | 0.941 | 0.965 | 0.962 | 1.010 |
| P19784 | CSK22 | 19.14 | 1 | 4 | 5 | 8 | 0.961 | 0.961 | 0.852 | 0.866 | 0.948 | 1.056 |
| B4DRU0 | B4DRU0 | 17.28 | 12 | 9 | 9 | 14 | 0.983 | 0.961 | 1.019 | 0.896 | 0.880 | 0.887 |
| P62805 | H4 | 52.43 | 1 | 7 | 7 | 105 | 0.988 | 0.961 | 0.987 | 0.971 | 0.953 | 0.984 |
| B4DEH8 | B4DEH8 | 19.64 | 4 | 4 | 4 | 5 | 0.933 | 0.961 | 1.087 | 1.016 | 1.176 | 1.000 |
| Q08945 | SSRP1 | 5.64 | 1 | 4 | 4 | 5 | 1.123 | 0.961 | 0.928 | 0.996 | 0.884 | 0.900 |
| P63241 | IF5A1 | 28.57 | 6 | 4 | 4 | 49 | 1.003 | 0.961 | 0.929 | 0.962 | 0.938 | 0.976 |
| P18077 | RL35A | 29.09 | 4 | 5 | 5 | 11 | 1.245 | 0.960 | 0.813 | 1.191 | 0.960 | 1.258 |
| P00338 | LDHA | 42.77 | 17 | 13 | 13 | 146 | 1.061 | 0.960 | 0.904 | 0.953 | 0.912 | 0.979 |
| Q15365 | PCBP1 | 27.53 | 17 | 5 | 8 | 28 | 0.956 | 0.960 | 0.983 | 0.900 | 0.946 | 0.953 |
| P68036 | UB2L3 | 36.36 | 5 | 6 | 6 | 28 | 0.951 | 0.960 | 0.989 | 0.913 | 0.920 | 0.969 |
| P62491 | RB11A | 31.48 | 3 | 7 | 7 | 20 | 0.973 | 0.960 | 0.988 | 1.008 | 0.963 | 1.025 |
| P62277 | RS13 | 38.41 | 2 | 6 | 6 | 30 | 1.281 | 0.960 | 0.742 | 1.360 | 1.042 | 1.383 |
| B7Z1I2 | B7Z1I2 | 14.75 | 3 | 4 | 4 | 7 | 1.049 | 0.959 | 0.916 | 0.982 | 0.969 | 1.072 |
| Q13263 | TIF1B | 17.84 | 1 | 14 | 14 | 39 | 0.958 | 0.959 | 0.949 | 0.942 | 0.974 | 0.988 |
| B4DV79 | B4DV79 | 20.60 | 5 | 12 | 12 | 35 | 0.952 | 0.959 | 0.977 | 0.986 | 1.001 | 1.037 |
| Q9BRA2 | TXD17 | 19.51 | 1 | 2 | 2 | 10 | 0.964 | 0.959 | 0.946 | 0.944 | 0.976 | 1.011 |
| P33316 | DUT | 29.76 | 1 | 5 | 5 | 30 | 0.989 | 0.959 | 0.954 | 0.964 | 1.001 | 0.998 |
| Q68DB1 | Q68DB1 | 8.28 | 2 | 4 | 4 | 13 | 1.147 | 0.958 | 0.968 | 1.113 | 1.001 | 1.116 |
| B4E0X8 | B4E0X8 | 11.76 | 13 | 4 | 7 | 12 | 0.982 | 0.958 | 0.974 | 0.903 | 0.934 | 0.972 |
| B4DHI8 | B4DHI8 | 12.33 | 15 | 8 | 8 | 14 | 1.002 | 0.957 | 0.983 | 0.861 | 0.881 | 0.897 |
| O15160 | RPAC1 | 17.34 | 5 | 4 | 4 | 4 | 1.096 | 0.957 | 0.957 | 1.062 | 1.298 | 1.141 |
| E9PLL6 | E9PLL6 | 16.67 | 3 | 2 | 2 | 3 | 1.230 | 0.957 | 0.777 | 1.052 | 0.852 | 1.094 |
| P30084 | ECHM | 9.66 | 1 | 3 | 3 | 5 | 0.976 | 0.957 | 0.979 | 0.963 | 1.003 | 1.010 |
| C9JWD4 | C9JWD4 | 15.16 | 7 | 3 | 3 | 3 | 0.991 | 0.957 | 1.105 | 1.010 | 1.017 | 1.004 |
| B2BCH7 | B2BCH7 | 17.51 | 3 | 2 | 2 | 3 | 0.810 | 0.957 | 1.163 | 0.944 | 1.053 | 0.902 |
| Q9UNE7 | CHIP | 5.94 | 1 | 2 | 2 | 3 | 0.920 | 0.957 | 1.012 | 0.937 | 1.163 | 1.137 |
| Q09666 | AHNK | 4.62 | 2 | 8 | 8 | 12 | 1.033 | 0.957 | 0.934 | 0.948 | 0.822 | 0.847 |
| B1AH77 | B1AH77 | 9.46 | 6 | 2 | 2 | 3 | 1.129 | 0.957 | 0.878 | 0.947 | 0.868 | 0.986 |
| P82979 | SARNP | 17.62 | 2 | 3 | 3 | 3 | 1.139 | 0.956 | 1.003 | 1.012 | 1.042 | 1.036 |
| F5H7Y0 | F5H7Y0 | 4.85 | 6 | 2 | 2 | 2 | 1.035 | 0.955 | 0.921 | 1.155 | 1.112 | 1.203 |
| Q9H223 | EHD4 | 11.83 | 2 | 3 | 5 | 8 | 1.276 | 0.954 | 0.746 | 1.047 | 0.966 | 1.100 |
| P68104 | EF1A1 | 32.25 | 8 | 15 | 15 | 222 | 0.996 | 0.954 | 0.947 | 0.969 | 0.980 | 1.033 |
| Q9Y5S9 | RBM8A | 22.41 | 1 | 3 | 3 | 11 | 0.992 | 0.954 | 1.015 | 0.959 | 0.956 | 0.966 |
| F5H2A7 | F5H2A7 | 11.46 | 2 | 2 | 2 | 3 | 1.193 | 0.953 | 0.781 | 0.844 | 0.690 | 0.881 |
| Q9BYG3 | MK67I | 9.22 | 3 | 2 | 2 | 2 | 1.085 | 0.953 | 0.877 | 1.095 | 1.005 | 1.143 |
| F5H1F8 | F5H1F8 | 9.66 | 8 | 2 | 2 | 2 | 1.098 | 0.952 | 0.866 | 0.924 | 0.839 | 0.965 |
| B4E241 | B4E241 | 24.19 | 2 | 3 | 4 | 18 | 0.994 | 0.952 | 0.942 | 0.983 | 0.947 | 1.003 |
| P43243 | MATR3 | 20.54 | 19 | 15 | 15 | 66 | 1.016 | 0.952 | 0.993 | 0.988 | 0.996 | 1.011 |
| B2REB8 | B2REB8 | 16.98 | 4 | 5 | 5 | 43 | 0.935 | 0.952 | 0.997 | 0.955 | 1.018 | 1.011 |
| P08621 | RU17 | 12.81 | 1 | 6 | 6 | 17 | 0.987 | 0.952 | 0.948 | 0.968 | 1.005 | 0.951 |
| Q8TCS8 | PNPT1 | 4.60 | 1 | 3 | 3 | 4 | 1.044 | 0.952 | 0.891 | 0.953 | 0.901 | 1.008 |
| P02786 | TFR1 | 26.84 | 5 | 17 | 17 | 98 | 1.036 | 0.952 | 0.913 | 0.972 | 0.969 | 1.038 |
| B4DQJ8 | B4DQJ8 | 34.68 | 5 | 15 | 15 | 65 | 0.980 | 0.952 | 0.987 | 0.966 | 0.979 | 1.032 |
| B4DFF1 | B4DFF1 | 36.60 | 1 | 2 | 18 | 134 | 0.908 | 0.952 | 1.046 | 1.020 | 1.120 | 1.066 |
| A8MUB1 | A8MUB1 | 42.26 | 10 | 2 | 16 | 139 | 0.651 | 0.952 | 1.292 | 1.016 | 1.442 | 1.025 |
| Q9NRX4 | PHP14 | 20.80 | 1 | 3 | 3 | 3 | 0.980 | 0.951 | 0.904 | 1.143 | 0.936 | 1.095 |
| B0AZP7 | B0AZP7 | 18.53 | 3 | 3 | 3 | 15 | 1.040 | 0.951 | 0.983 | 1.062 | 0.972 | 1.106 |
| P31946 | 1433B | 37.40 | 5 | 3 | 9 | 82 | 0.960 | 0.950 | 0.992 | 1.004 | 0.970 | 0.921 |
| P15880 | RS2 | 30.03 | 6 | 9 | 9 | 62 | 1.111 | 0.950 | 0.871 | 1.161 | 1.043 | 1.197 |
| P26599 | PTBP1 | 21.85 | 6 | 6 | 8 | 35 | 0.930 | 0.950 | 0.997 | 0.972 | 1.067 | 1.048 |
| P04075 | ALDOA | 42.58 | 4 | 15 | 17 | 167 | 1.030 | 0.950 | 0.922 | 0.985 | 0.948 | 1.017 |
| P62424 | RL7A | 15.79 | 3 | 4 | 4 | 6 | 1.176 | 0.950 | 0.767 | 1.210 | 1.005 | 1.280 |
| P98179 | RBM3 | 21.02 | 1 | 2 | 2 | 3 | 0.997 | 0.949 | 0.951 | 0.929 | 0.984 | 0.998 |
| P40227 | TCPZ | 27.50 | 6 | 13 | 13 | 88 | 0.957 | 0.949 | 0.950 | 0.911 | 0.946 | 1.004 |
| O76021 | RL1D1 | 6.94 | 2 | 3 | 3 | 6 | 1.544 | 0.949 | 0.693 | 1.283 | 0.889 | 1.200 |
| Q15717 | ELAV1 | 30.06 | 1 | 7 | 7 | 18 | 0.964 | 0.949 | 0.939 | 1.047 | 1.015 | 1.066 |
| Q09161 | NCBP1 | 4.56 | 1 | 3 | 3 | 4 | 0.962 | 0.949 | 1.152 | 1.044 | 1.029 | 0.882 |
| Q92900 | RENT1 | 9.30 | 1 | 8 | 8 | 14 | 1.049 | 0.949 | 0.918 | 1.103 | 1.026 | 1.110 |
| E9PDE8 | E9PDE8 | 7.13 | 4 | 2 | 5 | 14 | 0.919 | 0.949 | 1.030 | 0.887 | 0.962 | 0.931 |
| P14625 | ENPL | 27.52 | 5 | 20 | 22 | 103 | 1.007 | 0.948 | 0.926 | 0.938 | 0.906 | 0.995 |
| Q9BWL6 | Q9BWL6 | 5.85 | 2 | 2 | 2 | 3 | 1.083 | 0.948 | 0.946 | 1.172 | 1.205 | 1.270 |
| B1AK87 | B1AK87 | 22.69 | 7 | 5 | 5 | 25 | 0.969 | 0.948 | 1.033 | 0.917 | 0.966 | 0.988 |
| Q5JY90 | Q5JY90 | 14.29 | 2 | 4 | 4 | 8 | 0.878 | 0.948 | 1.140 | 0.976 | 1.108 | 0.956 |
| B2RDM2 | B2RDM2 | 20.37 | 3 | 6 | 6 | 16 | 1.039 | 0.948 | 0.965 | 0.892 | 0.957 | 1.028 |
| P12268 | IMDH2 | 27.43 | 9 | 11 | 11 | 27 | 1.001 | 0.948 | 0.914 | 0.931 | 0.931 | 0.971 |
| P24534 | EF1B | 19.56 | 4 | 4 | 4 | 36 | 0.981 | 0.947 | 0.960 | 0.962 | 0.979 | 1.001 |
| Q13347 | EIF3I | 21.23 | 1 | 6 | 6 | 13 | 1.011 | 0.947 | 0.964 | 1.002 | 0.969 | 1.024 |
| E7EQV9 | E7EQV9 | 12.07 | 5 | 2 | 2 | 6 | 2.308 | 0.947 | 0.474 | 1.300 | 0.590 | 1.277 |
| P50454 | SERPH | 26.79 | 13 | 7 | 7 | 16 | 1.037 | 0.947 | 0.889 | 1.050 | 0.949 | 1.054 |
| B7Z4V2 | B7Z4V2 | 38.20 | 5 | 23 | 24 | 134 | 0.968 | 0.947 | 0.970 | 0.973 | 0.988 | 0.996 |
| P42704 | LPPRC | 17.93 | 5 | 20 | 20 | 82 | 1.013 | 0.947 | 0.949 | 0.960 | 0.950 | 1.000 |
| Q60FE7 | Q60FE7 | 8.03 | 4 | 15 | 17 | 34 | 1.013 | 0.946 | 0.983 | 1.025 | 0.962 | 1.023 |
| Q5JR95 | Q5JR95 | 36.17 | 2 | 6 | 6 | 18 | 1.230 | 0.946 | 0.745 | 1.137 | 0.897 | 1.240 |
| P31689 | DNJA1 | 14.61 | 3 | 4 | 4 | 23 | 0.889 | 0.945 | 0.947 | 0.897 | 0.971 | 0.981 |
| P05387 | RLA2 | 40.87 | 1 | 4 | 4 | 32 | 0.948 | 0.944 | 0.968 | 0.903 | 0.893 | 0.953 |
| P28838 | AMPL | 9.63 | 1 | 5 | 5 | 22 | 0.914 | 0.944 | 1.017 | 0.953 | 1.029 | 0.987 |
| P52888 | THOP1 | 9.72 | 2 | 5 | 5 | 5 | 1.025 | 0.944 | 0.944 | 1.059 | 1.148 | 1.155 |
| F8VPD4 | F8VPD4 | 4.53 | 3 | 9 | 9 | 11 | 0.992 | 0.944 | 0.892 | 1.016 | 0.960 | 0.997 |
| Q14444 | CAPR1 | 10.72 | 5 | 9 | 9 | 17 | 1.025 | 0.944 | 0.841 | 0.868 | 0.813 | 0.939 |
| Q5T123 | Q5T123 | 32.95 | 2 | 2 | 2 | 8 | 1.055 | 0.943 | 0.970 | 0.995 | 0.971 | 0.955 |
| O00148 | DX39A | 32.08 | 8 | 4 | 14 | 88 | 0.965 | 0.943 | 0.999 | 1.022 | 1.006 | 1.070 |
| O00442 | RTC1 | 5.46 | 1 | 2 | 2 | 3 | 0.859 | 0.943 | 1.109 | 1.007 | 1.170 | 0.672 |
| Q99714 | HCD2 | 21.84 | 2 | 3 | 3 | 6 | 0.963 | 0.943 | 1.005 | 0.977 | 1.001 | 1.045 |
| F2Z3D0 | F2Z3D0 | 30.99 | 7 | 2 | 2 | 11 | 1.128 | 0.943 | 0.911 | 1.138 | 1.001 | 1.122 |
| P62244 | RS15A | 44.62 | 1 | 5 | 5 | 46 | 1.008 | 0.943 | 0.888 | 0.974 | 0.914 | 1.076 |
| O75153 | K0664 | 2.75 | 3 | 4 | 4 | 4 | 1.023 | 0.942 | 0.894 | 0.929 | 0.908 | 1.013 |
| P00441 | SODC | 13.64 | 2 | 2 | 2 | 15 | 0.925 | 0.942 | 1.000 | 0.946 | 1.017 | 1.048 |
| F5H0F5 | F5H0F5 | 6.12 | 3 | 2 | 2 | 12 | 0.987 | 0.941 | 0.963 | 0.984 | 0.965 | 1.040 |
| P13010 | XRCC5 | 26.23 | 2 | 14 | 14 | 30 | 1.030 | 0.941 | 0.940 | 1.008 | 0.988 | 1.004 |
| Q9Y265 | RUVB1 | 25.44 | 2 | 9 | 9 | 25 | 0.929 | 0.941 | 0.995 | 0.985 | 1.003 | 1.073 |
| P19623 | SPEE | 10.60 | 1 | 3 | 3 | 14 | 1.058 | 0.941 | 0.918 | 0.914 | 0.893 | 0.965 |
| F8VZJ2 | F8VZJ2 | 41.91 | 7 | 4 | 4 | 17 | 0.949 | 0.940 | 0.994 | 0.973 | 1.002 | 1.032 |
| P18583 | SON | 1.48 | 1 | 2 | 2 | 4 | 1.150 | 0.940 | 0.905 | 0.944 | 0.908 | 0.976 |
| Q8NC51 | PAIRB | 11.03 | 1 | 4 | 4 | 23 | 1.005 | 0.940 | 0.930 | 0.969 | 0.986 | 1.043 |
| P62826 | RAN | 25.00 | 4 | 5 | 5 | 69 | 0.989 | 0.939 | 0.918 | 0.944 | 0.958 | 1.033 |
| Q13643 | FHL3 | 5.00 | 1 | 2 | 2 | 2 | 1.077 | 0.939 | 0.870 | 1.030 | 0.953 | 1.091 |
| E7ENU2 | E7ENU2 | 3.14 | 5 | 3 | 4 | 4 | 0.908 | 0.939 | 0.996 | 1.056 | 1.111 | 1.112 |
| C9JXV8 | C9JXV8 | 6.50 | 3 | 3 | 3 | 5 | 1.031 | 0.939 | 0.917 | 1.029 | 0.901 | 0.977 |
| Q99439 | CNN2 | 11.65 | 3 | 3 | 3 | 4 | 1.024 | 0.938 | 0.978 | 1.096 | 0.985 | 1.154 |
| O75340 | PDCD6 | 26.70 | 2 | 5 | 5 | 21 | 0.956 | 0.938 | 0.956 | 0.975 | 1.000 | 0.973 |
| G3V2B8 | G3V2B8 | 19.47 | 4 | 14 | 15 | 33 | 0.937 | 0.938 | 1.006 | 0.898 | 0.977 | 0.920 |
| P10809 | CH60 | 49.91 | 8 | 16 | 26 | 390 | 0.959 | 0.937 | 0.974 | 0.976 | 0.999 | 1.036 |
| Q86UE4 | LYRIC | 3.78 | 2 | 2 | 2 | 2 | 0.840 | 0.937 | 1.113 | 0.719 | 0.854 | 0.764 |
| O43592 | XPOT | 2.18 | 4 | 2 | 2 | 4 | 0.854 | 0.936 | 1.075 | 0.835 | 0.977 | 0.912 |
| Q00325 | MPCP | 8.01 | 2 | 3 | 3 | 4 | 0.987 | 0.936 | 0.939 | 0.830 | 0.894 | 0.933 |
| C9IZM0 | C9IZM0 | 2.91 | 5 | 2 | 2 | 3 | 1.029 | 0.936 | 0.960 | 0.974 | 0.926 | 0.940 |
| P05091 | ALDH2 | 22.82 | 12 | 9 | 11 | 40 | 0.957 | 0.936 | 0.998 | 0.969 | 1.013 | 1.004 |
| O00231 | PSD11 | 24.41 | 1 | 8 | 8 | 22 | 0.882 | 0.935 | 1.020 | 0.990 | 1.021 | 0.965 |
| F8VW92 | F8VW92 | 48.05 | 21 | 4 | 18 | 452 | 0.944 | 0.935 | 0.983 | 0.944 | 0.980 | 1.007 |
| B4E3E8 | B4E3E8 | 27.40 | 10 | 14 | 15 | 52 | 0.990 | 0.935 | 0.920 | 0.897 | 0.904 | 0.976 |
| P09012 | SNRPA | 16.31 | 2 | 2 | 4 | 13 | 1.024 | 0.935 | 0.949 | 0.857 | 0.835 | 0.917 |
| P62249 | RS16 | 38.36 | 2 | 8 | 8 | 47 | 1.294 | 0.935 | 0.725 | 1.286 | 0.982 | 1.350 |
| B4DN37 | B4DN37 | 13.49 | 5 | 4 | 4 | 8 | 1.069 | 0.935 | 0.934 | 0.925 | 1.014 | 1.089 |
| Q8WW12 | PCNP | 13.48 | 1 | 2 | 2 | 2 | 0.904 | 0.935 | 1.032 | 1.053 | 1.161 | 1.121 |
| P31948 | STIP1 | 30.57 | 5 | 16 | 16 | 88 | 1.002 | 0.935 | 0.943 | 0.909 | 0.924 | 0.981 |
| O00487 | PSDE | 17.10 | 2 | 4 | 4 | 6 | 0.906 | 0.934 | 1.029 | 0.917 | 0.983 | 0.900 |
| Q9Y617 | SERC | 6.22 | 1 | 2 | 2 | 2 | 1.080 | 0.934 | 0.863 | 1.059 | 0.978 | 1.129 |
| P26641 | EF1G | 25.86 | 4 | 12 | 12 | 99 | 0.958 | 0.934 | 0.953 | 0.923 | 0.934 | 1.007 |
| P54886 | P5CS | 2.52 | 1 | 2 | 2 | 3 | 0.882 | 0.933 | 1.076 | 0.926 | 0.897 | 0.987 |
| C9J9W2 | C9J9W2 | 20.48 | 5 | 3 | 3 | 3 | 0.935 | 0.933 | 0.996 | 0.989 | 1.013 | 1.132 |
| E7EUM5 | E7EUM5 | 6.73 | 3 | 2 | 2 | 3 | 0.855 | 0.933 | 1.089 | 0.956 | 1.115 | 1.020 |
| Q04760 | LGUL | 30.43 | 1 | 5 | 5 | 13 | 0.954 | 0.933 | 1.003 | 0.984 | 0.998 | 0.987 |
| P39019 | RS19 | 41.38 | 1 | 8 | 8 | 78 | 1.016 | 0.932 | 0.945 | 1.040 | 1.068 | 1.143 |
| P05198 | IF2A | 18.73 | 2 | 6 | 6 | 10 | 1.012 | 0.932 | 0.909 | 0.975 | 0.950 | 1.029 |
| P06748 | NPM | 35.03 | 4 | 9 | 9 | 177 | 0.974 | 0.931 | 0.943 | 0.937 | 0.961 | 1.007 |
| Q13283 | G3BP1 | 18.03 | 11 | 6 | 7 | 24 | 0.986 | 0.931 | 0.972 | 0.936 | 0.975 | 0.930 |
| B4DZC3 | B4DZC3 | 5.92 | 2 | 4 | 4 | 5 | 1.137 | 0.931 | 0.788 | 0.971 | 0.864 | 0.963 |
| A0AVT1 | UBA6 | 3.80 | 1 | 4 | 4 | 9 | 0.974 | 0.931 | 0.902 | 0.871 | 0.968 | 1.011 |
| P46782 | RS5 | 23.53 | 1 | 5 | 5 | 10 | 0.970 | 0.931 | 0.937 | 0.941 | 1.001 | 0.999 |
| Q8WUA2 | PPIL4 | 4.67 | 1 | 2 | 2 | 2 | 0.801 | 0.931 | 1.159 | 0.940 | 1.170 | 1.005 |
| P02768 | ALBU | 3.61 | 7 | 2 | 2 | 11 | 0.877 | 0.930 | 1.020 | 0.925 | 1.045 | 0.997 |
| B7Z4T9 | B7Z4T9 | 28.46 | 8 | 12 | 12 | 58 | 0.966 | 0.930 | 0.961 | 0.949 | 0.970 | 1.015 |
| E9PBU3 | E9PBU3 | 20.14 | 5 | 10 | 10 | 31 | 0.986 | 0.930 | 0.982 | 0.932 | 0.958 | 0.982 |
| F5H897 | F5H897 | 25.04 | 2 | 13 | 14 | 81 | 0.994 | 0.929 | 0.922 | 0.895 | 0.901 | 0.977 |
| P31146 | COR1A | 9.54 | 4 | 5 | 5 | 7 | 1.014 | 0.929 | 0.930 | 0.950 | 1.045 | 1.087 |
| G3V1I6 | G3V1I6 | 16.37 | 7 | 6 | 6 | 8 | 0.931 | 0.929 | 0.946 | 0.906 | 0.986 | 0.981 |
| P04792 | HSPB1 | 45.37 | 4 | 9 | 9 | 82 | 0.934 | 0.929 | 0.996 | 0.932 | 1.005 | 1.014 |
| P51610 | HCFC1 | 1.38 | 2 | 2 | 2 | 7 | 0.908 | 0.928 | 1.020 | 0.933 | 1.099 | 1.000 |
| P61160 | ARP2 | 24.62 | 4 | 8 | 9 | 23 | 0.980 | 0.927 | 1.038 | 0.981 | 0.980 | 0.924 |
| P60842 | IF4A1 | 47.04 | 9 | 14 | 16 | 118 | 0.987 | 0.927 | 0.942 | 0.902 | 0.927 | 0.976 |
| F5H668 | F5H668 | 22.86 | 8 | 3 | 4 | 4 | 0.977 | 0.927 | 1.072 | 1.096 | 1.084 | 1.008 |
| B4DHN0 | B4DHN0 | 17.53 | 4 | 4 | 5 | 6 | 0.906 | 0.926 | 0.967 | 0.970 | 0.936 | 1.018 |
| P0C0S5 | H2AZ | 31.25 | 9 | 3 | 5 | 63 | 0.976 | 0.926 | 0.947 | 1.068 | 1.114 | 1.256 |
| P11215 | ITAM | 2.00 | 1 | 2 | 2 | 7 | 0.837 | 0.926 | 1.038 | 0.907 | 1.052 | 0.994 |
| P07900 | HS90A | 47.68 | 8 | 17 | 36 | 399 | 1.027 | 0.925 | 0.923 | 0.972 | 0.951 | 1.016 |
| Q9Y3C6 | PPIL1 | 16.87 | 1 | 2 | 2 | 2 | 0.969 | 0.925 | 0.953 | 0.910 | 0.936 | 0.979 |
| B7Z3X5 | B7Z3X5 | 7.24 | 9 | 3 | 3 | 3 | 0.889 | 0.924 | 0.968 | 0.894 | 1.043 | 1.074 |
| Q5JR08 | Q5JR08 | 25.93 | 12 | 3 | 3 | 4 | 0.934 | 0.924 | 0.939 | 0.951 | 1.043 | 1.096 |
| Q15435 | PP1R7 | 10.28 | 5 | 3 | 3 | 4 | 0.773 | 0.924 | 1.123 | 1.003 | 1.173 | 1.014 |
| P13667 | PDIA4 | 22.02 | 3 | 13 | 13 | 32 | 1.017 | 0.924 | 0.921 | 0.987 | 1.001 | 1.098 |
| P62857 | RS28 | 36.23 | 1 | 3 | 3 | 20 | 0.975 | 0.923 | 0.931 | 0.915 | 0.921 | 0.987 |
| Q9NTK5 | OLA1 | 17.93 | 3 | 6 | 6 | 33 | 0.906 | 0.923 | 1.048 | 0.949 | 1.046 | 1.077 |
| Q13895 | BYST | 5.95 | 1 | 3 | 3 | 6 | 1.186 | 0.921 | 0.798 | 0.851 | 0.802 | 0.996 |
| E9PB90 | E9PB90 | 7.87 | 3 | 4 | 6 | 8 | 0.943 | 0.921 | 1.038 | 0.787 | 0.929 | 0.965 |
| Q9UHV9 | PFD2 | 14.94 | 1 | 2 | 2 | 3 | 1.080 | 0.921 | 0.951 | 0.955 | 0.901 | 1.032 |
| B7ZAM9 | B7ZAM9 | 17.06 | 2 | 4 | 4 | 6 | 0.969 | 0.921 | 0.906 | 0.857 | 0.822 | 0.922 |
| P60228 | EIF3E | 18.65 | 5 | 6 | 6 | 11 | 0.934 | 0.921 | 1.060 | 0.945 | 0.941 | 0.876 |
| P29350 | PTN6 | 3.53 | 2 | 2 | 2 | 3 | 1.039 | 0.921 | 0.880 | 1.079 | 1.034 | 1.166 |
| B4DDM6 | B4DDM6 | 10.89 | 2 | 2 | 2 | 6 | 0.918 | 0.921 | 0.971 | 0.821 | 0.962 | 0.957 |
| O75489 | NDUS3 | 10.61 | 5 | 2 | 2 | 2 | 0.759 | 0.920 | 1.211 | 0.834 | 1.096 | 0.903 |
| P13639 | EF2 | 30.54 | 5 | 23 | 24 | 260 | 1.003 | 0.920 | 0.902 | 0.917 | 0.893 | 0.989 |
| P0CW22 | RS17L | 45.93 | 1 | 5 | 5 | 29 | 1.016 | 0.919 | 0.908 | 1.057 | 1.016 | 1.111 |
| P61081 | UBC12 | 16.94 | 1 | 3 | 3 | 4 | 1.010 | 0.919 | 0.951 | 1.003 | 1.024 | 1.050 |
| Q9UBQ0 | VPS29 | 14.29 | 3 | 3 | 3 | 9 | 0.954 | 0.919 | 0.966 | 0.987 | 1.054 | 1.074 |
| B9EGA3 | B9EGA3 | 5.26 | 2 | 2 | 2 | 2 | 1.119 | 0.919 | 0.820 | 1.344 | 1.197 | 1.456 |
| Q9H0C8 | ILKAP | 7.91 | 2 | 3 | 3 | 5 | 0.989 | 0.918 | 0.941 | 1.000 | 0.969 | 1.081 |
| B3KQT9 | B3KQT9 | 26.88 | 3 | 14 | 14 | 98 | 0.957 | 0.918 | 0.977 | 0.956 | 0.978 | 1.019 |
| Q9Y5B9 | SP16H | 4.30 | 2 | 6 | 6 | 6 | 0.982 | 0.917 | 0.951 | 0.987 | 0.959 | 0.996 |
| G3XAC6 | G3XAC6 | 10.40 | 8 | 4 | 4 | 13 | 1.063 | 0.917 | 0.855 | 1.006 | 0.993 | 1.009 |
| P50990 | TCPQ | 47.08 | 3 | 26 | 26 | 108 | 0.981 | 0.917 | 0.946 | 0.928 | 0.939 | 0.993 |
| O60869 | EDF1 | 14.86 | 1 | 2 | 2 | 2 | 0.945 | 0.917 | 0.968 | 0.861 | 0.908 | 0.935 |
| Q8TEX9 | IPO4 | 6.48 | 1 | 4 | 4 | 15 | 0.880 | 0.916 | 1.017 | 0.888 | 0.965 | 0.949 |
| F5H6W1 | F5H6W1 | 8.08 | 4 | 3 | 3 | 6 | 0.978 | 0.916 | 0.911 | 0.965 | 1.066 | 1.074 |
| P04632 | CPNS1 | 22.01 | 2 | 4 | 4 | 4 | 0.965 | 0.916 | 0.810 | 0.926 | 0.981 | 1.007 |
| E9PAQ6 | E9PAQ6 | 24.63 | 9 | 13 | 13 | 107 | 0.959 | 0.915 | 0.963 | 0.938 | 0.974 | 1.012 |
| E7ENQ1 | E7ENQ1 | 1.21 | 12 | 2 | 2 | 5 | 1.030 | 0.915 | 0.886 | 0.942 | 0.920 | 1.034 |
| O14979 | HNRDL | 12.86 | 1 | 4 | 5 | 55 | 0.851 | 0.915 | 0.976 | 0.870 | 0.984 | 0.912 |
| E7EM71 | E7EM71 | 9.39 | 11 | 7 | 7 | 16 | 0.985 | 0.915 | 0.939 | 0.899 | 0.881 | 0.966 |
| P37802 | TAGL2 | 54.27 | 2 | 8 | 9 | 102 | 0.974 | 0.914 | 0.926 | 0.912 | 0.969 | 0.999 |
| B4DZI8 | B4DZI8 | 7.18 | 2 | 5 | 5 | 9 | 0.989 | 0.914 | 0.933 | 0.906 | 0.956 | 0.994 |
| B0UX83 | B0UX83 | 5.33 | 11 | 4 | 4 | 6 | 0.897 | 0.913 | 0.965 | 0.939 | 0.929 | 1.052 |
| Q9UQ35 | SRRM2 | 2.62 | 2 | 4 | 4 | 4 | 0.943 | 0.913 | 0.927 | 0.962 | 1.017 | 1.094 |
| P61289 | PSME3 | 23.23 | 3 | 5 | 5 | 17 | 1.001 | 0.913 | 0.857 | 1.005 | 0.918 | 1.048 |
| B0QYA5 | B0QYA5 | 15.61 | 7 | 4 | 4 | 6 | 0.928 | 0.913 | 0.991 | 0.933 | 0.933 | 0.953 |
| P50395 | GDIB | 31.91 | 9 | 12 | 12 | 72 | 0.979 | 0.913 | 0.964 | 0.921 | 0.968 | 0.998 |
| B3KQV1 | B3KQV1 | 13.91 | 2 | 2 | 2 | 2 | 1.069 | 0.913 | 0.852 | 0.728 | 0.679 | 0.794 |
| B3KMI0 | B3KMI0 | 5.28 | 2 | 3 | 3 | 4 | 1.000 | 0.912 | 0.924 | 0.975 | 0.867 | 1.085 |
| P36405 | ARL3 | 15.93 | 1 | 2 | 2 | 3 | 1.133 | 0.912 | 1.012 | 0.737 | 1.029 | 0.736 |
| F8W020 | F8W020 | 42.03 | 16 | 6 | 7 | 62 | 0.932 | 0.912 | 0.923 | 0.905 | 0.936 | 0.976 |
| P43490 | NAMPT | 11.20 | 3 | 4 | 4 | 4 | 0.949 | 0.912 | 0.929 | 0.979 | 1.041 | 1.083 |
| P25787 | PSA2 | 32.05 | 2 | 5 | 5 | 26 | 0.976 | 0.912 | 0.969 | 1.002 | 1.018 | 1.085 |
| E9PEL7 | E9PEL7 | 5.42 | 4 | 4 | 4 | 4 | 1.044 | 0.911 | 0.883 | 0.860 | 0.808 | 0.974 |
| E9PJS4 | E9PJS4 | 8.03 | 2 | 5 | 5 | 7 | 1.049 | 0.911 | 0.894 | 0.770 | 0.888 | 0.892 |
| P43686 | PRS6B | 19.86 | 1 | 6 | 6 | 21 | 1.005 | 0.910 | 0.896 | 0.811 | 0.931 | 1.052 |
| Q5VU59 | Q5VU59 | 50.00 | 18 | 9 | 18 | 39 | 0.988 | 0.910 | 0.931 | 0.981 | 0.956 | 0.994 |
| B0V0F8 | B0V0F8 | 3.89 | 5 | 2 | 2 | 2 | 1.195 | 0.909 | 0.759 | 0.980 | 0.818 | 1.074 |
| P49755 | TMEDA | 20.09 | 2 | 4 | 4 | 5 | 0.928 | 0.909 | 1.018 | 0.909 | 0.862 | 0.808 |
| P08238 | HS90B | 46.41 | 8 | 17 | 38 | 530 | 0.982 | 0.908 | 0.915 | 0.927 | 0.943 | 1.021 |
| E7EPA8 | E7EPA8 | 46.14 | 4 | 21 | 21 | 164 | 0.985 | 0.908 | 0.922 | 0.927 | 0.943 | 1.012 |
| Q02790 | FKBP4 | 14.16 | 3 | 8 | 8 | 17 | 1.001 | 0.907 | 0.890 | 0.976 | 0.917 | 1.022 |
| E9PN17 | E9PN17 | 31.58 | 2 | 2 | 2 | 13 | 0.905 | 0.907 | 1.012 | 0.929 | 1.018 | 0.964 |
| B7Z3U6 | B7Z3U6 | 3.93 | 17 | 3 | 3 | 7 | 0.959 | 0.907 | 0.962 | 0.914 | 0.921 | 0.951 |
| E7EU96 | E7EU96 | 17.40 | 3 | 3 | 4 | 7 | 0.939 | 0.906 | 0.946 | 0.852 | 0.886 | 0.982 |
| P67809 | YBOX1 | 13.58 | 4 | 2 | 3 | 8 | 1.066 | 0.906 | 0.906 | 1.044 | 0.992 | 1.091 |
| P26583 | HMGB2 | 11.48 | 2 | 2 | 2 | 3 | 0.999 | 0.906 | 0.848 | 0.829 | 0.827 | 0.972 |
| P19338 | NUCL | 30.28 | 7 | 23 | 23 | 140 | 1.011 | 0.906 | 0.876 | 0.964 | 0.936 | 1.080 |
| B4E322 | B4E322 | 11.89 | 7 | 3 | 3 | 4 | 1.040 | 0.905 | 1.023 | 1.003 | 1.144 | 0.979 |
| Q08J23 | NSUN2 | 10.04 | 4 | 7 | 7 | 8 | 0.947 | 0.904 | 0.984 | 1.028 | 1.009 | 1.047 |
| Q330K1 | Q330K1 | 7.50 | 2 | 2 | 2 | 29 | 0.924 | 0.904 | 0.950 | 0.906 | 0.961 | 0.991 |
| F8W1A4 | F8W1A4 | 47.41 | 7 | 8 | 8 | 37 | 0.980 | 0.904 | 0.938 | 0.903 | 0.914 | 0.999 |
| E7ERJ7 | E7ERJ7 | 25.83 | 14 | 15 | 17 | 58 | 0.961 | 0.903 | 0.932 | 0.893 | 0.895 | 0.990 |
| P05141 | ADT2 | 39.26 | 2 | 4 | 12 | 88 | 0.879 | 0.903 | 0.876 | 0.869 | 0.980 | 1.075 |
| C9JL12 | C9JL12 | 4.65 | 2 | 2 | 2 | 2 | 1.030 | 0.903 | 0.876 | 0.960 | 0.930 | 1.058 |
| Q5HY54 | Q5HY54 | 8.63 | 6 | 15 | 17 | 27 | 1.050 | 0.902 | 0.881 | 0.950 | 0.886 | 1.019 |
| Q9Y3F4 | STRAP | 29.14 | 3 | 7 | 7 | 8 | 0.976 | 0.902 | 0.925 | 0.945 | 1.001 | 1.200 |
| Q9NZ63 | CI078 | 14.53 | 2 | 4 | 4 | 5 | 1.046 | 0.902 | 0.873 | 0.914 | 0.807 | 0.982 |
| B7ZB63 | B7ZB63 | 27.08 | 10 | 3 | 5 | 15 | 0.881 | 0.902 | 1.098 | 0.784 | 0.963 | 0.912 |
| E9PF10 | E9PF10 | 3.39 | 3 | 4 | 4 | 9 | 1.070 | 0.902 | 0.899 | 1.047 | 0.993 | 1.182 |
| P61981 | 1433G | 44.53 | 5 | 5 | 11 | 87 | 0.968 | 0.902 | 0.986 | 0.915 | 0.977 | 1.039 |
| P05783 | K1C18 | 20.70 | 2 | 8 | 8 | 8 | 1.049 | 0.902 | 0.882 | 0.768 | 0.754 | 0.871 |
| P27797 | CALR | 25.42 | 2 | 11 | 11 | 103 | 0.954 | 0.902 | 0.955 | 0.973 | 0.979 | 1.054 |
| P30086 | PEBP1 | 26.74 | 2 | 4 | 4 | 14 | 1.054 | 0.902 | 0.906 | 0.972 | 0.988 | 1.074 |
| P45880 | VDAC2 | 20.07 | 5 | 6 | 6 | 33 | 0.957 | 0.901 | 0.962 | 0.993 | 1.051 | 1.061 |
| P31949 | S10AB | 32.38 | 1 | 3 | 3 | 30 | 0.989 | 0.901 | 0.877 | 0.897 | 0.902 | 0.992 |
| Q96I24 | FUBP3 | 5.42 | 2 | 2 | 3 | 6 | 1.009 | 0.901 | 0.978 | 1.049 | 1.054 | 1.141 |
| Q9NSD9 | SYFB | 8.83 | 2 | 5 | 5 | 15 | 1.008 | 0.901 | 0.939 | 0.987 | 0.970 | 0.981 |
| P11021 | GRP78 | 47.09 | 5 | 27 | 30 | 185 | 0.955 | 0.899 | 0.895 | 0.876 | 0.881 | 1.008 |
| Q13442 | HAP28 | 12.15 | 1 | 3 | 3 | 3 | 1.044 | 0.899 | 0.897 | 1.036 | 1.016 | 1.147 |
| P52597 | HNRPF | 23.86 | 3 | 6 | 8 | 56 | 0.961 | 0.898 | 0.920 | 1.014 | 1.028 | 1.089 |
| P61604 | CH10 | 77.45 | 3 | 10 | 10 | 99 | 0.945 | 0.897 | 0.946 | 0.924 | 0.983 | 1.038 |
| Q5VSR7 | Q5VSR7 | 7.73 | 5 | 5 | 6 | 12 | 1.099 | 0.896 | 0.826 | 1.038 | 0.932 | 1.156 |
| P62913 | RL11 | 16.85 | 1 | 3 | 3 | 13 | 0.914 | 0.896 | 0.953 | 0.974 | 1.016 | 1.111 |
| E7ETK0 | E7ETK0 | 29.01 | 3 | 3 | 3 | 54 | 1.016 | 0.895 | 0.834 | 1.077 | 1.051 | 1.231 |
| Q16222 | UAP1 | 3.07 | 2 | 2 | 2 | 13 | 1.015 | 0.895 | 0.871 | 0.934 | 0.841 | 1.087 |
| P30050 | RL12 | 23.64 | 1 | 4 | 4 | 24 | 0.963 | 0.895 | 0.906 | 0.885 | 0.931 | 1.074 |
| F5H0C8 | F5H0C8 | 14.92 | 12 | 2 | 3 | 45 | 1.016 | 0.894 | 0.888 | 1.036 | 1.023 | 1.068 |
| P49327 | FAS | 16.09 | 2 | 29 | 29 | 101 | 0.975 | 0.894 | 0.916 | 0.896 | 0.907 | 0.995 |
| P61088 | UBE2N | 42.76 | 6 | 5 | 5 | 16 | 0.964 | 0.894 | 0.915 | 0.917 | 0.969 | 1.060 |
| C9JGW8 | C9JGW8 | 10.00 | 13 | 3 | 3 | 15 | 0.874 | 0.893 | 1.019 | 0.856 | 0.975 | 0.855 |
| Q9ULW0 | TPX2 | 4.55 | 6 | 3 | 4 | 4 | 1.038 | 0.893 | 0.941 | 0.946 | 1.032 | 1.064 |
| Q9NX63 | CHCH3 | 8.37 | 4 | 2 | 2 | 2 | 0.915 | 0.892 | 0.973 | 0.957 | 1.042 | 1.068 |
| O14737 | PDCD5 | 17.60 | 2 | 2 | 2 | 3 | 1.046 | 0.892 | 0.857 | 0.978 | 0.869 | 1.010 |
| E7EP32 | E7EP32 | 14.19 | 17 | 4 | 4 | 10 | 1.073 | 0.891 | 0.964 | 1.095 | 1.016 | 1.185 |
| P41208 | CETN2 | 17.44 | 1 | 2 | 2 | 2 | 0.964 | 0.891 | 0.923 | 0.955 | 0.988 | 1.067 |
| P63208 | SKP1 | 27.61 | 5 | 4 | 4 | 9 | 0.970 | 0.891 | 0.908 | 0.856 | 0.969 | 1.021 |
| Q15056 | IF4H | 32.26 | 1 | 7 | 7 | 12 | 0.990 | 0.890 | 0.899 | 0.851 | 0.911 | 0.978 |
| Q9Y2W1 | TR150 | 5.55 | 1 | 4 | 4 | 5 | 0.946 | 0.890 | 1.014 | 0.934 | 0.985 | 1.027 |
| Q9Y266 | NUDC | 25.98 | 1 | 8 | 8 | 21 | 1.026 | 0.890 | 0.844 | 0.915 | 0.874 | 1.018 |
| F8WB80 | F8WB80 | 28.40 | 8 | 2 | 2 | 2 | 0.986 | 0.890 | 0.901 | 0.934 | 0.944 | 1.044 |
| P05388 | RLA0 | 29.02 | 14 | 9 | 9 | 54 | 0.948 | 0.890 | 0.966 | 0.939 | 0.920 | 1.027 |
| P43487 | RANG | 15.42 | 8 | 3 | 3 | 23 | 0.881 | 0.889 | 0.995 | 0.845 | 0.964 | 0.956 |
| Q9Y262 | EIF3L | 13.48 | 6 | 8 | 8 | 23 | 1.046 | 0.889 | 0.925 | 0.964 | 0.995 | 1.060 |
| C0IMW5 | C0IMW5 | 12.80 | 4 | 3 | 4 | 4 | 0.937 | 0.888 | 0.897 | 0.990 | 1.053 | 1.109 |
| Q1KLZ6 | Q1KLZ6 | 4.58 | 3 | 2 | 3 | 4 | 0.885 | 0.888 | 1.001 | 0.823 | 0.926 | 0.922 |
| Q9GZP4 | PITH1 | 12.32 | 1 | 2 | 2 | 2 | 0.902 | 0.887 | 0.982 | 0.944 | 1.043 | 1.059 |
| B4DUX0 | B4DUX0 | 8.68 | 3 | 2 | 2 | 13 | 0.898 | 0.887 | 0.884 | 0.989 | 1.096 | 1.200 |
| P11142 | HSP7C | 50.62 | 17 | 26 | 34 | 398 | 0.991 | 0.887 | 0.887 | 0.904 | 0.894 | 1.016 |
| P61163 | ACTZ | 12.50 | 4 | 3 | 4 | 10 | 0.865 | 0.887 | 1.050 | 0.982 | 0.963 | 1.017 |
| Q14320 | FA50A | 6.49 | 2 | 2 | 2 | 2 | 0.741 | 0.885 | 1.192 | 0.706 | 0.950 | 0.794 |
| C9JFR7 | C9JFR7 | 35.64 | 2 | 3 | 3 | 43 | 1.002 | 0.883 | 0.870 | 0.985 | 0.985 | 1.091 |
| C9JBJ6 | C9JBJ6 | 35.09 | 6 | 3 | 3 | 3 | 1.163 | 0.882 | 0.757 | 0.935 | 0.801 | 1.054 |
| Q9H2G2 | SLK | 2.43 | 1 | 2 | 2 | 2 | 0.887 | 0.882 | 0.993 | 1.008 | 1.134 | 1.138 |
| P49591 | SYSC | 13.04 | 2 | 6 | 6 | 34 | 0.874 | 0.882 | 0.984 | 0.825 | 0.921 | 0.931 |
| Q15459 | SF3A1 | 7.82 | 3 | 4 | 4 | 9 | 0.969 | 0.881 | 0.932 | 0.980 | 1.042 | 1.098 |
| B3KTM6 | B3KTM6 | 25.51 | 2 | 6 | 6 | 22 | 1.012 | 0.881 | 0.869 | 1.006 | 1.003 | 1.131 |
| P11388 | TOP2A | 5.09 | 5 | 7 | 7 | 14 | 1.060 | 0.880 | 0.857 | 0.847 | 0.814 | 0.966 |
| P41091 | IF2G | 11.44 | 3 | 5 | 5 | 13 | 0.974 | 0.879 | 0.882 | 1.003 | 1.009 | 1.015 |
| F8VRR3 | F8VRR3 | 7.24 | 8 | 2 | 2 | 3 | 1.054 | 0.879 | 0.872 | 0.947 | 0.821 | 1.157 |
| Q10713 | MPPA | 17.14 | 2 | 6 | 6 | 7 | 0.937 | 0.878 | 0.973 | 0.880 | 0.963 | 1.000 |
| P59998 | ARPC4 | 11.31 | 6 | 2 | 2 | 2 | 0.887 | 0.877 | 0.987 | 1.062 | 1.193 | 1.204 |
| F2Z3A5 | F2Z3A5 | 7.91 | 4 | 3 | 3 | 9 | 1.515 | 0.877 | 0.696 | 1.092 | 0.809 | 1.193 |
| P10768 | ESTD | 13.83 | 1 | 3 | 3 | 8 | 0.993 | 0.876 | 0.992 | 0.989 | 1.042 | 1.113 |
| A6NNE8 | A6NNE8 | 28.48 | 3 | 4 | 5 | 11 | 0.985 | 0.876 | 0.896 | 0.865 | 0.850 | 1.060 |
| F8WEU4 | F8WEU4 | 17.74 | 2 | 2 | 2 | 3 | 1.060 | 0.874 | 0.913 | 0.962 | 0.953 | 0.987 |
| Q96AG4 | LRC59 | 10.75 | 1 | 3 | 3 | 4 | 1.161 | 0.874 | 0.849 | 0.875 | 0.848 | 0.960 |
| Q8N1F7 | NUP93 | 5.13 | 2 | 4 | 4 | 9 | 0.914 | 0.873 | 0.998 | 0.861 | 0.917 | 0.993 |
| Q9UKK9 | NUDT5 | 14.16 | 5 | 2 | 2 | 2 | 0.932 | 0.872 | 0.933 | 0.886 | 0.947 | 1.012 |
| O43776 | SYNC | 9.31 | 4 | 5 | 5 | 8 | 0.896 | 0.872 | 0.957 | 0.944 | 1.064 | 1.131 |
| O00273 | DFFA | 9.97 | 1 | 2 | 2 | 3 | 1.043 | 0.871 | 0.834 | 0.932 | 0.888 | 1.086 |
| P67870 | CSK2B | 7.91 | 3 | 2 | 2 | 4 | 0.978 | 0.871 | 1.070 | 0.975 | 1.006 | 1.031 |
| D6R9P3 | D6R9P3 | 28.93 | 4 | 7 | 7 | 42 | 0.974 | 0.871 | 0.922 | 0.884 | 0.938 | 1.004 |
| Q9Y3C1 | NOP16 | 24.72 | 5 | 4 | 4 | 4 | 0.905 | 0.870 | 0.818 | 0.835 | 0.946 | 1.037 |
| P04264 | K2C1 | 11.18 | 1 | 5 | 7 | 12 | 0.766 | 0.869 | 1.052 | 1.270 | 1.584 | 1.514 |
| P49720 | PSB3 | 16.59 | 1 | 2 | 2 | 20 | 0.866 | 0.869 | 0.954 | 0.909 | 1.017 | 1.103 |
| B7Z800 | B7Z800 | 14.37 | 3 | 3 | 3 | 8 | 0.926 | 0.869 | 1.006 | 1.020 | 1.099 | 1.136 |
| B4DH52 | B4DH52 | 8.16 | 5 | 5 | 5 | 6 | 0.919 | 0.868 | 0.942 | 0.884 | 0.959 | 1.014 |
| O60732 | MAGC1 | 2.19 | 2 | 2 | 2 | 6 | 0.907 | 0.867 | 0.911 | 0.985 | 1.048 | 1.181 |
| P23919 | KTHY | 10.38 | 1 | 2 | 2 | 3 | 0.969 | 0.867 | 0.879 | 0.906 | 0.917 | 1.039 |
| P53618 | COPB | 7.66 | 4 | 6 | 6 | 7 | 1.100 | 0.866 | 0.893 | 1.077 | 0.966 | 1.108 |
| Q9NU22 | MDN1 | 1.13 | 2 | 4 | 4 | 6 | 0.994 | 0.866 | 0.943 | 0.846 | 0.877 | 1.141 |
| Q99848 | EBP2 | 8.82 | 1 | 3 | 3 | 3 | 1.389 | 0.865 | 0.631 | 0.984 | 0.696 | 1.133 |
| Q8TEQ6 | GEMI5 | 3.51 | 1 | 4 | 4 | 6 | 0.986 | 0.864 | 0.844 | 0.862 | 0.995 | 0.976 |
| C9JZI1 | C9JZI1 | 5.95 | 5 | 2 | 2 | 3 | 0.901 | 0.863 | 0.991 | 0.968 | 1.109 | 1.034 |
| P04080 | CYTB | 24.49 | 1 | 2 | 2 | 10 | 0.931 | 0.863 | 0.994 | 1.011 | 1.062 | 1.025 |
| F5H6D0 | F5H6D0 | 7.39 | 3 | 4 | 4 | 7 | 1.001 | 0.863 | 0.877 | 0.858 | 0.900 | 0.999 |
| P62906 | RL10A | 7.37 | 1 | 2 | 2 | 4 | 1.371 | 0.863 | 0.630 | 1.156 | 0.778 | 1.245 |
| P50552 | VASP | 13.42 | 1 | 4 | 4 | 7 | 1.033 | 0.863 | 0.947 | 0.805 | 0.867 | 0.969 |
| B4E318 | B4E318 | 6.11 | 2 | 2 | 2 | 6 | 0.968 | 0.862 | 0.966 | 0.941 | 0.931 | 0.846 |
| P35268 | RL22 | 23.44 | 1 | 3 | 3 | 5 | 1.026 | 0.862 | 0.909 | 0.949 | 0.984 | 1.096 |
| D6RDU5 | D6RDU5 | 8.06 | 12 | 2 | 3 | 3 | 0.881 | 0.861 | 0.976 | 0.869 | 0.983 | 1.004 |
| F2Z2B9 | F2Z2B9 | 2.91 | 4 | 2 | 2 | 2 | 1.110 | 0.861 | 0.774 | 0.809 | 0.726 | 0.934 |
| B4DK30 | B4DK30 | 15.05 | 2 | 11 | 12 | 52 | 0.916 | 0.861 | 0.974 | 0.903 | 0.992 | 1.071 |
| P08107 | HSP71 | 28.86 | 12 | 13 | 19 | 164 | 0.988 | 0.861 | 0.849 | 0.877 | 0.906 | 1.027 |
| O75306 | NDUS2 | 10.15 | 3 | 3 | 3 | 6 | 0.864 | 0.860 | 1.036 | 1.014 | 1.098 | 1.160 |
| P31943 | HNRH1 | 20.27 | 16 | 2 | 6 | 55 | 0.991 | 0.860 | 0.943 | 0.954 | 0.927 | 0.938 |
| P23284 | PPIB | 29.63 | 2 | 7 | 8 | 44 | 0.958 | 0.860 | 0.896 | 0.868 | 0.910 | 1.039 |
| Q15181 | IPYR | 21.45 | 2 | 4 | 4 | 11 | 1.061 | 0.859 | 0.938 | 0.826 | 0.869 | 0.905 |
| O95721 | SNP29 | 28.29 | 2 | 6 | 6 | 14 | 0.937 | 0.859 | 0.957 | 0.915 | 0.960 | 0.969 |
| A6NFM2 | A6NFM2 | 14.56 | 4 | 3 | 3 | 3 | 1.078 | 0.858 | 0.794 | 1.010 | 1.068 | 1.267 |
| E9PKG6 | E9PKG6 | 7.10 | 7 | 2 | 2 | 3 | 0.945 | 0.857 | 0.905 | 0.998 | 1.074 | 1.183 |
| P08670 | VIME | 68.24 | 21 | 30 | 33 | 242 | 1.043 | 0.857 | 0.828 | 0.873 | 0.825 | 0.989 |
| E7ETL9 | E7ETL9 | 27.02 | 2 | 11 | 15 | 50 | 1.028 | 0.856 | 0.814 | 0.889 | 0.810 | 0.994 |
| P62750 | RL23A | 25.00 | 3 | 4 | 4 | 10 | 0.952 | 0.855 | 0.957 | 1.025 | 0.980 | 1.173 |
| O75534 | CSDE1 | 7.27 | 4 | 6 | 6 | 6 | 1.019 | 0.855 | 0.896 | 0.845 | 0.817 | 0.918 |
| P62851 | RS25 | 36.80 | 1 | 5 | 5 | 18 | 1.090 | 0.854 | 0.792 | 1.034 | 0.944 | 1.174 |
| Q8WVM8 | SCFD1 | 6.07 | 9 | 3 | 3 | 3 | 0.809 | 0.854 | 1.081 | 0.787 | 1.020 | 0.955 |
| E9PHJ7 | E9PHJ7 | 2.78 | 4 | 2 | 2 | 2 | 0.904 | 0.853 | 0.943 | 0.961 | 1.060 | 1.120 |
| P62266 | RS23 | 23.78 | 7 | 4 | 4 | 19 | 0.961 | 0.853 | 0.851 | 0.994 | 0.983 | 1.038 |
| Q9NR30 | DDX21 | 16.09 | 3 | 10 | 11 | 35 | 0.993 | 0.853 | 0.908 | 0.959 | 0.967 | 1.126 |
| A6NDN0 | A6NDN0 | 15.67 | 3 | 2 | 2 | 4 | 1.014 | 0.852 | 0.788 | 0.924 | 0.890 | 1.061 |
| P13693 | TCTP | 26.74 | 4 | 4 | 4 | 5 | 0.932 | 0.852 | 0.915 | 0.882 | 0.942 | 1.033 |
| P49006 | MRP | 14.36 | 1 | 2 | 2 | 2 | 0.825 | 0.852 | 1.030 | 0.929 | 1.121 | 1.085 |
| F5H0M0 | F5H0M0 | 6.25 | 3 | 2 | 2 | 2 | 1.015 | 0.851 | 0.849 | 0.777 | 0.774 | 0.909 |
| Q8N8S7 | ENAH | 5.41 | 1 | 3 | 3 | 3 | 0.990 | 0.848 | 0.868 | 0.800 | 0.824 | 0.946 |
| P62072 | TIM10 | 28.89 | 1 | 2 | 2 | 3 | 0.970 | 0.844 | 0.987 | 0.879 | 0.853 | 1.042 |
| B4DS13 | B4DS13 | 6.64 | 10 | 3 | 3 | 4 | 0.929 | 0.841 | 0.934 | 0.792 | 0.850 | 0.907 |
| F8W973 | F8W973 | 2.69 | 4 | 3 | 3 | 4 | 0.991 | 0.839 | 0.862 | 0.751 | 0.787 | 0.910 |
| P62820 | RAB1A | 39.02 | 7 | 3 | 8 | 17 | 0.993 | 0.838 | 0.843 | 0.919 | 0.923 | 1.092 |
| E9PE68 | E9PE68 | 3.66 | 5 | 3 | 3 | 9 | 0.890 | 0.836 | 0.931 | 0.921 | 1.046 | 1.120 |
| Q5SZU1 | Q5SZU1 | 13.03 | 4 | 5 | 5 | 22 | 0.940 | 0.834 | 0.879 | 0.792 | 0.853 | 0.959 |
| E9PC09 | E9PC09 | 6.56 | 4 | 2 | 2 | 3 | 0.693 | 0.833 | 1.298 | 0.982 | 1.425 | 1.175 |
| Q96AT1 | K1143 | 17.53 | 1 | 2 | 2 | 2 | 1.064 | 0.833 | 0.781 | 0.802 | 0.752 | 0.959 |
| F5H2U2 | F5H2U2 | 4.53 | 2 | 3 | 3 | 3 | 0.780 | 0.832 | 1.029 | 0.812 | 1.004 | 0.972 |
| Q13885 | TBB2A | 35.51 | 14 | 2 | 15 | 330 | 0.872 | 0.830 | 1.031 | 0.383 | 0.475 | 0.459 |
| Q9Y3B4 | PM14 | 20.80 | 1 | 2 | 2 | 8 | 1.066 | 0.830 | 0.800 | 0.818 | 0.858 | 1.089 |
| O95747 | OXSR1 | 4.17 | 2 | 2 | 2 | 2 | 0.893 | 0.829 | 0.927 | 0.836 | 0.933 | 1.003 |
| F2Z3F8 | F2Z3F8 | 11.97 | 4 | 2 | 2 | 2 | 1.149 | 0.829 | 0.720 | 0.873 | 0.758 | 1.048 |
| G3V1T2 | G3V1T2 | 3.58 | 2 | 2 | 2 | 2 | 1.066 | 0.829 | 0.776 | 0.745 | 0.696 | 0.895 |
| Q71TU5 | Q71TU5 | 10.46 | 7 | 3 | 3 | 12 | 1.051 | 0.827 | 0.793 | 0.923 | 0.856 | 1.120 |
| Q99614 | TTC1 | 5.82 | 1 | 2 | 2 | 3 | 1.076 | 0.824 | 0.764 | 0.959 | 0.888 | 1.158 |
| F5H6Z3 | F5H6Z3 | 26.77 | 12 | 3 | 8 | 29 | 0.979 | 0.823 | 0.954 | 1.000 | 1.064 | 1.167 |
| G3V4X6 | G3V4X6 | 26.53 | 3 | 2 | 2 | 2 | 0.988 | 0.821 | 0.830 | 0.959 | 0.968 | 1.163 |
| B4DZ67 | B4DZ67 | 5.47 | 4 | 3 | 3 | 7 | 0.936 | 0.818 | 0.936 | 1.215 | 1.308 | 1.393 |
| Q969V3 | NCLN | 4.97 | 1 | 2 | 2 | 2 | 0.892 | 0.816 | 0.913 | 0.734 | 0.821 | 0.896 |
| P84095 | RHOG | 15.18 | 1 | 2 | 2 | 2 | 0.864 | 0.813 | 0.939 | 0.877 | 1.012 | 1.074 |
| Q6UX04 | CWC27 | 9.32 | 2 | 3 | 3 | 3 | 0.862 | 0.813 | 0.942 | 0.975 | 1.035 | 1.037 |
| F8W9Q2 | F8W9Q2 | 5.19 | 3 | 2 | 2 | 4 | 0.846 | 0.811 | 0.957 | 0.911 | 0.914 | 1.138 |
| B5MCY4 | B5MCY4 | 26.96 | 7 | 2 | 2 | 6 | 1.117 | 0.811 | 0.584 | 0.870 | 0.837 | 1.090 |
| B4E2Z3 | B4E2Z3 | 15.26 | 7 | 6 | 6 | 36 | 0.953 | 0.809 | 0.808 | 0.756 | 0.763 | 0.966 |
| P40222 | TXLNA | 3.66 | 1 | 2 | 2 | 2 | 0.966 | 0.807 | 0.834 | 0.918 | 0.948 | 1.133 |
| Q92598 | HS105 | 18.30 | 6 | 11 | 13 | 19 | 0.929 | 0.806 | 0.919 | 0.770 | 0.847 | 0.983 |
| B3KRS5 | B3KRS5 | 13.54 | 10 | 2 | 5 | 14 | 0.893 | 0.806 | 0.901 | 0.901 | 1.006 | 1.113 |
| O60841 | IF2P | 1.64 | 1 | 2 | 2 | 3 | 0.822 | 0.805 | 1.041 | 0.903 | 0.985 | 0.956 |
| P30533 | AMRP | 5.88 | 2 | 2 | 2 | 2 | 0.865 | 0.801 | 0.925 | 0.966 | 1.114 | 1.200 |
| Q9UK45 | LSM7 | 14.56 | 2 | 2 | 2 | 3 | 1.018 | 0.795 | 0.757 | 1.001 | 0.952 | 1.253 |
| E7EX73 | E7EX73 | 6.34 | 14 | 11 | 11 | 36 | 0.962 | 0.795 | 0.904 | 0.882 | 0.983 | 1.067 |
| B4DSE2 | B4DSE2 | 11.95 | 3 | 3 | 3 | 7 | 0.850 | 0.794 | 0.971 | 0.862 | 1.066 | 1.115 |
| O15294 | OGT1 | 2.39 | 2 | 2 | 2 | 3 | 1.171 | 0.794 | 0.677 | 0.810 | 0.689 | 1.010 |
| F5H863 | F5H863 | 5.62 | 2 | 2 | 2 | 2 | 1.067 | 0.784 | 0.733 | 1.027 | 0.959 | 1.303 |
| Q49AN9 | Q49AN9 | 20.31 | 4 | 2 | 2 | 5 | 0.805 | 0.784 | 0.921 | 0.842 | 0.943 | 1.020 |
| P23921 | RIR1 | 8.46 | 4 | 5 | 5 | 12 | 0.916 | 0.773 | 0.870 | 0.974 | 1.029 | 1.065 |
| F5H7R0 | F5H7R0 | 9.34 | 3 | 2 | 2 | 2 | 0.842 | 0.773 | 0.916 | 0.757 | 0.897 | 0.976 |
| E9PCI3 | E9PCI3 | 14.26 | 8 | 6 | 6 | 24 | 0.918 | 0.769 | 0.841 | 0.724 | 0.810 | 0.961 |
| Q9UNQ2 | DIM1 | 6.07 | 2 | 2 | 2 | 2 | 1.158 | 0.768 | 0.662 | 0.983 | 0.846 | 1.274 |
| P42126 | ECI1 | 12.58 | 1 | 3 | 3 | 4 | 0.804 | 0.765 | 1.000 | 0.730 | 1.161 | 1.069 |
| F5H621 | F5H621 | 9.90 | 5 | 2 | 2 | 4 | 1.002 | 0.764 | 0.854 | 0.855 | 0.861 | 1.045 |
| O95486 | SC24A | 2.74 | 1 | 2 | 2 | 5 | 1.096 | 0.764 | 0.981 | 1.173 | 0.951 | 1.267 |
| F8WBG8 | F8WBG8 | 27.34 | 4 | 2 | 2 | 2 | 0.832 | 0.748 | 0.898 | 1.208 | 1.449 | 1.608 |
| O95817 | BAG3 | 3.83 | 2 | 2 | 2 | 3 | 0.883 | 0.747 | 0.948 | 0.813 | 0.917 | 0.999 |
| Q9GZL7 | WDR12 | 4.49 | 1 | 2 | 2 | 2 | 0.968 | 0.739 | 0.762 | 1.061 | 1.093 | 1.429 |
| B7Z844 | B7Z844 | 4.87 | 5 | 2 | 2 | 2 | 1.004 | 0.735 | 0.731 | 0.809 | 0.804 | 1.096 |
| Q96A33 | CCD47 | 12.42 | 1 | 4 | 4 | 5 | 0.861 | 0.730 | 0.846 | 0.902 | 1.078 | 1.368 |
| B3KYB6 | B3KYB6 | 20.83 | 5 | 2 | 2 | 3 | 0.860 | 0.724 | 0.802 | 0.892 | 0.974 | 1.226 |
| Q9Y333 | LSM2 | 28.42 | 1 | 2 | 2 | 10 | 1.515 | 0.704 | 0.463 | 1.277 | 0.865 | 1.859 |
| C9JX83 | C9JX83 | 4.44 | 2 | 2 | 2 | 2 | 1.025 | 0.696 | 0.677 | 0.769 | 0.748 | 1.100 |
| P49790 | NU153 | 2.78 | 2 | 3 | 3 | 4 | 0.721 | 0.693 | 0.879 | 0.913 | 1.095 | 1.041 |
| A8MW61 | A8MW61 | 3.50 | 3 | 2 | 2 | 2 | 1.030 | 0.690 | 0.668 | 0.914 | 0.884 | 1.318 |
| Q8WZA0 | LZIC | 17.37 | 2 | 2 | 2 | 5 | 0.861 | 0.689 | 1.011 | 0.635 | 0.777 | 0.984 |
| P07948 | LYN | 8.40 | 32 | 3 | 4 | 9 | 0.822 | 0.670 | 0.814 | 0.600 | 0.727 | 0.891 |
| P46013 | KI67 | 1.11 | 2 | 3 | 3 | 3 | 1.164 | 0.659 | 0.579 | 0.833 | 0.770 | 1.226 |
| O75794 | CD123 | 8.33 | 1 | 3 | 3 | 9 | 0.886 | 0.644 | 0.739 | 0.726 | 0.824 | 1.121 |
| Q9NSI8 | SAMN1 | 13.40 | 2 | 4 | 4 | 5 | 0.927 | 0.635 | 0.674 | 0.841 | 0.846 | 1.102 |
| Q13618 | CUL3 | 5.08 | 1 | 4 | 4 | 4 | 0.948 | 0.627 | 0.761 | 1.039 | 0.937 | 1.204 |
| Q15758 | AAAT | 4.07 | 1 | 2 | 2 | 4 | 0.738 | 0.590 | 0.672 | 0.628 | 0.714 | 1.059 |
| P67812 | SC11A | 6.70 | 1 | 2 | 2 | 3 | 1.379 | 0.581 | 0.488 | 0.915 | 0.827 | 1.689 |

**Supplementary Table 1. iTraq identification and relative quantification of proteins expressed in not treated and imatinib-treated K562 cells**. Non treated cells (n=2 cell cultures) were labeled with iTRAQ Reagents 117 and 118. Treated cells (n=2 cell cultures) were labeled with iTRAQ Reagents 119 and 121. Only proteins that were identified by 2 peptides or more are listed. Protein ratios were the average of considered peptides ratio. Normalization between conditions was performed based on the protein ration average.
